# Protocol and Statistical Analysis Plan for the Vasopressin for Septic Shock Pragmatic (VASSPR) Cluster-Crossover Randomized Trial

**DOI:** 10.1016/j.chstcc.2025.100178

**Published:** 2025-11-12

**Authors:** Ithan D. Peltan, Danielle Groat, Jason R. Jacobs, Elisabeth H. Tillman, Ben J. Brintz, Stephanie Chauv, Sarah J. Beesley, Jennifer H. Edwards, Natalia Arizmendez, Eliotte L. Hirshberg, Jason R. Carr, Michael J. Lanspa, Joseph R. Bledsoe, Austin T. Smith, Harmony Schneider, Ping Hu, Tamara D. Moores Todd, Carolyn Klippel, Matthew W. Semler, Jonathan W. Casey, Colin K. Grissom, Samuel M. Brown, Lindsay M. Leither

**Affiliations:** Department of Pulmonary and Critical Care Medicine (I. D. P., D. G., J. R. J., E. H. T., S. J. B., J. H. E., N. A., E. L. H., J. R. C., M. J. L., C. K., C. K. G., S. M. B., and L. M. L.), the Department of Pharmacy (S. C. and H. S.), the Department of Emergency Medicine (J. R. B. and T. D. M. T.), Intermountain Medical Center, Murray, the Division of Epidemiology (B. J. B.), Department of Medicine, University of Utah; the Division of Respiratory, Critical Care, and Occupational Pulmonary Medicine (E. L. H., C. K. G., and S. M. B.), Department of Medicine, University of Utah School of Medicine; Digital Technology Services (H. S., P. H., and T. D. M. T.), Intermountain Health, Salt Lake City; the Department of Emergency Medicine (A. T. S.), Park City Hospital, Park City, UT; the Division of Allergy, Pulmonary and Critical Care Medicine (M. W. S. and J. W. C.), Department of Medicine, and the Vanderbilt Institute for Clinical and Translational Research (M. W. S. and J. W. C.), Vanderbilt University Medical Center, Nashville, TN.

**Keywords:** cluster randomization, pragmatic clinical trial, sepsis, septic shock, vasopressin, vasopressor therapy

## Abstract

**BACKGROUND:**

Guidelines recommend vasopressin as the preferred second-line vasopressor for treatment of septic shock. However, the optimal threshold for vasopressin initiation is unclear.

**RESEARCH QUESTION:**

Does a treatment strategy using a lower or a higher threshold for initiation of vasopressin as a secondary vasopressor decrease the incidence of 28-day mortality for patients with septic shock?

**STUDY DESIGN AND METHODS:**

The Vasopressin for Septic Shock Pragmatic (VASSPR) trial is a multicenter, open-label, cluster-randomized, multiple cluster-crossover pragmatic clinical trial being conducted in 13 hospitals in Utah and Idaho. The trial compares a strategy for septic shock management recommending initiation of fixed-dose vasopressin as a secondary vasopressor at a lower threshold (when the total dose of other vasopressors reaches 0.1 mg/kg/min norepinephrine equivalents [NEEs]) to a strategy initiating vasopressin at a higher threshold (when other vasopressors reach 0.4 mg/kg/min NEEs). All adult patients with septic shock at study hospitals are enrolled in the trial and assigned to the treatment strategy in place at the study hospital when and where they start vasopressor treatment for suspected or confirmed septic shock. Individual patient care, including adherence to the assigned vasopressin initiation strategy, occurs at the discretion of each patient’s treating clinicians. The primary outcome is 28-day all-cause mortality. The key secondary outcome is renal replacement therapy-free days to day 28.

**RESULTS:**

This article describes the protocol and statistical analysis plan for the conduct and analysis of the VASSPR trial.

**INTERPRETATION:**

The VASSPR trial will provide important data to guide vasopressor management for patients with septic shock. Prespecification of the trial protocol and statistical analysis plan before completion of enrollment is important for rigorous trial conduct and reporting.

**CLINICAL TRIAL REGISTRATION:**

ClinicalTrials.gov; No.: NCT06217562; URL: www.clinicaltrials.gov

Sepsis is a syndrome of life-threatening organ failure resulting from infection that affects nearly 50 million people around the world each year and contributes to 20% of global deaths.^1,2^ Patients with septic shock comprise approximately 20% of patients with sepsis and show a mortality rate of 30% to 40%.^3–7^ Mortality climbs as high as 80% in refractory shock.^8^

Norepinephrine is the recommended first-line vasopressor in septic shock.^9^ However, higher catecholamine doses result in tachyphylaxis, tachyarrhythmias, lactic acidosis, myocardial toxicity, and immunosuppression.^10–17^ Vasopressin—a potent peripheral vasoconstrictor via V_1_ receptors^18,19^—is a catecholamine-sparing vasopressor of particular interest because patients with septic shock exhibit relative deficiency of this endogenous peptide hormone. International guidelines recommend initiation of fixed-dose vasopressin therapy for patients with an “inadequate” response to norepinephrine^9^ based on meta-analyses suggesting mortality benefit.^9,20^ However, the guidelines do not define an evidence-based threshold for vasopressin initiation, and the provided experience-based suggestion is not aligned with the results of the Vasopressin and Septic Shock Trial, in which the addition of vasopressin to norepinephrine conferred benefit only among patients with less severe shock (norepinephrine < 15 μg/min) at the time of randomization.^21^ As a result of the lack of evidence to inform the threshold at which vasopressin should be initiated, substantial practice variation exists regarding vasopressin use, with some institutions further limiting its administration based on cost.^22–27^

We therefore designed the Vasopressin for Septic Shock Pragmatic (VASSPR) trial as a pragmatic, multicenter, open-label, adaptive, embedded, cluster-randomized, multiple cluster-crossover comparative effectiveness trial to compare 2 strategies for initiation of vasopressin as a secondary vasopressor that fall within the currently observed spectrum of usual care for adult patients with septic shock. The study aims to test the hypothesis that a strategy using a lower threshold for vasopressin initiation reduces mortality for patients with septic shock.

## Study Design and Methods

In this article, we summarize the protocol and statistical analysis plan for the VASSPR trial. Reporting adheres to the Standard Protocol Items: Recommendations for Interventional Trials guidelines and its outcome extension (e-Appendix 1).^28,29^ The full study protocol (e-Appendix 2) and statistical analysis plan (e-Appendix 3) provide additional details on study design, detailed outcome definitions, and statistical analysis methods.

### Trial Design

The VASSPR trial is a pragmatic, multicenter, open-label, adaptive, embedded, cluster-randomized, multiple cluster-crossover comparative effectiveness trial investigating outcomes associated with hospital-level implementation of strategies for septic shock management incorporating a lower threshold (0.1 μg/kg/min norepinephrine or equivalent) vs a higher threshold (0.4 μg/kg/min of norepinephrine or equivalent) for initiation of vasopressin infusion as a secondary vasopressor among patients with septic shock. Conceptually, the trial compares the adoption of 2 different vasopressor management guidelines for septic shock. The trial therefore used a cluster-randomized design employing the types of clinician-targeted implementation methods that are deployed at the hospital level in real-world practice to standardize clinical care around guidelines. The primary outcome is 28-day mortality. The trial is sponsored by Intermountain Health with funding from the Intermountain Foundation and was registered on ClinicalTrials.gov [Identifier: NCT06217562] on January 22, 2024, before the initiation of enrollment on February 1, 2024.

### Trial Setting and Status

The VASSPR trial is enrolling patients at 13 hospitals with adult ICUs—including 1 hospital with 4 adult ICUs—operated by a vertically-integrated health system in Utah and Idaho. Study hospitals include 1 tertiary teaching hospital (vanguard site), 3 regional referral centers, and 9 community hospitals (Table 1 and e-Methods). Study hospitals use norepinephrine’s bitartrate formulation dosed in norepinephrine base units.^30^ Trial enrollment began at a single vanguard hospital on February 1, 2024, and at remaining study sites on June 3, 2024.

### Study Population

All patients meeting study eligibility criteria are enrolled in the study and included in the intention-to-treat analyses, regardless of whether the assigned vasopressor strategy is used by their clinical team (Table 2). Inclusion criteria are:

1. Age ≥ 18 years
2. Admitted to a study hospital emergency department (ED) or inpatient care unit
3. Administration of vasopressor(s) for septic shock-are administered vasopressor(s) for septic shock.

Indications for vasopressor administration documented by the ordering provider in real time within the computerized order for vasopressor administration were used to identify suspected or confirmed septic shock meeting study inclusion criteria (e-Methods). The study has no exclusion criteria. Patients are eligible for enrollment only once during each health care encounter, which merges all contiguous ED and inpatient care at hospitals within the study health system. Patients are re-enrolled if they meet study eligibility criteria during subsequent encounters.

### Randomization and Allocation

**Figure 1 –:**
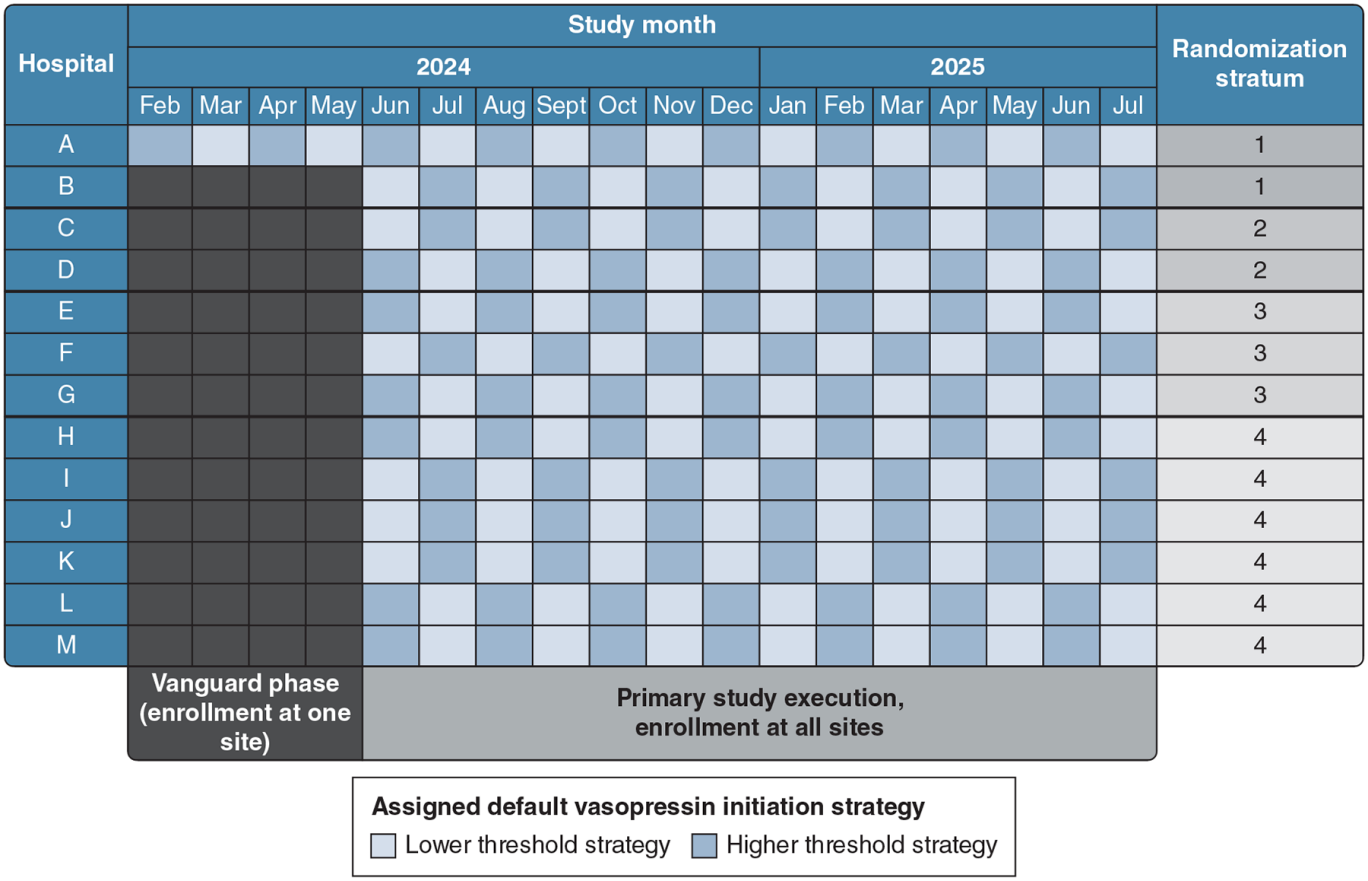
*Randomized hospital strategy assignment during the Vasopressin for Septic Shock Pragmatic trial*.

Before trial initiation, study hospitals’ first enrolling month was assigned randomly to use of a lower- or higher-threshold vasopressin initiation strategy. Randomization used a computer-generated random number sequence and block randomization within 4 strata of study hospitals. Strata were created using historic data on the number of patients with septic shock treated at each hospital to minimize imbalances in the number of patients assigned to each treatment within each period. After the first enrolling month, each study hospital crossed over its treatment strategy monthly through the end of the study (Fig 1) to reduce potential site, temporal, or seasonal effects on measured outcomes.

Participants’ treatment assignment is the vasopressin initiation strategy in place at the time and study hospital they first meet study entry criteria, including being admitted to a study hospital ED or inpatient care unit; having an active order for vasopressors with an indication for suspected or confirmed septic shock; and actively receiving vasopressors. Participants will be analyzed in the group to which they were assigned at the time of initial enrollment, regardless of treatment subsequently received or crossover of the study hospital to the alternative treatment strategy after participants’ study entry.

### Study Intervention

The VASSPR trial compares strategies for septic shock management initiating fixed-dose (0.03 units/min) IV vasopressin as a secondary vasopressor when other vasopressors reach a total norepinephrine equivalent (NEE) dose of 0.1 μg/kg/min (“lower threshold strategy”) or initiating vasopressin when other vasopressors reach a total NEE dose of 0.4 μg/kg/min (“higher threshold strategy”). The 2 treatment strategies were selected to represent approaches to septic shock management within the range of current usual practice both within the study health system and within the United States generally (see e-Methods for additional rationale regarding selection of the thresholds incorporated in the tested strategies). Implementation of vasopressin initiation strategies is embedded in routine care (Table 2), applying at the hospital level clinician-targeted methods to facilitate use of the assigned strategy akin to those used for implementing clinical practice guidelines or evidence-based care protocols. Within this framework, care teams tailor care for individual patients based on clinical judgement as needed, including deciding not to use vasopressin or to initiate vasopressin at an alternative threshold. Implementation and analytic approaches were applied to address unintentional non-adherence to participants’ allocated strategy resulting from such scenarios as transfer between study hospitals assigned to different vasopressor strategies and hospital strategy crossover after trial entry (e-Methods, e-Table 1, and section 5.2.7 of e-Appendix 2).

To facilitate use of study-assigned strategies, we created a study-specific order for threshold-based vasopressin for septic shock that incorporates instructions to initiate the vasopressin infusion on the total dose of first-line vasopressor(s) reaching the assigned threshold value (0.1 μg/kg/min or 0.4 μg/kg/min NEE) (e-Fig 1). Like participants’ treatment assignments, the order’s vasopressin initiation threshold remains stable even if the hospital’s strategy assignment crosses over after order entry. Bedside nurses start vasopressin when the total NEE dose of other vasopressors reaches the relevant threshold. Measures facilitating ordering and adherence to study vasopressor strategies include implementation of an order set for simultaneous ordering of first-line vasopressors (norepinephrine) together with threshold-based vasopressin, making this order the default vasopressor option within ED-oriented and ICU-oriented order sets for sepsis and septic shock management (e-Fig 2), allowing nurses and pharmacists to enter threshold-based vasopressin orders on behalf of clinicians (eg, verbal orders) in situations and settings where this is an option for other vasopressor orders, and deploying a decision support “pop-up” alerts to offer clinicians the opportunity to add a threshold-based vasopressin order when septic shock vasopressors were ordered but no vasopressin order was present and no evidence was present in the electronic medical record (EMR) of a condition potentially affecting the risk to benefit ratio for vasopressin (e-Fig 3 and e-Appendix 2).

### Cointerventions

Sepsis care at the study hospitals uses a shared sepsis management protocol supported by health system-level and hospital-level quality improvement programs.^9,31,32^ No other cointerventions are specified. Clinical care teams determine selection of first-line vasopressors, use of nonvasopressin secondary vasopressors, vasopressor weaning, central vs peripheral vasopressor administration, use of corticosteroids and other adjunctive agents, and IV fluid administration. Placement of central venous access, initiation of stress-dose corticosteroids, and IV fluid administration volumes after enrollment will be reported in the study article.

### Masking

Because of the nature of the study intervention, patients, clinical care teams, and study personnel are not masked to the study intervention. Primary data capture for study outcomes uses automated electronic queries agnostic to treatment assignment.

### Data Collection

Consistent with its pragmatic study design and to minimize potential observer bias, the VASSPR trial is using routinely collected clinical data stored in the study hospitals’ EMR and electronic data warehouse,^33^ including electronic query-based participant identification and capture of core demographic, clinical, laboratory, and outcome data (e-Appendix 4). Mortality after discharge is obtained via linkage to Utah state death records and the US Social Security Death Index. Trained personnel unaware of participants’ strategy assignment use validated, structured methods for review of the medical record^34–37^ to verify vital status, rectify missing data, validate outlier and potentially discrepant data central to participant characterization and data analysis, capture information unavailable from queries (eg, diagnosed source of infection at study entry^34^), identify adverse events, and adjudicate safety outcomes. Data review and entry use the Research Electronic Data Capture^38^ application, including features supporting high-fidelity data capture such as embedded data entry instruction, “piped-in” electronic query data for query-assisted manual adjudication of patient comorbidities and safety outcomes, and real-time error checking. Data are maintained in password-protected, encrypted research computers and servers and are accessible only to approved study personnel.

### Outcomes

The primary outcome is 28-day all-cause, all-location mortality, a patient-centered and objective outcome aligned with prior trials.^14,21,39–41^ The key secondary outcome is renal replacement therapy-free days through day 28, with death on or before day 28 assigned a value of −1.^42^ Exploratory clinical outcomes are hospital and 90-day all-cause mortality^43^; vasopressor-free, ICU-free, and hospital-free days to day 28; and incidence of new renal replacement therapy. Exploratory and safety outcomes are described in detail in Table 3, e-Methods, and sections 1.2.6 through 1.2.8 of e-Appendix 3. Monitored care processes (section 7.2.5 of e-Appendix 2) include those related to strategy implementation—notably the proportion of patients receiving vasopressin after study entry and the maximum dose of nonvasopressin vasopressors (in NEEs) before or without initiation of vasopressin—and interventions potentially influenced by the tested strategies (eg, receipt of stress-dose corticosteroids).

### Statistical Analysis

The primary analysis will compare 28-day all-cause mortality among patients allocated to the 2 treatment strategies—lower vs higher threshold for the addition of vasopressin as a secondary vasopressor—on an intention-to-treat basis. The analysis will use a logistic mixed-effects model including a random intercept for the study hospital and fixed effects for patient-level covariates (age, sex, source of infection at study entry [pulmonary, urine, gastrointestinal or abdominal, skin, or other or multiple], noncardiovascular Sequential Organ Failure Assessment score, chronic kidney disease, chronic cardiovascular disease, and community-acquired vs hospital-acquired septic shock [initiation of vasopressors within 72 hours of hospital arrival]). The treatment effect will be expressed as the adjusted marginal OR and its 95% CI together with the corresponding estimated risk difference and its 95% CI (if applicable),^44,45^ using the higher threshold strategy as the reference (control) arm. A 2-sided *P* value of ≤ .05 for the OR will be considered evidence of a statistically significant effect of the treatment strategy on the outcome. To ensure independence of observations, only the first eligible encounter for an individual patient will be included in analyses.

Analyses of the key secondary and the exploratory and safety outcomes will use methods identical to the primary analysis except for the substitution of ordinal logistic regression for the support-free days outcomes or linear regression for the maximum lactate outcome. Analyses will not be adjusted for multiple comparisons. All exploratory analyses will be considered hypothesis generating.

Exploratory analyses will evaluate whether baseline participant characteristics modify the effect of the vasopressin strategy exposure on the primary outcome. Potential effect modifiers were prespecified (age, sex, infection source, chronic kidney disease, chronic cardiovascular disease, non-cardiovascular Sequential Organ Failure Assessment score, and community- versus hospital-acquired septic shock [initiation of vasopressors within 72 hours of hospital arrival]), with element structure and hypotheses regarding the direction of effect shown in e-Table 2 (section 2.2.7 of e-Appendix 3). Data on the treatment assignment, outcomes, and analysis model covariates are expected to be 100% nonmissing. Section 2.2.3 of e-Appendix 3 describes the approach planned to address unexpected missing data and section 2.2.8 of e-Appendix 3 describes planned sensitivity analyses for the primary and key secondary outcome evaluating potential effects of crossover, temporal trends in treatment and outcomes during the study, participant and encounter eligibility, and statistical modeling strategy.

#### Power Analysis:

Power analyses use estimates for patient volumes, mortality, and within-cluster correlation derived from study hospital historical data. We assumed a 28-day mortality incidence of 33% for patients assigned to the higher vasopressin initiation treatment strategy, including 40% mortality among patients receiving ≥ 0.1 μg/kg/min NEE during the hospitalization (ie, patients expected to be affected directly by the study strategies). Mortality at 28 days was assumed to be 21% for patients receiving < 0.1 μg/kg/min and to be unaffected by strategy assignment. Power analyses used a conservative 16-month study duration (including the 4-month vanguard phase at a single hospital) that was expected to yield 2,050 participants (range, 1–45 participants per month per cluster), including 1,445 participants with a maximum vasopressor dose of ≥ 0.1 μg/kg/min NEE. Assuming within-cluster correlation of 0.003, no heterogeneity of intervention effect across clusters, and a type 1 error rate of 0.05, we estimated the VASSPR trial would have 80% power to detect an absolute 5.4% difference in all-cause mortality (27.6% vs 33.0%) when comparing the lower with the higher initiation threshold treatment strategy.

### Trial Monitoring and Interim Analysis

An independent data and safety monitoring board (DSMB) with expertise in critical care medicine, septic shock, bioethics, biostatistics, and pragmatic clinical trials was convened to monitor study conduct (e-Appendix 5). The study DSMB is performing interim analyses for safety every 6 months, beginning with availability of data from patients enrolled in the trial’s first 6 months. The DSMB conducted the single formal interim analysis for efficacy on January 8, 2025, using data on participants enrolled during the trial’s first 8 months (50% of the 16-month trial duration expected at study launch) and conservative Haybittle-Peto stopping criteria^46–48^ (section 7.6 of e-Appendix 2). Based on this analysis, the DSMB recommended continuing the trial without modification.

Potential adverse effects identified from vasopressin brand name (Vasostrict, Par Pharmaceuticals) and generic package inserts and prior clinical trials are collected and evaluated systematically by the DSMB and in final analyses as safety outcomes (Table 3). Any adverse events determined by a study investigator to be possibly, probably, or definitely related to study procedures are reported to the DSMB and institutional review board, with expedited reporting of such events considered to be serious or unanticipated given the patient’s underlying critical illness and baseline medical conditions and health.^49^ See section 10 of e-Appendix 2 for additional details.

### Study Duration and Adaptation

Trial enrollment began at the vanguard site on February 1, 2024, and at all other sites on June 3, 2024. The trial was designed to close enrollment at each study hospital at a time determined by that hospital’s transition to a new EMR system. At the time of trial design and initiation, enrollment was expected to last 16 months (maximum, 22 months) at the vanguard site and 8 to 12 months at nonvanguard sites (maximum, 18 months). Based on finalized EMR transition plans, the study will close enrollment at the end of July 2025, resulting in total study duration of 18 months at the vanguard site (9 periods per treatment strategy) and 14 months (7 periods per treatment strategy) at other sites, for an overall total of 186 cluster-periods (Fig 1, section 4.5 of e-Appendix 2).

At the 2 scheduled meetings in the first year of the trial, the DSMB reviewed pooled data for factors (eg, enrollment rate) affecting study power. If meaningful differences from expected values were observed, the DSMB could request a re-estimated sample size and could consider recommending trial extension beyond the planned duration (section 7.6 of e-Appendix 2). Based on these reviews, the DSMB did not request a re-estimated sample size or recommend continuing the study beyond the planned duration.

### Ethics

The trial was approved by the Intermountain Health Institutional Review Board (IRB #1052518) and granted a waiver of informed consent given the minimal risk nature of strategy implementation and the impracticability of obtaining informed consent. Section 9 of e-Appendix 2 provides a detailed discussion of the rationale for the consent waiver. In brief, obtaining informed consent was considered impracticable because vasopressors are often started emergently (ie, within minutes of ED arrival or hypotension development), and all patients with septic shock are enrolled in the trial and exposed to the treatment strategy in place at the study hospital at the moment vasopressors are initiated. The study was considered minimal risk because (1) the tested management strategies are within the range of observed usual care; (2) patients’ treating clinicians decide whether to apply the assigned vasopressor management strategy to any individual patient; and (3) the study uses only data collected for routine clinical care. Information about the study was provided to all patients cared for in study hospital ICUs (e-Figs 4–5, section 9.2.4 of e-Appendix 2).

### Patient and Public Involvement

The study health system’s patient and family advisory council for critical care provided feedback on the trial concept, design, and execution (e-Methods).

### Dissemination of Results and Data Sharing

Results of the VASSPR trial will be published in a peer-reviewed journal and entered into ClinicalTrial.gov. Manuscript authorship will adhere to International Committee of Medical Journal Editors criteria. After publication of the trial’s primary results, de-identified datasets generated or analyzed during the VASSPR trial may be requested from the Intermountain Health Office of Research (officeofresearch@imail.org) for use in methodologically sound research after completion of institutional review board review and required data use agreements.

### Protocol Amendments

The study protocol was updated from version 1.0 to version 1.1 on May 17, 2024, to specify norepinephrine dose units per newly-published guidelines,^30^ to clarify methods for support-free day outcome calculation, to apply consistent terminology regarding the study’s investigation of a secondary vasopressor strategy, and to apply formatting required during registration on ClinicalTrial.gov to the naming and description of exploratory outcome.

## Supplementary Material

1

## Figures and Tables

**TABLE 1 ]:** Study Sites

Study Hospital	Location	Description	No. of Hospital Beds	No. of ICU Beds	Intensivist Staffing	Estimated No. of Community-Acquired Septic Shock Cases in 2021
A (vanguard site)	Murray, UT	Tertiary/teaching	472	96	On site	2,200
B	St. George, UT	Regional referral	245	32	On site	1,800
C	Provo, UT	Regional referral	395	36	On site	1,300
D	Ogden, UT	Regional referral	321	16	On site	1,400
E	Salt Lake City, UT	Community	250	14	On site	380
F	Logan, UT	Community	146	12	Telemedicine	580
G	Riverton, UT	Community	97	4	Telemedicine	410
H	Sandy, UT	Community	71	6	Telemedicine	290
I	American Fork, UT	Community	89	8	Telemedicine	670
J	Cedar City, UT	Community	48	6	Telemedicine	280
K	Park City, UT	Community	37	4	Telemedicine	170
L	Burley, ID	Community	25	6	Telemedicine	170
M	Layton, UT	Community	50	4	Telemedicine	330

**TABLE 2 ]:** Vasopressin for Septic Shock Pragmatic Trial Schedule of Events

Activity and Assessment	Study Time Point			
	Allocation and Enrollment	Days 0–28 (or Hospital Discharge)	Day 28	Day 90
Enrollment				
Eligibility screening	X			
Enrollment	X			
Allocation	X			
Interventions				
Initial clinician order for vasopressors for septic shock	X			
Initiation of first-line vasopressors	X			
Clinician discretionary order for threshold-based vasopressin initiation	X	X		
Strategy-related decision support	X	X		
Titration of first-line vasopressors		X		
Initiation of vasopressin		X		
Assessments				
Baseline variables	X			
Adverse events		X		
Cointerventions		X		
Mortality		X	X	X
Secondary or exploratory outcomes		X		
Safety outcomes		X		

**TABLE 3 ]:** Study Outcomes

Outcome Type	Outcome	Description
Primary	28-d all-cause mortality	Death on or before study day 28
Key secondary	Renal replacement therapy-free days to day 28	No. of days between day 28 and the end of the last period of renal replacement therapy before day 28. Death on or before day 28 will be assigned a value of −1.^a,b^
Exploratory	In-hospital all-cause mortality	Death before discharge from the hospital
	90-day all-cause mortality	Death on or before study day 90
	Vasopressor-free days to day 28	No. of days between day 28 and the end of the last period of vasopressor therapy before day 28^a^
	Incidence of new renal replacement therapy	New receipt of renal replacement therapy after enrollment^c^
	ICU-free days to day 28	No. of days between day 28 and the end of the last period of ICU admission before day 28^a^
	Hospital-free days to day 28	No. of days between day 28 and the end of the last period of hospital admission before day 28^a^
Safety	Incidence of acute coronary syndrome^d^	Documented new-onset clinical diagnosis of acute coronary syndrome or ST-elevation or non-ST-elevation myocardial infarction
	Incidence of mesenteric ischemia^d^	Documented new-onset clinical diagnosis of mesenteric ischemia
	Incidence of soft tissue ischemia^d^	Documented new-onset clinical diagnosis of extremity, nose, or ear ischemia
	Incidence of vasopressor extravasation	Documented clinical diagnosis of vasopressor extravasation occurring after onset of septic shock
	Incidence of clinically significant arrhythmia	Documented clinical diagnosis of clinically significant arrhythmia (sustained ventricular tachycardia, re-entrant [supraventricular] tachycardia, atrial arrhythmia with rapid ventricular response requiring intervention, or new-onset atrial fibrillation or flutter) occurring after onset of septic shock
	Incidence of cardiogenic shock	Documented clinical diagnosis of cardiogenic shock occurring after onset of septic shock
	Incidence of cardiac arrest	Documented occurrence of a cardiac arrest with administration of chest compressions or defibrillation after onset of septic shock
	Incidence of severe hyponatremia	New-onset severe hyponatremia (serum sodium < 120 mEq/L)
	Maximum lactate	Maximum lactate (arterial or venous; millimoles per liter) from enrollment through study day 7
	Incidence of abnormal troponin	Serum troponin above upper limit of normal for assay from enrollment through study day 7

a For support-free days outcomes, death on or before day 28 is assigned a value of −1. Detailed definitions are provided in Appendix D of e-Appendix 2.

b Potential values for the renal replacement therapy-free days outcome will be 0 or −1 for patients receiving chronic renal replacement therapy before trial enrollment.

c Patients receiving chronic renal replacement therapy before trial enrollment will be excluded from analyses of the incidence of new renal replacement therapy outcome.

d Participants with onset of the exploratory safety outcome before enrollment in the study will be excluded from analysis of this outcome.

## Interpretation

The VASSPR trial will provide evidence to aid clinicians’ management of septic shock, a common and deadly problem in the ICU. As a result of its pragmatic design and intervention implementation, the trial will yield a generalizable estimate of the effects resulting from implementation of 2 alternative practice guidelines to support more definitive recommendations regarding the threshold for adding vasopressin to first-line vasopressors for septic shock. Submission of the VASSPR trial’s protocol and statistical analysis plan before completion of patient enrollment bolsters transparency and interpretability of the trial’s results.

## Take-Home Points

### Research Question:

Does a treatment strategy using a lower or a higher threshold for initiation of vasopressin as a secondary vasopressor decrease the incidence of 28-day mortality for patients with septic shock?

### Results:

The Vasopressin for Septic Shock Pragmatic (VASSPR) trial is a multicenter, open-label, cluster-randomized, multiple cluster-crossover pragmatic clinical trial being conducted in 13 hospitals. The trial compares a strategy for septic shock management recommending initiation of fixed-dose vasopressin as a secondary vasopressor at a lower threshold (when the total dose of other vasopressors reaches 0.1 mg/kg/min norepinephrine equivalents [NEEs]) with a strategy initiating vasopressin at a higher threshold (when other vasopressors reach 0.4 mg/kg/min NEEs). The primary outcome is 28-day all-cause mortality.

### Interpretation:

The VASSPR trial will address a key knowledge gap affecting clinicians’ management of septic shock, a common and deadly problem in the ICU.

## ABBREVIATIONS:

DSMB: data and safety monitoring board
ED: emergency department
EMR: electronic medical record
MAP: mean arterial pressure
NEE: norepinephrine equivalent
VASSPR: Vasopressin for Septic Shock Pragmatic
