# Supplementary material for: Protocol and Statistical Analysis Plan for the Vasopressin for Septic Shock Pragmatic (VASSPR) Cluster-Crossover Randomized Trial
Source: CHEST Crit Care. Author manuscript; Available in PMC 2026 Jan 17. (PMC12810988; doi:10.1016/j.chstcc.2025.100178)
Supplement: 1 [file NIHMS2130099-supplement-1.pdf]

SUPPLEMENTARY MATERIALS

Protocol and Statistical Analysis Plan for the Vasopressin for Septic Shock Pragmatic (VASSPR) Cluster-Crossover Randomized Trial

Ithan D. Peltan, Danielle Groat, Jason R. Jacobs, Elisabeth H. Tillman, Ben J. Brintz, Stephanie Chauv, Sarah J. Beesley, Jennifer H. Edwards, Natalia Arizmendez, Eliotte L. Hirshberg, Jason R. Carr, Michael J. Lanspa, Joseph R. Bledsoe, Austin T. Smith, Harmony Schneider, Ping Hu, Tamara D. Moores Todd, Carolyn Klippel, Matthew W. Semler, Jonathan W. Casey, Colin K. Grissom, Samuel M. Brown, Lindsay M. Leither

e-Methods ... **2**
ICU staffing trial sites ... 2
Identification of indication for vasopressor administration ... 2
Rationale for selection of vasopressin initiation threshold strategies ... 2
Mitigation of unintentional treatment strategy non-adherence ... 2
e-Table 1. Mitigation of unintentional treatment strategy non-adherence. ... 2
Definitions for support-free days outcomes ... 3
Details of patient and public involvement ... 4
Data access ... 4
e-Figure 1. Order for threshold-based vasopressin ... **5**
e-Figure 2. Integration of threshold-based vasopressin with sepsis order sets ... **6**
e-Figure 3. Vasopressin strategy best practice alert ... **7**
e-Figure 4. Patient/surrogate information poster ... **8**
e-Figure 5. Patient/surrogate information sheet ... **9**
e-Table 2. Baseline variables prespecified for evaluation for effect modification. **10**
e-References ... **11**
e-Appendix 1. SPIRIT 2013 & SPIRIT-Outcomes 2022 combined checklist[7] ... **12**
e-Appendix 2. Study protocol ... **19**
e-Appendix 3. Statistical analysis plan ... **71**
e-Appendix 4. Study variables ... **88**
e-Appendix 5. Data and safety monitoring board charter ... **95**

e-Methods

ICU staffing trial sites
Among the 13 hospitals participating in the Vasopressin for Septic Shock Pragmatic (VASSPR) trial, ICUs at the single tertiary/teaching, three referral hospitals, and one community hospital have 24-hour in-person intensivist coverage. Hospitalists provide in-person coverage of ICUs at the remaining eight community hospitals with support from the study health system's critical care telemedicine program with 24-hour staffing by intensivists and ICU nurses.

Identification of indication for vasopressor administration
For the present pragmatic trial, patients were met the inclusion criterion for "Administration of vasopressor(s) for septic shock" if they had concurrent (1) active vasopressor infusion and (2) an active vasopressor administration order incorporating an indication for septic shock. The indication for vasopressor administration was documented in real time by the ordering clinician within the computerized order for vasopressor administration as a "Yes" or "No" answer to the mandatory field, "Is infection a possible contributor to shock?" Documentation of a "Yes" response indicated the treating clinician was providing treatment for septic shock (suspected or confirmed infection causing or contributing to shock).

Rationale for selection of vasopressin initiation threshold strategies
The vasopressin initiation thresholds implemented in the VASSPR trial were selected to be representative of treatment strategies used in routine clinical care as shown by internal data from the study health system, published data from a nationally-representative group of ICUs, consultation with sepsis experts, and input from patient representative and clinician stakeholders from the study health system's ICUs and EDs. Evidence considered included:

1. The VASST trial,[1] which is the largest trial of vasopressin as a secondary vasopressor to date, suggested improved mortality among patients randomized to vasopressin rather than norepinephrine who started on study drug while norepinephrine was <15 mcg/min (0.19 mcg/kg/min in an 80 kg individual). Though prespecified, this subgroup analysis for heterogeneity of treatment effect must be considered exploratory and underpowered.
2. In a geographically-diverse dataset including 69 U.S. hospitals, risk-adjusted hospital-level initiation thresholds for second-line vasopressors varied by over 10-fold.[2] Roughly 25-30% of hospitals in this study started second-line vasopressors in the vicinity of the lower threshold planned for the present study (norepinephrine 0.1 mcg/kg/min) for an average patient, while approximately 20% of hospitals started second-line vasopressors in the vicinity of the higher threshold planned for the present study (norepinephrine 0.4 mcg/kg/min) for an average patient.
3. Some U.S. health systems restrict vasopressin initiation until other vasopressors reach a norepinephrine equivalent in the range of 0.5 mcg/kg/min. Outside the U.S., vasopressin use is even more restricted.
4. Within the study health system, informal exploratory analysis demonstrated that norepinephrine equivalent vasopressor doses of 0.1 mcg/kg/min and 0.4 mcg/kg/min represented the 24th and 76th percentiles for patient-level vasopressin initiation thresholds after accounting for the characteristics of patients with community-acquired septic shock. Among referral hospitals, the hospital-level median norepinephrine dose at which vasopressin was started ranged from 0.14 to 0.64 mcg/kg/min even after case mix adjustment.

Given feasibility, interpretability, and power considerations and use of vasopressin initiation strategies representative of existing practice within a study design encouraging clinician discretion in the application of treatment strategies, we adopted a two-arm trial design rather than a multi-arm trial design.

Mitigation of unintentional treatment strategy non-adherence
In this pragmatic trial comparing two default strategies for septic shock management, patients' exposure to the assigned treatment strategy begins at study entry and treatment assignment for intention-to-treat analyses will be based on the date and time they meet all inclusion criteria at a study hospital. It is expected and desired that individual participants' vasopressor management will differ from their assigned strategy based on clinician judgement (intentional non-adherence). Multiple strategies were deployed to mitigate risk for *unintentional* strategy non-adherence (e-Table 1).

e-Table 1. Mitigation of unintentional treatment strategy non-adherence.

Selected scenarios	Potential unintentional strategy non-adherence	Key mitigation strategy
Participant enrolled at end of month	Treatment via non-assigned strategy after hospital crosses over to alternate strategy	Once order for threshold-based vasopressin is placed, participant's vasopressin initiation threshold remains stable as long as order is not discontinued; nurse education to "follow the order" for vasopressin initiation threshold
Recurrent septic shock	Treatment via non-assigned strategy during subsequent shock episode if recurrence occurs after hospital crosses over to alternate strategy	Initiation threshold in study order for vasopressin remains stable as long as order is not discontinued
Patient transfer between study hospitals	Transition to non-assigned strategy if destination hospital is assigned to alternate strategy	Education to clinicians about ability to manually order vasopressin initiation threshold based on sending hospital strategy assignment
Readmission during follow-up window	Treatment via non-assigned strategy during subsequent shock episode if recurrence occurs after hospital crosses over to alternate strategy	Subsequent care and outcomes through the end of the index hospital encounter and study follow-up (including any subsequent episodes of shock meeting study entry criteria) contributing to the subjects' study outcomes
Readmission after follow-up window	Treatment via non-assigned strategy if recurrence occurs after hospital crosses over to alternate strategy	For an individual patient, episodes of shock meeting study inclusion criteria that occur during distinct hospital encounters subsequent to the index hospital encounter and primary out will be included in safety monitoring reports for the Data and Safety Monitoring Board and a pre-planned sensitivity analysis but will be excluded from other analyses.
Rapid increase in vasopressor requirement	Failure to initiate vasopressin at assigned threshold	Education to bedside clinicians to start vasopressin "as soon as possible" after ordered threshold reached.
Novel vasopressin strategy implementation	Study vasopressin strategy not used due to lack of awareness	Ongoing multimodal education to clinicians, nurses, and pharmacists; study status review integrated into routine daily care (e.g., nursing huddles); creation of dashboard (updated weekly) depicting crude unit-level order set utilization and strategy adherence.
	Study vasopressin strategy not used due to implementation barriers	Combined order for first-line vasopressors (norepinephrine) together with threshold-based vasopressin created and set as default vasopressor option for septic shock; real-time clinical decision support ("pop up" alert)
Nurse or pharmacist enter vasopressor orders on behalf of clinician	Desired order for threshold-based vasopressin initiation not entered	Nurses and pharmacists educated about study and able to enter threshold-based vasopressin order on behalf of clinician.

Definitions for support-free days outcomes

Support-free day outcomes incorporate the day of study enrollment as study day 0.

- *Renal replacement-free days:* Defined as the number of days alive and off renal replacement therapy — including intermittent hemodialysis, hemofiltration, or ultrafiltration; slow continuous ultrafiltration or dialysis;

or continuous venovenous hemodialysis, hemofiltration, hemodiafiltration, or hemofiltration — from the time of trial enrollment to study day 28.[3,4] If a patient resumes renal replacement therapy and subsequently achieves renal replacement therapy independence prior to day 28, renal replacement therapy-free days will be counted from the end of the last period of renal replacement therapy to day 28 (the "last off" method). Patients discharged alive and off renal replacement therapy will be assumed to remain off through study day 28. Patients discharged alive on renal replacement therapy will be assumed to remain on renal replacement through study day 28. If a patient was receiving renal replacement therapy at day 28 or has end stage renal disease treated with hemodialysis prior to the index hospitalization, renal replacement therapy-free days will be zero. If a patient dies on or before day 28, renal replacement therapy-free days will be -1.[4] The maximum number of renal replacement therapy-free days for a patient is 29 which would be assigned if they were renal replacement therapy independent on the day of study enrollment and remained alive meeting the definition of renal replacement therapy independence to day 28.

- *Vasopressor-free days:* Defined as the number of days alive and off vasopressor support from the time of trial enrollment to study day 28.[3,4] If a patient returns to vasopressor support and subsequently achieves vasopressor independence prior to day 28, vasopressor-free days will be counted from the end of the last period of vasopressor support to day 28 (the "last off" method). Patients discharged alive and off vasopressors will be assumed to remain vasopressor free through study day 28. Patients discharged alive and on vasopressors before day 28 will be assumed to remain on vasopressors through study day 28. A period of vasopressor dependence during a surgical procedure will not count against the vasopressor-free days calculation. If a patient was receiving vasopressors at day 28, vasopressor-free days will be zero. If a patient dies on or before day 28, vasopressor-free days will be -1.[4] The maximum number of vasopressor-free days for a patient is 28 which would be assigned if they were vasopressor independent on the day following study enrollment and remained alive meeting the definition of vasopressor independence to day 28.
- *Hospital-free days:* Defined as the number of days alive and out of the hospital through study day 28. Value of outcome will be calculated analogous to vasopressor-free days, with patients who die on or before study day 28 assigned a value of -1 and patients still hospitalized on day 28 assigned a value of 0. Patients discharged to another acute care hospital (excluding psychiatric inpatient facility) or a long-term acute care facility will be assumed to remain hospitalized through study day 28. Patients discharged alive and who do not die on or before study day 28 will be assumed to remain out of the hospital through day 28.
- *ICU-free days:* defined as the number of days alive and out of the ICU through study day 28. The value of the outcome will be calculated analogous to vasopressor-free days, with patients who die on or before study day 28 assigned a value of -1 and patients still hospitalized on day 28 assigned a value of 0. If a patient returns to the ICU and subsequently achieves ICU independence prior to day 28, ICU-free days will be counted from the end of the last period of ICU care to day 28 (the "last off" method). Patients discharged alive and who do not die on or before study day 28 will be assumed to remain out of the ICU through day 28.

Details of patient and public involvement

The study health system's patient and family advisory council for critical care provided feedback on the trial concept, design, and execution and specific feedback on study outcomes and the protocol-specified methods for protections of participant rights and safety, including the waiver of informed consent and patient-facing communication regarding the study. In addition, two members of this council served as *ad hoc* members of the study protocol committee and provided ongoing input on study design and conduct.
he study principal investigator and study biostatisticians will have access to the study dataset.

Data access

The study principal investigator and study biostatisticians will have access to the study dataset. In addition, any study co-authors may obtain access to the full study dataset.

e-Figure 1. Order for threshold-based vasopressin

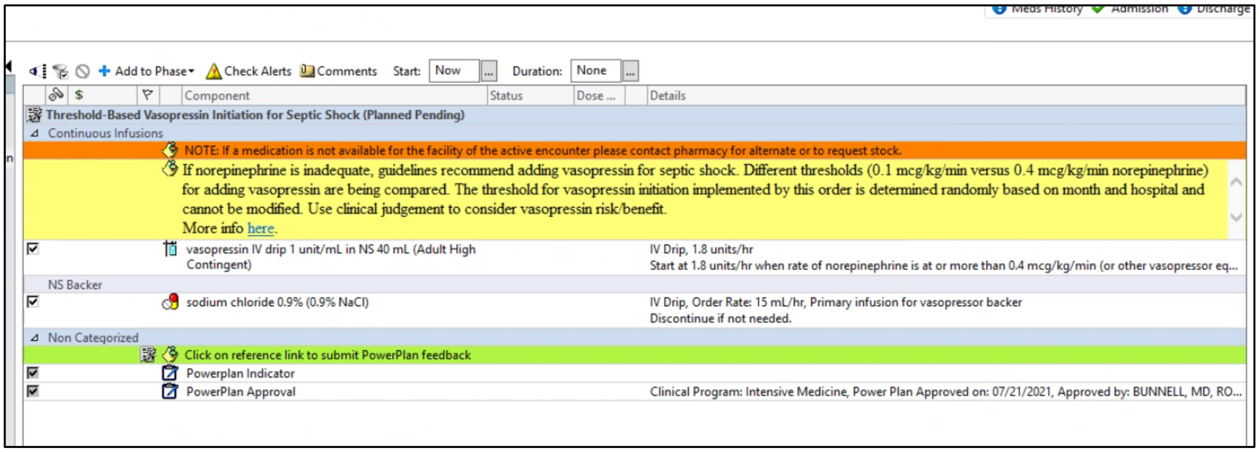

Threshold-based vasopressin orders are placed by clinicians via an order set entitled “Threshold-based Vasopressin Initiation for Septic Shock,” which incorporates either a vasopressin “Adult High Contingent” or “Adult Low Contingent” order with administration instructions to start vasopressin infusion at the corresponding norepinephrine threshold. Inclusion of the vasopressin order incorporating a higher or lower vasopressin initiation threshold is automated based on the date of order entry and patient location. The order set also includes guidance to the ordering clinician regarding the order and a link to additional information about the VASSPR trial.

e-Figure 2. Integration of threshold-based vasopressin with sepsis order sets

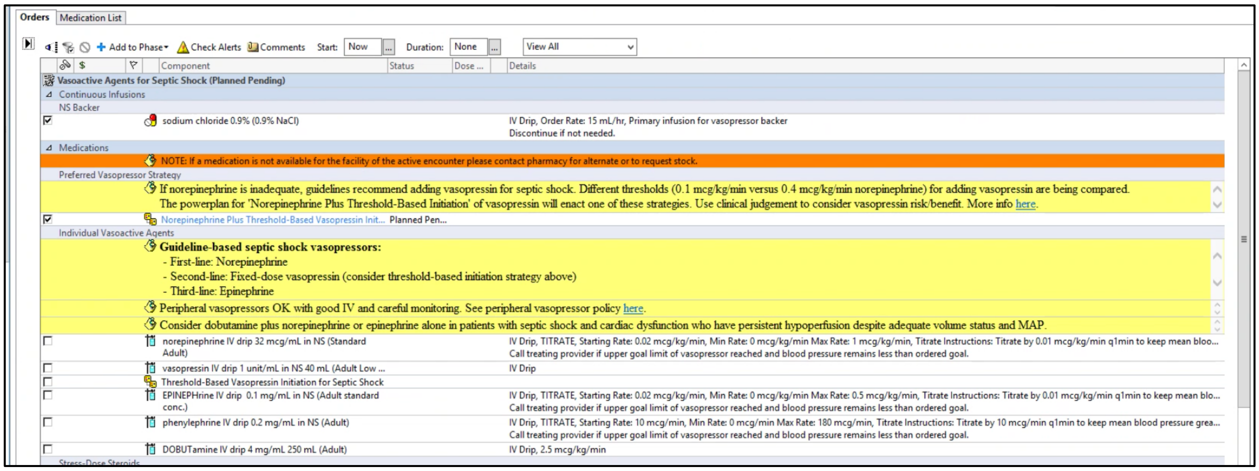

To facilitate clinician ordering of threshold-based vasopressin, an order set combining an order for norepinephrine with an order for threshold-based vasopressin initiation was created and nested within existing ICU and ED vasopressor, sepsis, and septic shock order sets. This example depicts integration of the threshold-based vasopressin order with an ICU-oriented order set for septic shock vasopressors. This order set was presented as the preferred vasopressor strategy alongside guidance to the ordering clinician and a link to additional information about the VASSPR trial. Orders for individual vasopressors as well as the standalone threshold-based vasopressin order continued to be provided within an “individual vasoactive agents” section.

e-Figure 3. Vasopressin strategy best practice alert

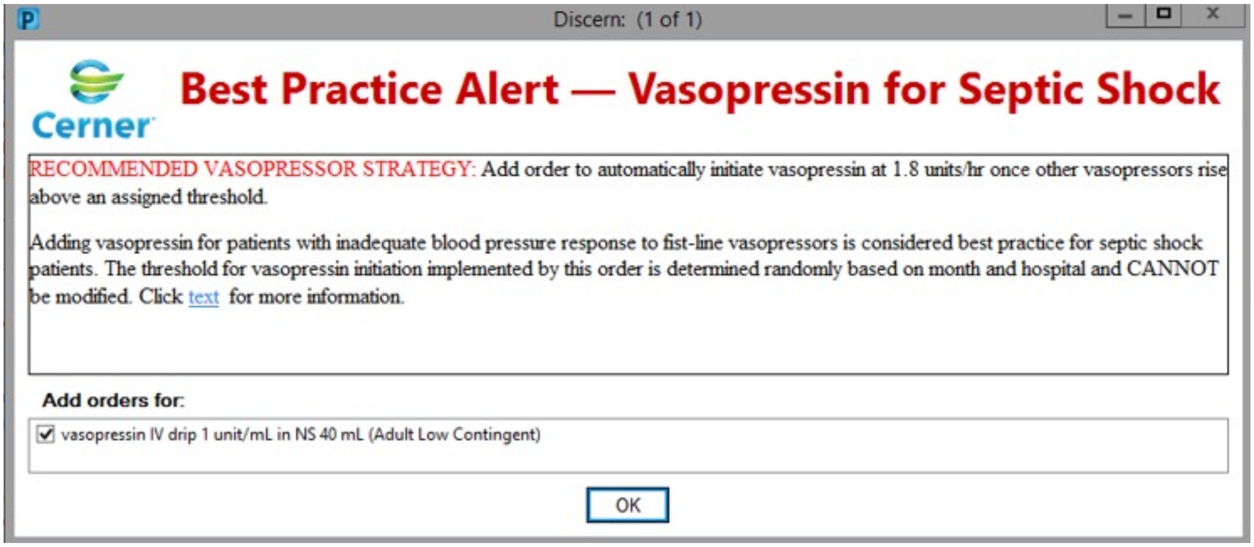

Best practice “pop up” alert provided to clinicians to offer clinicians the opportunity to add a threshold-based vasopressin order when septic shock vasopressors are being ordered but no vasopressin order is present and there is no evidence in the electronic medical record (EMR) of a condition potentially altering the risk/benefit ratio for vasopressin (see Appendix C of e-Appendix 2).

e-Figure 4. Patient/surrogate information poster

VASSPR Trial

Intermountain Health

Intermountain Health is working to understand the best way to care for patients with infection and low blood pressure

Life-threatening low blood pressure due to a serious infection is called “septic shock.” Doctors treat septic shock with medications that raise blood pressure.

What do we need to learn?

Vasopressin is a natural hormone that raises blood pressure. Vasopressin has been used for many years to treat septic shock. Currently, doctors each choose a different time to start vasopressin treatment because the best time to start this medication is not known.

What are we doing?

The doctors, nurses, and pharmacists at Intermountain are conducting a study to learn the best time to start vasopressin for patients with septic shock.

What should you know?

If you have septic shock and your healthcare team does not know when it is best to start vasopressin, the time to start vasopressin may be assigned randomly (like the flip of a coin) based on the month. If the healthcare team already knows when it is best to start vasopressin for you, they will use that approach. No other part of your care will be affected.

Questions

If you have questions, please talk to your doctors or nurses, or call the study project manager (801-507-4791) or Dr. Ithan Peltan (801-507-6556).

e-Figure 5. Patient/surrogate information sheet

VASSPR Trial

Intermountain Health

Vasopressin in Septic Shock Pragmatic Trial (VASSPR)

INFORMATION FOR PATIENTS, FAMILIES, & SURROGATES

What is septic shock?

Life-threatening low blood pressure due to a serious infection is called "septic shock." Shock prevents the brain, kidneys, and other organs from getting enough oxygen.

How is septic shock treated?

Doctors treat septic shock with medications that raise blood pressure. These medications are given through an I.V. and act like adrenaline on the body. The most commonly used medication is "norepinephrine."

What is vasopressin?

Vasopressin is a natural hormone. It is approved for and has been used for many years in addition to norepinephrine to treat septic shock. The best dose level of norepinephrine to add vasopressin for septic shock is not known. Currently, doctors each choose a different level of norepinephrine to start vasopressin.

What is being studied?

This hospital is participating in a clinical trial to help our doctors learn how to treat septic shock better. The trial is comparing two common strategies for treating septic shock:

- Starting vasopressin when other blood pressure raising medications are at a lower level
- Starting vasopressin when other blood pressure raising medications are at a higher level

During the trial, our hospital's treatment strategy alternates each month. Your doctor will choose a different treatment strategy if they feel that is right for you.

Who is included in the trial?

Adult patients treated for septic shock are included in the trial.

What is involved in study participation?

For patients included in the study, members of the research team will confidentially review your medical record. The research team will collect information about you, your medical problems, your treatment, and your hospital stay. No other part of your care will be affected. There will be no cost to you for participating in the study.

How will the trial help me or my loved one?

This study could help improve care of patients with septic shock. It is unknown if one of the treatment strategies is better.

Are there risks to me?

The tested treatment strategies are used for patients at hospitals across the United States. It is not known if risks differ between the treatment strategies. Some potential risks of vasopressin treatment include abnormal heart rhythms and insufficient blood flow to organs and skin. Another possible risk is a loss of privacy of the research data. Intermountain will do everything possible to protect your personal information while conducting this research study.

Questions and concerns

For questions about this trial, please contact:

VASSPR project manager
Pulmonary/Critical Care Research, Bldg 1
Intermountain Medical Center
5121 S Cottonwood St., Murray, UT
(801) 507-4791
VASSPR.trial@imail.org

Ithan Peltan, MD, MSc (principal investigator)
Shock Trauma ICU
Intermountain Medical Center
5121 S. Cottonwood St., Murray, UT
(801) 507-6556
ithan.peltan@imail.org

If you have questions or concerns that you do not feel you can discuss with the study team, please contact Intermountain's Institutional Review Board (IRB) at (**800) 321-2107** or **IRB@imail.org**. The VASSPR trial is registered on the National Institutes of Health ClinicalTrials.gov registry.

e-Table 2. Baseline variables prespecified for evaluation for effect modification.

Potential effect modifier	Format for primary assessment	Categorization of continuous variable for data presentation	Hypothesis regarding effect modification[5]
Age (years)	Continuous	≤65 vs >65 years	No effect modification
Sex at birth	Binary	N/A	No effect modification
Source of infection	Pulmonary, urinary, intrabdominal, or other/multiple	N/A	No effect modification
Chronic kidney disease	Binary	N/A	Reduced mortality benefit from lower threshold for vasopressin initiation among participants with chronic kidney disease compared to patients without chronic kidney disease[6]
Chronic cardiovascular disease	Binary	N/A	No effect modification
Non-cardiovascular SOFA score	Continuous	Tertiles	Reduced mortality benefit from lower threshold for vasopressin initiation among participants with higher non-cardiovascular SOFA score compared to patients with lower non-cardiovascular SOFA score[1]
Time (hours) to vasopressor initiation hospital arrival	Continuous	<72 vs ≥72 hours	No effect modification

e-References

1. Russell JA, Walley KR, Singer J, et al. Vasopressin versus norepinephrine infusion in patients with septic shock. *N Engl J Med.* 2008;358(9):877-887.
2. Bosch NA, Teja B, Wunsch H, Walkey AJ. Practice Patterns in the Initiation of Secondary Vasopressors and Adjunctive Corticosteroids during Septic Shock in the United States. *Ann Am Thorac Soc.* 2021;18(12):2049-2057.
3. Schoenfeld DA, Bernard GR, Network A. Statistical evaluation of ventilator-free days as an efficacy measure in clinical trials of treatments for acute respiratory distress syndrome. *Crit Care Med.* 2002;30(8):1772-1777.
4. Novack V, Beitler JR, Yitshak-Sade M, et al. Alive and ventilator free: a hierarchical, composite outcome for clinical trials in the acute respiratory distress syndrome. *Crit Care Med.* 2020;48(2):158-166.
5. Schandelmaier S, Briel M, Varadhan R, et al. Development of the Instrument to assess the Credibility of Effect Modification Analyses (ICEMAN) in randomized controlled trials and meta-analyses. *CMAJ.* 2020;192(32):E901-E906.
6. Bhatraju PK, Zelnick LR, Herting J, et al. Identification of Acute Kidney Injury Subphenotypes with Differing Molecular Signatures and Responses to Vasopressin Therapy. *Am J Respir Crit Care Med.* 2019;199(7):863-872.
7. Butcher NJ, Monsour A, Mew EJ, et al. Guidelines for Reporting Outcomes in Trial Protocols: The SPIRIT-Outcomes 2022 Extension. *JAMA.* 2022;328(23):2345-2356.

e-Appendix 1. SPIRIT 2013 & SPIRIT-Outcomes 2022 combined checklist[7]

SPIRIT-Outcomes 2022 Checklist (for combined completion of SPIRIT 2013 and SPIRIT-Outcomes 2022 items)[a]

Section	Item No.	SPIRIT 2013 Item	SPIRIT-Outcomes 2022 item	Location Reported[b]
**Administrative information**				
Title	1	Descriptive title identifying the study design, population, interventions, and, if applicable, trial acronym	-	Title page
Trial registration	2a	Trial identifier and registry name. If not yet registered, name of intended registry	-	Title page, abstract, methods
	2b	All items from the World Health Organization Trial Registration Data Set	-	Throughout manuscript, clinicaltrials.gov entry
Protocol version	3	Date and version identifier	-	e-Appendix 2
Funding	4	Sources and types of financial, material, and other support	-	Title page, methods paragraph #1
Roles and responsibilities	5a	Names, affiliations, and roles of protocol contributors	-	Title page
	5b	Name and contact information for the trial sponsor	-	Title page, methods paragraph #1
	5c	Role of study sponsor and funders, if any, in study design; collection, management, analysis, and interpretation of data; writing of the report; and the decision to submit the report for publication, including whether they will have ultimate authority over any of these activities	-	Title page
	5d	Composition, roles, and responsibilities of the coordinating centre, steering committee, endpoint adjudication committee, data management team, and other individuals or groups overseeing the trial, if applicable (see Item 21a for data monitoring committee)	-	e-Appendix 2, e-Appendix 3
**Introduction**				
Background and rationale	6a	Description of research question and justification for undertaking the trial, including summary of relevant studies (published and unpublished) examining benefits and harms for each intervention	-	Introduction
	6b	Explanation for choice of comparators	-	Methods “Study intervention” section; e-Methods
Objectives	7	Specific objectives or hypotheses	-	Introduction

1

Section	Item No.	SPIRIT 2013 Item	SPIRIT-Outcomes 2022 item	Location Reported[b]
Trial design	8	Description of trial design including type of trial (eg, parallel group, crossover, factorial, single group), allocation ratio, and framework (eg, superiority, equivalence, noninferiority, exploratory)	-	Methods “trial design” section
**Methods: Participants, interventions, and outcomes**				
Study setting	9	Description of study settings (eg, community clinic, academic hospital) and list of countries where data will be collected. Reference to where list of study sites can be obtained	-	Methods “trial setting” section, Table 1, e-Appendix 2, ClinicalTrials.gov
Eligibility criteria	10	Inclusion and exclusion criteria for participants. If applicable, eligibility criteria for study centres and individuals who will perform the interventions (eg, surgeons, psychotherapists)	-	Methods “setting” and “trial setting” section
Interventions	11a	Interventions for each group with sufficient detail to allow replication, including how and when they will be administered (for specific guidance see TIDieR checklist and guide)	-	Methods “Study intervention” section, eFigures 1-3, e-Appendix 2 sections 5.2
	11b	Criteria for discontinuing or modifying allocated interventions for a given trial participant (eg, drug dose change in response to harms, participant request, or improving/worsening disease)	-	Methods “Study intervention” section, e-Appendix 2 sections 5.2.7 and Appendix C
	11c	Strategies to improve adherence to intervention protocols, and any procedures for monitoring adherence (eg, drug tablet return, laboratory tests)	-	Methods “Study intervention” section, eFigures 1-3, section 5.2 of e-
	11d	Relevant concomitant care and interventions that are permitted or prohibited during the trial	-	“Co-interventions” section of Methods
Outcomes	12	Primary, secondary, and other outcomes, including the specific measurement variable (eg, systolic blood pressure), analysis metric (eg, change from baseline, final value, time to event), method of aggregation (eg, median, proportion), and time point for each outcome. Explanation of the clinical relevance of chosen efficacy and harm outcomes is strongly recommended	-	Methods outcomes section, Table 3, e-Methods section entitled “Definitions for support-free days outcomes”, and section 7.2

Section	Item No.	SPIRIT 2013 Item	SPIRIT-Outcomes 2022 item	Location Reported[b]
	12.1		Provide a rationale for the selection of the domain for the trial's primary outcome	"Outcomes" section of methods
	12.2		If the analysis metric for the primary outcome represents within-participant change, define and justify the minimal important change in individuals	Not applicable
	12.3		If the outcome data collected are continuous but will be analyzed as categorical (method of aggregation), specify the cutoff values to be used	Table 3 where applicable
	12.4		If outcome assessments will be performed at several time points after randomization, state the time points that will be used for analysis	Table 3
	12.5		If a composite outcome is used, define all individual components of the composite outcome	Table 3 and e-Methods where applicable
Participant timeline	13	Time schedule of enrolment, interventions (including any run-ins and washouts), assessments, and visits for participants. A schematic diagram is highly recommended (see Figure)	-	Table 2
Sample size	14	Estimated number of participants needed to achieve study objectives and how it was determined, including clinical and statistical assumptions supporting any sample size calculations	-	"Power analysis" section of Methods
	14.1		Define and justify the target difference between treatment groups (eg, the minimal important difference)	"Power analysis" section of
Recruitment	15	Strategies for achieving adequate participant enrolment to reach target sample size	-	Not applicable
**Methods: Assignment of interventions (for controlled trials)**				
Allocation:				
Sequence generation	16a	Method of generating the allocation sequence (eg, computer-generated random numbers), and list of any factors for stratification. To reduce predictability of a random sequence, details of any planned restriction (eg, blocking) should be provided in a separate document that is unavailable to those who enrol participants or assign interventions	-	"Randomization and allocation" subsection of Methods

Section	Item No.	SPIRIT 2013 Item	SPIRIT-Outcomes 2022 item	Location Reported[b]
Allocation concealment mechanism	16b	Mechanism of implementing the allocation sequence (eg, central telephone; sequentially numbered, opaque, sealed envelopes), describing any steps to conceal the sequence until interventions are assigned	-	"Randomization and allocation" subsection of Methods
Implementation	16c	Who will generate the allocation sequence, who will enrol participants, and who will assign participants to interventions	-	"Randomization and allocation" subsection of Methods
Blinding (masking)	17a	Who will be blinded after assignment to interventions (eg, trial participants, care providers, outcome assessors, data analysts), and how	-	"Blinding" subsection of Methods
	17b	If blinded, circumstances under which unblinding is permissible, and procedure for revealing a participant's allocated intervention during the trial	-	Not applicable
**Methods: Data collection, management, and analysis**				
Data collection methods	18a	Plans for assessment and collection of outcome, baseline, and other trial data, including any related processes to promote data quality (eg, duplicate measurements, training of assessors) and a description of study instruments (eg, questionnaires, laboratory tests) along with their reliability and validity, if known. Reference to where data collection forms can be found, if not in the protocol	-	"Data collection" subsection of Methods, e-Appendix 4
	18a.1		Describe what is known about the responsiveness of the study instruments in a population similar to the study sample	Not applicable
	18a.2		Describe who will assess the outcome (eg, nurse, parent)	"Data collection" subsection of Methods, e-Appendix 4
	18b	Plans to promote participant retention and complete follow-up, including list of any outcome data to be collected for participants who discontinue or deviate from intervention protocols	-	Not applicable

Section	Item No.	SPIRIT 2013 Item	SPIRIT-Outcomes 2022 item	Location Reported[b]
Data management	19	Plans for data entry, coding, security, and storage, including any related processes to promote data quality (eg, double data entry; range checks for data values). Reference to where details of data management procedures can be found, if not in the protocol	-	"Data collection" subsection of Methods; e-Appendix 2
Statistical methods	20a	Statistical methods for analysing primary and secondary outcomes. Reference to where other details of the statistical analysis plan can be found, if not in the protocol	-	"Statistical analysis" subsection of Methods, e-Appendix 3
	20a.1		Describe any planned methods to account for multiplicity in the analysis or interpretation of the primary and secondary outcomes (eg, coprimary outcomes, same outcome assessed at multiple time points, or subgroup analyses of an outcome)	"Statistical analysis" subsection of Methods, e-Appendix 3
	20b	Methods for any additional analyses (eg, subgroup and adjusted analyses)	-	"Statistical analysis", e-
	20c	Definition of analysis population relating to protocol non-adherence (eg, as randomised analysis), and any statistical methods to handle missing data (eg, multiple imputation)	-	"Statistical analysis" and allocation subsection of Methods; e-Appendix-3
**Methods: Monitoring**				
Data monitoring	21a	Composition of data monitoring committee (DMC); summary of its role and reporting structure; statement of whether it is independent from the sponsor and competing interests; and reference to where further details about its charter can be found, if not in the protocol. Alternatively, an explanation of why a DMC is not needed	-	"Trial monitoring" subsection of methods, section 10 of e-Appendix 2, and e-Appendix 5
	21b	Description of any interim analyses and stopping guidelines, including who will have access to these interim results and make the final decision to terminate the trial	-	Methods - "Trial monitoring"; e-Appendix 2 section 7.6, e-Appendix section 2.1
Harms	22	Plans for collecting, assessing, reporting, and managing solicited and spontaneously reported adverse events and other unintended effects of trial interventions or trial conduct	-	"Trial monitoring" subsection of methods; Table 3; section 10 of e-Appendix 2

Section	Item No.	SPIRIT 2013 Item	SPIRIT-Outcomes 2022 item	Location Reported[b]
Auditing	23	Frequency and procedures for auditing trial conduct, if any, and whether the process will be independent from investigators and the sponsor	-	"Trial monitoring" subsection of methods
**Ethics and dissemination**				
Research ethics approval	24	Plans for seeking research ethics committee/institutional review board (REC/IRB) approval	-	Methods — "Ethics" section
Protocol amendments	25	Plans for communicating important protocol modifications (eg, changes to eligibility criteria, outcomes, analyses) to relevant parties (eg, investigators, REC/IRBs, trial participants, trial registries, journals, regulators)	-	"Protocol amendments" subsection of Methods
Consent or assent	26a	Who will obtain informed consent or assent from potential trial participants or authorised surrogates, and how (see Item 32)	-	Not applicable
	26b	Additional consent provisions for collection and use of participant data and biological specimens in ancillary studies, if applicable	-	Not applicable
Confidentiality	27	How personal information about potential and enrolled participants will be collected, shared, and maintained in order to protect confidentiality before, during, and after the trial	-	Sections 9.2.5 and 9.2.6 of e-Appendix 2
Declaration of interests	28	Financial and other competing interests for principal investigators for the overall trial and each study site	-	Title page
Access to data	29	Statement of who will have access to the final trial dataset, and disclosure of contractual agreements that limit such access for investigators	-	e-Methods
Ancillary and post-trial care	30	Provisions, if any, for ancillary and post-trial care, and for compensation to those who suffer harm from trial participation	-	Not applicable
Dissemination policy	31a	Plans for investigators and sponsor to communicate trial results to participants, healthcare professionals, the public, and other relevant groups (eg, via publication, reporting in results databases, or other data sharing arrangements), including any publication restrictions	-	"Dissemination of results and data sharing" subsection of methods
	31b	Authorship eligibility guidelines and any intended use of professional writers	-	Methods — "Dissemination of results"

Section	Item No.	SPIRIT 2013 Item	SPIRIT-Outcomes 2022 item	Location Reported[b]
	31c	Plans, if any, for granting public access to the full protocol, participant-level dataset, and statistical code	-	"Dissemination of results & data sharing" in methods
**Appendices**				
Informed consent materials	32	Model consent form and other related documentation given to participants and authorised surrogates	-	Not applicable
Biological specimens	33	Plans for collection, laboratory evaluation, and storage of biological specimens for genetic or molecular analysis in the current trial and for future use in ancillary studies, if applicable	-	Not applicable

[a]It is strongly recommended that this checklist be read in conjunction with the SPIRIT (Standard Protocol Items: Recommendations for Interventional Trials) Statement paper for important clarification on the items. Amendments to the protocol should be tracked and dated. The SPIRIT checklist is copyrighted by the SPIRIT Group under the Creative Commons "Attribution-NonCommercial-NoDerivs 3.0 Unported" license and is reproduced with permission.
[b]Indicates page numbers and/or manuscript location: to be completed by authors.

e-Appendix 2. Study protocol

VASSPR Trial

Vasopressin for Septic Shock Pragmatic Trial

STUDY PROTOCOL

Protocol version: Version 1.1

Version date: May 29, 2024

Principal Investigator: Ithan Peltan, MD, MSc

Trial Steering Committee: Ithan Peltan, MD, MSc (chair)
Lindsay Leither, DO
Samuel Brown, MD, MS
Joseph Bledsoe, MD
Colin Grissom, MD

Sponsor: Intermountain Foundation

TABLE OF CONTENTS

1. ABBREVIATIONS AND DEFINITIONS 3
1.1 Abbreviations 3
1.2 Definitions 4
2. PROTOCOL SUMMARY 5
3. STUDY BACKGROUND, SUMMARY, AIMS, HYPOTHESES 7
3.1 Critical illness due to septic shock 7
3.2 First-line therapy for septic shock 7
3.3 Second-line vasopressor options for septic shock 7
3.4 Vasopressin physiology and potential mechanisms of benefit in septic shock 7
3.5 Evidence for vasopressin therapy in septic shock 7
3.6 Practice variation in vasopressin use 8
3.7 Primary objective 9
3.8 Primary hypothesis 9
3.9 Study overview 9
4. STUDY POPULATION AND ENROLLMENT 10
4.1 Setting 10
4.2 Study population 10
4.3 Enrollment 11
4.4 Randomization 11
4.5 Study duration 11
4.6 Treatment assignment 11
4.7 Study population representativeness 11
4.8 Vulnerable subjects 12
5. STUDY PROCEDURES 13
5.1 Study interventions 13
5.2 Pragmatic implementation of study interventions 14
5.3 Vanguard implementation 17
5.4 Standard care 17
5.5 Blinding 17
6. DATA COLLECTION 18
6.1 Data collection 18
6.2 Protected Health Information 18
6.3 Variables/data elements 18
6.4 Data management 20
7. STATISTICAL CONSIDERATIONS AND DATA ANALYSIS 21
7.1 Primary exposure 21
7.2 Outcomes 21
7.3 Data analysis 22
7.4 Missing data 23
7.5 Sample size/power analysis 24
7.6 Interim analysis 24
8. RISK ASSESSMENT 26
8.1 Potential risks 26
8.2 Alternatives to participation 26
8.3 Minimization of risks 27

8.4 Potential benefits of the proposed research to human subjects and others 28
8.5 Importance of the knowledge to be gained 28
9. HUMAN SUBJECTS 29
9.1 Selection of subjects 29
9.2 Waiver of informed consent and HIPAA authorization 29
10. SAFETY AND DATA MONITORING AND REPORTING 32
10.1 Summary and rationale for safety and data monitoring and reporting 32
10.2 Potential Risks and Benefits for Participants 33
10.3 Data and Safety Monitoring Board (DSMB) 33
10.4 Adverse Event Definitions 34
10.5 Adverse Event Monitoring and Reporting 35
10.6 Enrollment, procedure, regulatory, and data quality monitoring 38
10.7 Frequency of data and safety monitoring 38
11. AMENDMENT HISTORY 39
12. BIBLIOGRAPHY 40
APPENDIX A — Eligible vasopressor and associated equivalencies 46
APPENDIX B —Randomized monthly strategy assignments and example trial timeline 47
APPENDIX C — Conditions potentially altering vasopressin risk/benefit ratio 48
APPENDIX D — Definitions of study outcomes and variables 49
Outcomes 49
Other variables 50

1. ABBREVIATIONS AND DEFINITIONS

1.1 Abbreviations

**AE**	Adverse event
**ED**	Emergency Department
**EMR**	Electronic medical record
**HIPAA**	Health Insurance Portability and Accountability Act
**IRB**	Institutional Review Board
**ICU**	Intensive care unit
**ICD-9-CM**	International Classification of Disease, version 9, clinical modification
**ICD-10-CM**	International Classification of Disease, version 10, clinical modification
**DSMB**	Data and Safety and Monitoring Board
**ITT**	Intention to treat
**IV**	Intravenous
**MAP**	Mean arterial pressure
**PI**	Principal investigator
**PHI**	Protected health information
**REDCap**	Research Electronic Data Capture
**SAE**	Serious adverse event
**SOFA score**	Sequential Organ Failure Assessment score
**SpO2**	Oxygen saturation via pulse oximetry
**SUSAR**	Serious and unanticipated suspected adverse reaction
**UP**	Unanticipated problem

1.2 Definitions

- **Adverse event (AE):** Any untoward medical occurrence associated with the use of a drug or a study procedure, whether or not considered drug related.
- **Adverse reaction:** An adverse reaction means any adverse event caused by a study intervention or procedure. An adverse reaction is a subset of all suspected adverse reactions where there is a reason to conclude that the study intervention caused the event.
- **Hypotension**: Systolic blood pressure <90 mmHg or mean arterial pressure (MAP) <65 mmHg or, where applicable, receipt of vasopressor medication.
- **Shock**: Hypotension treated with a continuous infusion of one or more vasopressors.
- **Protected health information (PHI):** identifiable health information that is used, maintained, stored, or transmitted by a HIPAA-covered entity.
- **Serious adverse event (SAE):** Adverse events that meet criteria for seriousness.
- **Sepsis**: Life-threatening organ dysfunction resulting from a dysregulated host response to infection.
- **Septic shock:** Sepsis associated with hypotension treated with a continuous infusion of one or more vasopressors. For purposes of this trial, septic shock will be identified using international consensus criteria receipt of a vasopressor infusion associated temporally with receipt of antimicrobial therapy or a positive test for an infection not amenable to antimicrobial therapy.
- **Suspected adverse reaction**: Any adverse event for which there is a reasonable possibility that the study intervention or procedures caused the adverse event. Reasonable possibility means there is evidence to suggest a causal relationship between the study procedures and the adverse event. A suspected adverse reaction implies a lesser degree of certainty about causality than adverse reaction (21 CFR 312.32(a)).
- **Serious and unanticipated suspected adverse reaction:** Unanticipated adverse events that are also serious and are judged to be possibly, probably, or definitely related to study procedures.

2. PROTOCOL SUMMARY

**Title**	Vasopressin in Septic Shock Pragmatic (VASSPR) Trial
**Background**	Septic shock is a common trigger for intensive care unit admission associated with substantial morbidity and mortality. Therapy includes prompt initiation of appropriate antibiotics, rapid source control, and hemodynamic resuscitation with intravenous fluid and vasopressors. However, the threshold at which to start secondary vasopressor support for patients who receive escalating doses of first-line vasopressors remains unclear.
**Primary objective**	To compare the effect of strategies for septic shock management employing lower versus higher thresholds for initiation of vasopressin infusion as a secondary vasopressor on 28-day mortality among patients with septic shock.
**Primary hypothesis**	A management strategy incorporating a lower threshold rather than a higher threshold for adding vasopressin as a secondary vasopressor will improve 28-day all-cause mortality among patients with septic shock when compared to a higher threshold for adding vasopressin.
**Study design**	Pragmatic multicenter open-label adaptive embedded cluster-randomized cluster-crossover comparative effectiveness trial
**Study treatments**	(1) Lower-threshold for vasopressin initiation strategy: If clinical team places a study-specific threshold-based vasopressin initiation order, instruction to initiate fixed-dose vasopressin infusion (1.8 units/hr) if combined norepinephrine-equivalent dose of other vasopressors reaches ≥0.1 mcg/kg/min.<br>(2) Higher-threshold for vasopressin initiation strategy: If clinical team places a study-specific threshold-based vasopressin initiation order, instruction to initiate fixed-dose vasopressin infusion (1.8 units/hr) if combined norepinephrine-equivalent dose of other vasopressors reaches ≥0.4 mcg/kg/min.
**Randomization**	Cluster-randomized cluster-crossover trial in which each hospital will be randomly assigned during the initial study month to a treatment strategy with subsequent crossover of their treatment strategy monthly.
**Inclusion criteria**	1. Age ≥18 years<br>2. Admitted to a study hospital emergency department (ED) or inpatient care unit<br>3. Administration of vasopressor(s) for septic shock

**Exclusion criteria**	None
**Primary outcome**	28-day all-cause mortality
**Key secondary outcome**	Renal replacement therapy-free days to day 28
**Exploratory clinical outcomes**	In-hospital all-cause mortality<br>90-day all-cause mortality<br>ICU-free days to day 28<br>Hospital-free days to day 28<br>Vasopressor-free days to day 28<br>New receipt of renal replacement therapy after enrollment
**Exploratory safety outcomes**	Clinical diagnosis of new-onset acute coronary syndrome<br>Clinical diagnosis of new-onset mesenteric ischemia<br>Clinical diagnosis of new-onset extremity, nose, or ear ischemia<br>Clinical diagnosis of vasopressor extravasation<br>Clinical diagnosis of clinically-significant arrhythmia<br>Clinical diagnosis of cardiogenic shock<br>Cardiac arrest<br>New-onset severe hyponatremia (serum sodium <120 mEq/L)<br>Maximum lactate through day 7<br>Serum troponin above upper limit of normal through day 7
**Analysis**	Multivariable regression including a fixed effect for treatment assignment, fixed effects adjusting for patient characteristics, and a random effect for study hospital.
**Estimated study duration**	16 months (up to 22 months)
**Estimated patient sample size**	2050 patients (maximum 7000)

3. STUDY BACKGROUND, SUMMARY, AIMS, HYPOTHESES

3.1 Critical illness due to septic shock

Sepsis is a deadly and common syndrome that results when a maladaptive immune response to infection results in organ failure.[1,2] Across the U.S., sepsis results in at least 1.3 million hospitalizations annually associated with 15-20% mortality and over $23 billion in costs for Medicare alone.[2-5] Survivors suffer substantial disability and frequent rehospitalization.[6,7] Septic shock — defined by receipt of vasopressor support to achieve adequate blood pressure and maintain end-organ perfusion[8] — is particularly deadly. The 17% of septic patients with septic shock suffer even worse outcomes than sepsis patients generally, with mortality above 40% in some studies.[2,4,9]

3.2 First-line therapy for septic shock

Good quality evidence supports use of a titrated continuous infusion of norepinephrine as the first-line vasopressor to support target mean arterial pressure (MAP) ≥65 mmHg for sepsis patients with hypotension unresponsive to fluid resuscitation.[10-14] However, concerns persist about toxicities associated with catecholamine therapy for septic shock, including potential modulation of the immune response.[15-17] Such concerns may be particularly relevant for patients with more severe shock, who receive more than low-dose norepinephrine (i.e., >0.1 mcg/kg/min[a]) to maintain target MAPs.

3.3 Second-line vasopressor options for septic shock

For patients in whom norepinephrine monotherapy proves insufficient to achieve MAP targets, U.S. clinicians may choose among five alternative intravenous (IV) agents targeting three different receptor classes:

- Other catecholamines, including dopamine, epinephrine, and phenylephrine;
- Angiotensin II;[19,20]
- Vasopressin (analogues including selepressin and terlipressin are not available with labelled indication for shock in the U.S. market as of May 2023).

International guidelines and the Intermountain Health sepsis care process model recommend vasopressin as the preferred second-line vasopressor in septic shock.[10]

3.4 Vasopressin physiology and potential mechanisms of benefit in septic shock

Vasopressin, an endogenous peptide hormone synthesized in the hypothalamus and secreted from the posterior pituitary, is involved in regulation of plasma osmolality and fluid balance via V_2 receptors. At higher doses, however, the hormone acts a potent vasoconstrictor via V_1 receptors.[21] After a rapid increase early in septic shock, however, vasopressin levels appear to be relatively low in patients with established septic shock compared to other forms of shock.[22] Many experts believe that this represents a vasopressin deficiency in septic shock and suggest that supplementation with exogenous vasopressin may improve outcomes by avoiding catecholamine-induced tachyarrhythmias, myocardial damage, lactic acidosis, immunosuppression, and the tachyphylaxis sometimes observed with high-dose catecholamines.[15,16,23-27]

3.5 Evidence for vasopressin therapy in septic shock

Most trials of vasopressin in septic shock have been underpowered[28-31] or tested vasopressin as a first-line vasopressor.[32,33] The best available evidence comes from the Vasopressin and Septic Shock Trial

[a] References to norepinephrine and norepinephrine-equivalent doses employ norepinephrine base units.[18]

(VASST), which randomized 778 patients with septic shock receiving at least 5 µg/min norepinephrine to initiation of vasopressin (up to 1.8 units/hr) or norepinephrine alone. Randomization was stratified by severity of shock with patients randomized in the less severe stratum if the patient's norepinephrine dose was 5 to 14 µg/min in the hour prior to randomization and the more severe stratum if the norepinephrine dose was 15 µg/min or higher. The trial did not show benefit from vasopressin overall (28-day mortality 39% vs 35%; p=0.26), but there was possible benefit from vasopressin among patients with less severe shock (28-day mortality 27% vs 36%, p=0.05). A formal analysis of effect modification by severity of shock did not achieve statistical significance (p=0.10), but that analysis was particularly underpowered.[34] Some have argued that these results suggest no benefit from vasopressin,[35-37] while others have suggested that vasopressin may have benefit if started earlier in the course of septic shock when norepinephrine doses are lower,[38,39] a hypothesis supported by a recent multihospital observational analysis.[40] No subsequent studies, however, have specifically evaluated the threshold for vasopressin initiation. Renal benefits from use of vasopressin in septic shock suggested by a *post hoc* analysis of the VASST trial[41] and small trials[30,42] have so far not been confirmed in larger trials, though importantly the key trial on this topic used vasopressin as a first-line agent rather than an adjunct to norepinephrine.[33] Overall, while data on vasopressin have led international guidelines to recommend addition of vasopressin infusion for patients "with inadequate MAP levels" on norepinephrine,[10] the guidelines do not indicate a specific threshold at which vasopressin should be started. Instead, they provide a broad "reasonable" range of doses at which vasopressin may be initiated.

3.6 Practice variation in vasopressin use

The lack of evidence to inform the dose of norepinephrine at which vasopressin should be initiated drives dramatic variability in the treatment of septic shock in routine clinical care.[43,44] Further, the lack of definitive evidence of clinical benefit from vasopressin plus rising cost (the price of vasopressin increased 50-fold in 2016) have led many hospitals to alter clinical protocols and restrict the drug's usage despite limited evidence regarding the effect of these changes.[45-48] In an analysis of over 500,000 patients admitted to 532 hospitals, the proportion of hospitals' septic shock patients receiving vasopressin varied from 0 to 70%, with hospital a more important predictor of vasopressin receipt than any patient characteristic except the presence of respiratory failure.[44] Another recent study of 252 U.S. hospitals similarly found 15-fold between-hospital variation in the average dose of norepinephrine at which vasopressin was started, ranging from <10 µg/min to nearly 90 µg/min (Figure 1).[43] Within Intermountain Health, we have observed similar variability in the dose of norepinephrine at which vasopressin is initiated. Among 422 patients with septic shock treated

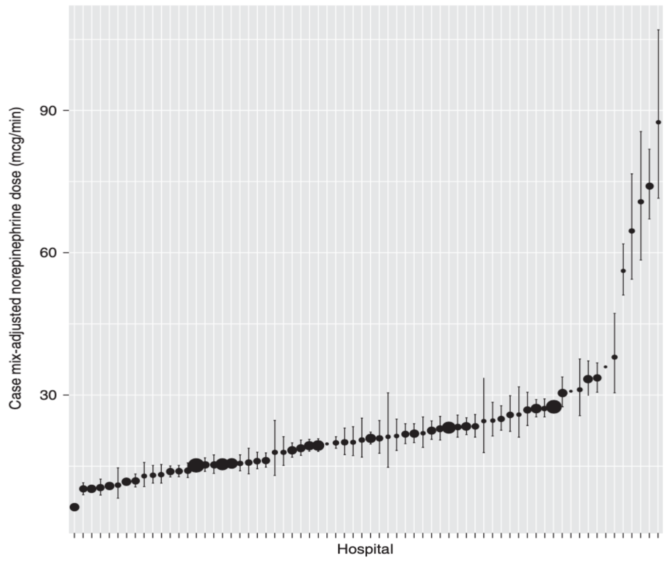

Figure 1. Practice variation for the initiation threshold for second-line vasopressor initiation among 69 U.S. hospitals after case mix adjustment (figure from Bosch *et al.* Annals Am Thorac Soc, 2021; 18: 2049-57).[43]

(VASST), which randomized 778 patients with septic shock receiving at least 5 µg/min norepinephrine to initiation of vasopressin (up to 1.8 units/hr) or norepinephrine alone. Randomization was stratified by severity of shock with patients randomized in the less severe stratum if the patient's norepinephrine dose was 5 to 14 µg/min in the hour prior to randomization and the more severe stratum if the norepinephrine dose was 15 µg/min or higher. The trial did not show benefit from vasopressin overall (28-day mortality 39% vs 35%; p=0.26), but there was possible benefit from vasopressin among patients with less severe shock (28-day mortality 27% vs 36%, p=0.05). A formal analysis of effect modification by severity of shock did not achieve statistical significance (p=0.10), but that analysis was particularly underpowered.[34] Some have argued that these results suggest no benefit from vasopressin,[35-37] while others have suggested that vasopressin may have benefit if started earlier in the course of septic shock when norepinephrine doses are lower,[38,39] a hypothesis supported by a recent multihospital observational analysis.[40] No subsequent studies, however, have specifically evaluated the threshold for vasopressin initiation. Renal benefits from use of vasopressin in septic shock suggested by a *post hoc* analysis of the VASST trial[41] and small trials[30,42] have so far not been confirmed in larger trials, though importantly the key trial on this topic used vasopressin as a first-line agent rather than an adjunct to norepinephrine.[33] Overall, while data on vasopressin have led international guidelines to recommend addition of vasopressin infusion for patients "with inadequate MAP levels" on norepinephrine,[10] the guidelines do not indicate a specific threshold at which vasopressin should be started. Instead, they provide a broad "reasonable" range of doses at which vasopressin may be initiated.

3.6 Practice variation in vasopressin use

The lack of evidence to inform the dose of norepinephrine at which vasopressin should be initiated drives dramatic variability in the treatment of septic shock in routine clinical care.[43,44] Further, the lack of definitive evidence of clinical benefit from vasopressin plus rising cost (the price of vasopressin increased 50-fold in 2016) have led many hospitals to alter clinical protocols and restrict the drug's usage despite limited evidence regarding the effect of these changes.[45-48] In an analysis of over 500,000 patients admitted to 532 hospitals, the proportion of hospitals' septic shock patients receiving vasopressin varied from 0 to 70%, with hospital a more important predictor of vasopressin receipt than any patient characteristic except the presence of respiratory failure.[44] Another recent study of 252 U.S. hospitals similarly found 15-fold between-hospital variation in the average dose of norepinephrine at which vasopressin was started, ranging from <10 µg/min to nearly 90 µg/min (Figure 1).[43] Within Intermountain Health, we have observed similar variability in the dose of norepinephrine at which vasopressin is initiated. Among 422 patients with septic shock treated

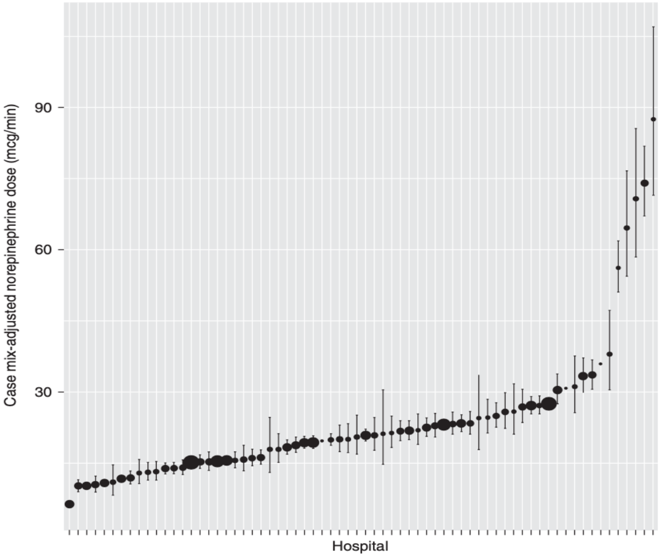

Figure 1. Practice variation for the initiation threshold for second-line vasopressor initiation among 69 U.S. hospitals after case mix adjustment (figure from Bosch *et al.* Annals Am Thorac Soc, 2021; 18: 2049-57).[43]

with vasopressin in 2021, the median norepinephrine dose at which vasopressin was initiated was 0.28 mcg/kg/min, but between-patient variability was significant: 5% of patients started on vasopressin when norepinephrine was ≤0.05 mcg/kg/min and 25% started at or below 0.17 mcg/kg/min of norepinephrine while an additional 25% of patients started vasopressin at or above a norepinephrine dose of 0.45 mcg/kg/min and 16% were not started on vasopressin until the norepinephrine dose was ≥0.60 mcg/kg/min (more than twice the median, Figure 2A). Patient characteristics (age, sex, comorbidity burden, and organ failure severity) were not associated with the norepinephrine dose at which vasopressin was initiated for a particular patient. This variability occurred both within and across the five hospitals within the system where ICU patients are managed by bedside intensivists. After accounting for differences in case mix, the use of vasopressin among patients with septic shock at referral hospitals ranged from 17% to 38% (p<0.001) and the median norepinephrine dose at which vasopressin was initiated ranged from 0.14 to 0.64 mcg/kg/min (p<0.001; Figure 2B). Taken together, these data demonstrate both substantial arbitrary (non-patient motivated) care variation and demonstrate substantial equipoise both nationally and within Intermountain Health with regard to the optimal threshold for vasopressin initiation.

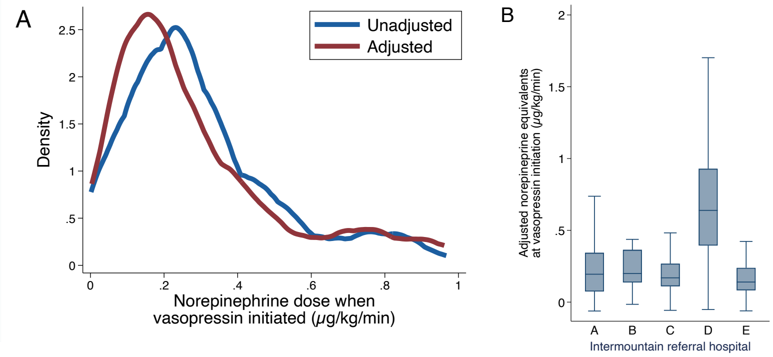

Figure 2. Practice variation at the (A) patient and (B) hospital level for vasopressin initiation threshold among 422 Intermountain Health patients treated with vasopressin for septic shock in 2021.

3.7 Primary objective

To compare the effect of strategies for septic shock management employing lower versus higher thresholds for initiation of vasopressin infusion as a secondary vasopressor on 28-day all-cause mortality among patients with septic shock.

3.8 Primary hypothesis

A management strategy incorporating a lower threshold for adding vasopressin as a secondary vasopressor will improve 28-day all-cause mortality among patients with septic shock when compared to a higher threshold for adding vasopressin.

3.9 Study overview

To compare the effectiveness of alternative strategies currently within the spectrum of usual care for refractory septic shock management, we will conduct a multicenter, open-label, adaptive, cluster-randomized cluster-crossover embedded pragmatic clinical trial (Figure 3). Patients with septic shock in one of Intermountain Health's 13 hospitals in Utah and Idaho with intensive care units (ICUs) during a 2-year period will be included in an analysis comparing clinical and safety outcomes resulting from hospital-level implementation of treatment strategies recommending initiating fixed-dose vasopressin when first-line vasopressors reach a norepinephrine-equivalent infusion rate of 0.1 versus 0.4 µg/kg/min.

4. STUDY POPULATION AND ENROLLMENT

4.1 Setting

Intermountain Health is a nonprofit health system based in Salt Lake City, Utah, with 24 hospitals in Utah and Idaho and 8 additional hospitals in Colorado and Montana. The proposed trial will take place at Intermountain Health's 13 adult hospitals with ICUs in Utah and Idaho. Study hospitals range in size from 25-472 beds and include 1 tertiary teaching hospital, 3 regional referral hospitals, and 9 community hospitals. (Table 1). Study hospitals employ the bitartrate formulation of norepinephrine.[18] Product labeling, clinical administration and documentation of norepinephrine doses use norepinephrine base units. Subsequent references to norepinephrine doses employ norepinephrine base units.

Table 1. Intermountain Health study hospitals.

Hospital	Location	Description	Hospital beds	2021 sepsis cases
Intermountain Medical Center	Murray, UT	Tertiary/teaching	472	2207
St. George Regional Hospital	St. George, UT	Regional referral	245	1819
Utah Valley Hospital	Provo, UT	Regional referral	395	1283
McKay-Dee Hospital	Ogden, UT	Regional referral	321	1405
LDS Hospital	Salt Lake City, UT	Community	250	382
Logan Regional Hospital	Logan, UT	Community	146	578
Riverton Hospital	Riverton, UT	Community	97	406
Alta View Hospital	Sandy, UT	Community	71	291
American Fork Hospital	American Fork, UT	Community	89	673
Cedar City Hospital	Cedar City, UT	Community	48	283
Park City Hospital	Park City, UT	Community	37	173
Cassia Regional Hospital	Burley, ID	Community	25	174
Layton Hospital	Layton, UT	Community	50	328

4.2 Study population

The proposed trial will enroll adult patients (age ≥18 years) admitted to a study hospital with septic shock.[1,2,49]

4.2.1 Inclusion criteria

1. Age ≥18 years
2. Admitted to a study hospital emergency department (ED) or inpatient care unit
3. Administration of vasopressor(s) for septic shock*

   * Determined by concurrent (1) active vasopressor infusion and (2) an active vasopressor administration order incorporating an indication for septic shock. The indication for vasopressor administration is documented by the ordering provider in real time within the computerized order for vasopressor administration. See Appendix A for applicable vasopressors.

4.2.2 Exclusion criteria

There are no exclusion criteria.

4.2.3 Inclusion/exclusion criteria rationale

This study will address vasopressor management of septic shock in adults. The trial will include patients administered vasopressors for whom clinicians indicate a suspected or confirmed diagnosis of septic shock. Children <18 years of age will be excluded because the mechanisms, manifestations, and management of infection and sepsis in children <18 years of age are distinct from those applicable to

adults and vary based on age group between birth and age 18 years. Moreover, septic shock care for children <18 years is performed by distinct care teams in distinct venues using distinct clinical care pathways.

4.3 Enrollment

Patients will be considered enrolled at the time that (1) patient is admitted to a study hospital ED or inpatient care unit; (2) clinician has entered an active order for vasopressors with an indication for suspected or confirmed septic shock; and (3) vasopressors are being infused. Day of enrollment is considered study day 0.

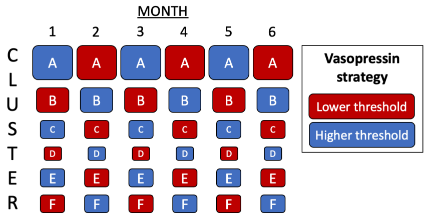

Figure 3. Simplified cluster crossover trial schematic including 6 variably-sized clusters in 6 monthly crossovers. *The actual trial will have 13 clusters and last 24-28 months. See Appendix B for full trial timeline/randomization schematic and study randomization assignments.*

4.4 Randomization

For this cluster-randomized cluster-crossover trial (Figure 3), study hospitals' initial assignment to lower or higher threshold strategy for vasopressin initiation will be determined by block randomization using a computer-generated random number sequence stratified based on four levels of historic hospital septic shock patient volumes. (For one stratum comprised of three hospitals, whether one or two hospitals are initially assigned the low threshold strategy will also be assigned randomly.) Thereafter, hospitals will crossover their treatment strategy monthly through the end of the study (see Appendix B and Sections 4.5 and 5.3).

4.5 Study duration

After a 4-month vanguard phase at one referral hospital (see Section 5.3), active study intervention and enrollment will begin at all study hospitals. The study will continue for approximately 16 months (maximum 22 months) at the vanguard hospital and 6-12 months (maximum 18 months) at each of the other 12 study hospitals. The date on which study hospitals cease study enrollment will be determined by each study hospital's transition from its current electronic medical record (EMR) system to a new EMR.[50-52] (Study hospitals are expected to transition EMRs in late 2025, with some study hospitals acting as pilot sites and transitioning EMRs in spring or summer of 2025. Definite information on each study hospital's EMR transition date is not expected to be available at the time the present trial begins enrollment.) Each study hospital will cease study interventions and patient enrollment preceding that hospital's EMR transition. The hospital's study closure date will be selected such that the amount of time that the hospital is actively enrolling is balanced across the two study arms (i.e., hospitals will have approximately equal time assigned to each vasopressin initiation strategy).

4.6 Treatment assignment

Patients' treatment assignment will be the strategy in place at the time and study hospital they meet all study entry criteria as described in Section 4.3.

4.7 Study population representativeness

Women and individuals of minoritized race and/or ethnicity will be enrolled in proportion to their representation in the population base served by study hospitals, specifically patients presenting to Intermountain Health hospitals located in Utah and Idaho. In order to provide generalizable data, there will be no specific selection criteria that differ between sex/gender and racial/ethnic groups. Hospitals in this study serve a combined population of over 2.9 million individuals, including 24% who are Latino or

Hispanic or of race other than white. We expect the sex, ethnic, and racial mix of the enrolled patients to reflect the population served.

4.8 Vulnerable subjects

The goal of this comparative effectiveness trial is to enroll a cohort that reflects the diversity of the U.S. sepsis population. We therefore anticipate that potentially vulnerable subjects will be eligible for the study, proportional to their membership in the overall population of septic shock patients who will ultimately benefit from the knowledge gained. Specific potentially vulnerable patient subjects include pregnant women, prisoners, and/or individuals with decisional impairment. Importantly, potentially vulnerable participants will not be singled out for inclusion, nor will they disproportionately bear the burden of research unlikely to benefit people like them in the future. Therefore, we believe these individuals should not be excluded from the study since the study is of no more than minimal risk, and in fact, attempting to exclude such individuals from cluster-randomized strategy interventions could increase clinical and privacy risks:

1. In many embedded cluster-randomized effectiveness trials generally and this trial specifically, it is not possible to prevent vulnerable subjects from receiving the study intervention (see Section 5.2) because it is fully integrated with usual care.[53-55] Clinicians will be empowered to tailor usual care to the individual need for each patient with septic shock.
2. The study aims to compare the effects of adopting two different, more specific guidelines for secondary vasopressor initiation in septic shock. Assessment for vulnerability status prior to study entry would preclude achievement of this goal because it would preclude delivery of the intervention via the implementation strategies applied in real-world practice to standardize clinical care based on guidelines.
3. Data on prisoner or incarceration status will not be explicitly collected.
4. Altered mental status is a very common manifestation or complication of sepsis. Excluding patients with decisional impairment would therefore severely bias results of the planned analyses and ultimately preclude obtaining an answer to the study's motivating question.

5. STUDY PROCEDURES

5.1 Study interventions

We will compare two usual care vasopressor management strategies: a lower versus a higher threshold for adding fixed dose vasopressin in septic shock. The two treatment strategies were selected to represent treatment strategies within the range of current usual practice both within the study health system and within the U.S. generally.

5.1.1 Lower-threshold vasopressin strategy

The lower-threshold strategy will target initiation of IV vasopressin at a continuous rate (1.8 units/hr) when the combined dose of other continuously-infused vasopressor medications reaches an equivalent of norepinephrine 0.1 mcg/kg/min.

5.1.2 Higher-threshold vasopressin strategy

The higher-threshold strategy will target initiation of IV vasopressin at a continuous rate (1.8 units/hr) when the combined dose of other continuously-infused vasopressor medications reaches an equivalent of norepinephrine 0.4 mcg/kg/min.

5.1.3 Rationale for selection of vasopressin initiation threshold strategies

The vasopressin initiation thresholds were selected as representative of treatment strategies used in routine clinical care as shown by internal data from the study health system, published data from a nationally-representative group of ICUs, consultation with sepsis experts, and input from patient representative and clinician stakeholders from the study health system's ICUs and EDs. Evidence considered included:

(1) The VASST trial[34] — the largest trial of vasopressin as a second-line vasopressor to date — suggested the possibility of improved outcomes among patients randomized to vasopressin who started on study drug while norepinephrine was <15 mcg/min (0.19 mcg/kg/min in an 80 kg individual), but lacked adequate statistical power to robustly evaluate the question of whether benefit might vary by infusion rate of norepinephrine at the time of vasopressin initiation.

(2) Observational data on current practice in the United States in a geographically-diverse dataset including 69 hospitals demonstrated that risk-adjusted hospital-level initiation thresholds for second-line vasopressors vary by over 10-fold.[43] Roughly 25-30% of hospitals in this study started second-line vasopressors in the vicinity of the lower threshold planned for the present study (norepinephrine 0.1 mcg/kg/min) for an average patient, while approximately 20% of hospitals started second-line vasopressors in the vicinity of the higher threshold planned for the present study (norepinephrine 0.4 mcg/kg/min) for an average patient.

(3) Nationally, some health systems — including Vanderbilt University Medical Center and the 40-hospital University of Pittsburgh system — restrict vasopressin initiation until other vasopressors reach a norepinephrine equivalent in the range of 0.5 mcg/kg/min.

(4) Within the study health system, norepinephrine equivalent vasopressor doses of 0.1 mcg/kg/min and 0.4 mcg/kg/min represented the 17th and 71st percentiles for patient-level vasopressin initiation thresholds, respectively. After accounting for patient characteristics, the selected thresholds represented the 24th and 76th percentiles, respectively (Figure 2A). At the hospital level, median norepinephrine dose at which vasopressin was started ranged from 0.14 to 0.64 mcg/kg/min even after case mix adjustment (Figure 2B).

5.2 Pragmatic implementation of study interventions

Strategy implementation will be embedded in routine care, applying pragmatic methods akin to those applied for evidence-based care protocols, thus encouraging adherence to the assigned strategy as best practice while allowing tailoring of care for individual patients based on clinicians' judgement.

5.2.1 Study-specific threshold-based vasopressin order

A new, study-specific order will be created in the study health system EMR's provider order entry module for threshold-based initiation of fixed-dose IV vasopressin infusion (1.8 units/hour [0.03 units/min]). The order's title will be "Threshold-Based Vasopressin for Septic Shock" or similar, explicitly indicating its intended use is restricted to patients with suspected or confirmed septic shock. Upon this order's entry by the treating clinical team, fixed-dose IV vasopressin infusion will become active medication for the patient, with automatic incorporation of medication administration instructions directing the clinical pharmacy and bedside nurse to initiate the vasopressin infusion when the total dose of first-line vasopressor(s) reaches 0.1 mcg/kg/min (lower threshold) or 0.4 mcg/kg/min (higher threshold) norepinephrine equivalents depending on study treatment assignment currently in place at the hospital where the patient is receiving care.

5.2.2 Methods for ordering the study-specific threshold-based vasopressin order

Clinical teams will be able to place a study-specific threshold-based vasopressin order via multiple pathways, including pathways designed to ease utilization of this order while also ensuring clinical teams are empowered to customize care for individual patients based on clinical judgement. As described in Section 5.2.1, once the study-specific order is placed, the threshold for vasopressin initiation automatically inserted in the order generated will alternate monthly per hospitals' randomized strategy assignment.

5.2.2.1 Direct study-specific threshold-based vasopressin order entry

Licensed clinicians, including attending and resident physicians and advanced practice clinicians will be able to access and order the study-specific threshold-based vasopressin order directly via the provider order entry search function. For key clinician types (e.g., emergency medicine and ICU physicians), the study-specific vasopressin order will be added to their "quick orders" interface. Pharmacists and nurses acting on behalf of a licensed independent physician will also be able to directly access and place the study-specific vasopressin order via their usual methods for entering a "verbal order."

5.2.2.2 Order sets for vasopressor management in septic shock

All existing vasopressor management order sets in the study EMR will be modified to include the study-specific threshold-based vasopressin order described in Section 5.2.1. In order sets specific to sepsis and septic shock, the study-specific vasopressin order will be pre-selected alongside norepinephrine. If clinicians use the septic shock order sets but believe an alternative approach to vasopressin administration is preferred for an individual patient, they may choose to order vasopressors (including vasopressin) outside of the septic shock order set or to modify default orders within the order set, including deactivating the preselected vasopressin or adjusting the ordered vasopressin dose.

5.2.2.3 EMR-embedded decision support

Clinical decision support embedded within the EMR will be deployed to remind clinicians caring for potentially eligible adult patients at a study ED or inpatient unit of the option to enter the order for

threshold-based vasopressin initiation described in Section 5.2.1. The decision support "pop up" alert will appear if (1) clinicians (including pharmacists and nurses acting on behalf of a licensed independent physician) are entering an order for vasopressors; (2) vasopressor orders' designated indication is infection potentially contributing to hypotension; and (3) a vasopressin order is not already present. The alert will incorporate:

1) A reminder that guidelines recommend adding vasopressin in septic shock with an inadequate mean arterial pressure on first-line vasopressors.
2) Two response options:
    a. An option to add the study-specific threshold-based vasopressin order;
    b. An option to continue with the original order without adding the study-specific threshold-based vasopressin order.

The alert will not appear if the patient has evidence in the EMR of a condition (e.g., existing diagnosis of digital ischemia) that could potentially alter the risk/benefit ratio for vasopressin utilization or for a lower or higher vasopressin initiation threshold (see Appendix C). The alert will also be silenced for clinician roles or situations for whom the alert is generally not applicable (e.g., cardiac surgeon).

5.2.4 Treatment delivery based on the study-specific threshold-based vasopressin order

Once a study-specific threshold-based vasopressin order is entered by the clinical team, usual care processes will be used for drug preparation by the clinical pharmacy and administration by the bedside nurse, with the exception that the vasopressin infusion is intended to be initiated only once the patient's other vasopressors reach the ordered threshold.

5.2.4.1 EMR-embedded vasopressin threshold notification for nurses

For patients with an active threshold-based vasopressin order, nurses will receive a notification from the EMR prompting vasopressin initiation when the patient's dose of other vasopressor reaches the assigned/ordered threshold based on real-time entry of vasopressor dose data into the electronic medical record.

5.2.4.2 Other support for threshold-based vasopressin initiation

Implementation strategies customized to the care setting and clinician roles will be utilized to support nursing and pharmacy adherence to the ordered vasopressin initiation threshold. Examples of potential implementation support strategies include:

- Discussion of their hospitals' current assigned threshold for vasopressin initiation during change-of-shift nurse huddles;
- Just-in-time education from ED pharmacists to ED nurses or from ICU telemedicine nurses/pharmacists to bedside ICU nurses triggered by ordering of the threshold-based vasopressin initiation order.

5.2.5 Utilization review and feedback

We will perform selective real-time monitoring of utilization of threshold-based vasopressin orders to allow ongoing support for appropriate utilization and education for clinical care teams.

5.2.6 Education and implementation support for clinical teams

Prior to study launch and periodically throughout the study, we will provide and/or make available education about the trial to clinical personnel — including physicians, advanced practice clinicians,

nurses, and pharmacists — who care for patients with septic shock. This education will be tailored for specific venues and audiences and may include information about current guidelines and data on use of vasopressin in septic shock, conditions and situations that may alter that risk/benefit of vasopressin use, practical guidance about ordering and executing threshold-based vasopressin orders, and education about notifying the study team about adverse events that are possibly related to study procedures. Procedures for ongoing measures to support awareness for physicians, advanced practice clinicians, nurses, pharmacists, and other relevant bedside personnel about the availability and successful utilization of the threshold-based vasopressin orders will be developed and customized as needed to the needs of individual hospitals and care units. Examples of potential strategies include those described in Section 5.2.4.2 as well as:

- Periodic discussion of the trial and utilization rates for the study-specific threshold-based vasopressin initiation order at clinical staff meetings, departmental grand rounds, and other similar venues.
- Just-in-time education from ICU telemedicine clinicians to bedside clinicians regarding the threshold-based vasopressin initiation order when a potentially suitable patient is identified.

5.2.7 Treatment exposure considerations

As described in Sections 4.3 and 4.5, in this pragmatic trial comparing two default strategies for septic shock management, patients' exposure to the assigned treatment strategy begins at study entry and treatment assignment for intention-to-treat analyses will be based on the date and time they meet all inclusion criteria at a study hospital. Patients may not receive vasopressin at the threshold dictated by their assigned treatment strategy for several reasons, including:

- The patients' clinical team does not order vasopressin;
- Vasopressin is initiated by the clinical team at a threshold other than the assigned via an order other than the study-specific threshold-based vasopressin initiation order.
- The clinical team enters the study-specific threshold-based vasopressin initiation order but patient's other vasopressors do not reach the assigned vasopressin initiation threshold.

Once a study-specific threshold-based vasopressin order is entered, the threshold for vasopressin for an individual patient will remain stable as long as that patient remains admitted to the study hospital and the order is not discontinued, even if the study hospital treatment assignment crosses over before patient completes treatment for septic shock. However, some patients assigned to one vasopressin management strategy for analyses may still receive treatment that partially or completely adheres to the alternative vasopressin management strategy in some situations. Examples include:

- Patients who are weaned off vasopressors after initially qualifying for trial inclusion but subsequently have vasopressors reordered after the study site has crossed over to the alternative vasopressin strategy;
- Patients who qualify for trial inclusion while study hospital is assigned to one vasopressin strategy but have a new threshold-based vasopressor order entered after the study site has crossed over to the alternative vasopressin strategy without weaning off vasopressors in the interim;
- Patients transferred between study hospitals randomized to different vasopressin strategies;
- Patients who qualify for trial inclusion, survive the hospitalization, and then have a subsequent readmission to a study hospital involving vasopressor administration for septic shock before the end of study follow up.

Patients will be analyzed in the group to which they were assigned at the time of initial enrollment, regardless of which treatments are subsequently received.

5.3 Vanguard implementation

At a single referral hospital (Intermountain Medical Center), the trial will launch 4 months before system-wide implementation (see Appendix B). During this vanguard phase, study personnel will optimize EMR and other procedures for supporting intervention implementation and adherence, data collection, and safety monitoring. Patients enrolled during the vanguard phase will be included in the intention-to-treat analysis.

5.4 Standard care

Irrespective of study or clinician-assigned vasopressin initiation strategy, all sepsis patients will receive then-current evidence-based care for sepsis as directed and administered by their clinical team. Sepsis care at study hospitals— including antibiotics, procedural source control (where applicable), fluid resuscitation, adjunctive steroid treatment, blood and body fluid cultures, and other aspects of supportive care — is guided by clinician judgment within the context of system-wide protocols and care monitoring for sepsis.[56] Vasopressor and vasopressin weaning will be at the discretion of patients' clinical care team. Key components of supportive care potentially modified by the tested vasopressor management strategies, including fluid resuscitation and adjunctive steroid treatment specifically, will be chosen by the clinical team and included as monitored care processes in study analyses.

5.5 Blinding

Due to the pragmatic design of the trial, blinding of patients, clinicians, and study personnel to treatment assignment is not practicable. Primary data collection for study outcomes, including the primary 28-day mortality outcome, will be obtained via data queries agnostic to treatment assignment, with any manual adjudication of endpoints and other study data also blinded to treatment assignment. A statistician blinded to treatment assignment throughout the trial will conduct final analyses.

6. DATA COLLECTION

Core analyses will employ data collected during routine clinical care and hospital operations. There will be no direct patient contact for collection of identifiable data or specimens.

6.1 Data collection

The Intermountain Health Enterprise Data Warehouse (EDW) is a centrally-managed, well-curated, and accessible database linking system-wide clinical, billing, laboratory, and other data.[57] Trial data managers will identify eligible patient subjects using EDW data and will generate a dataset specific to the proposed study by linking EDW data to additional data abstracted from the electronic health record. We will employ a preexisting linkage to Utah State death records and the U.S. Social Security Death Index for mortality ascertainment. Manual chart review will be employed as needed to validate and supplement electronically-available data and will use the Research Electronic Data Capture (REDCap) platform.[32]

6.2 Protected Health Information

Protected health information (PHI) including subject encounter codes, medical record numbers, birthdates, encounter event date and times, address, and social security number will be collected to allow manual chart abstraction, intervention implementation, manual review of electronic record for data completion and verification, and estimation of patient socioeconomic status and also to ensure accurate capture of mortality outcomes. Specific PHI data elements planned for collection are:

- Patient medical record number(s)
- Patient encounter ID code(s)
- Hospital/facility
- Date of birth
- Name
- Date/time of events related to hospitalization (e.g. outside hospital arrival/departure, ED arrival & departure, hospital admission & discharge), treatments, clinical testing and documentation, and other clinical events
- Date/time of death (if applicable)
- Address/zip code (for determination of residence at a nursing facility or other long-term care facility and estimation of socioeconomic status)
- Age (including age >89 years)

6.3 Variables/data elements

- Demographics
  - Age
  - Sex
  - Race
  - Ethnicity
  - Marital status
  - Preferred language
  - Residence prior to hospital admission (e.g. home, skilled nursing facility, long-term acute care hospital)
  - Insurance status/type

- Characteristics of index hospitalization
    - Type of admission to study hospital (e.g., emergent vs elective)
    - Source of admission to study hospital (e.g., ED of study hospital, ED of other Intermountain Health hospital)
    - Patient transferred from initial study hospital to another acute care hospital (yes/Intermountain Health, yes/other hospital, no)
    - Care location(s)
- Clinical data related to index hospitalization (with associated date/times as applicable)
    - Admission diagnosis
    - Illness severity scores (e.g. APS, APACHE IV score)
    - Comorbidities and comorbidity scores (e.g. Charlson index, Elixhauser index)
    - Chronic dialysis
    - Home medications
    - Acute SOFA score and components
    - Baseline SOFA score and components (up to preceding 10 years)
    - Baseline laboratory data (up to preceding 10 years)
    - Clinical care notes and authors
    - Index hospitalization diagnoses
    - Presence/source of infection diagnosed at key time points (e.g., ED arrival, antibiotic initiation, at time of vasopressor initiation)
    - Presence/source of infection on final assessment
- Clinical assessments (e.g. physical exam, vital signs, triage scores, RASS), laboratory, microbiology, diagnostic, and radiology testing related to index hospitalization
    - Assessment/test type/characteristics
    - Associated date/times
    - Test results
- Antibiotics, vasopressors, IV fluid, and other pharmacologic therapies
    - Pharmacologic agent
    - Route
    - Rate (if applicable)
    - Dose
    - Date/time(s)
    - Caregivers involved in therapy ordering and administration
    - Complications (if applicable)
    - Antibiotic spectrum scores
    - Costs of drug and administration
- Other clinical treatments and interventions (e.g. mechanical ventilation, central line placement, renal replacement therapy)
    - Treatment/intervention performed and characteristics thereof
    - Associated date/times
    - Complications (if applicable)
- Admission to hospital via ED (yes/no)
    - Mode of arrival to ED
    - ED disposition
    - ED length of stay
- Hospitalization outcomes
    - Hospital disposition

- In-hospital all-cause mortality
- Long-term all-cause mortality (e.g. 28-day, 90-day and 1-year mortality)
- Death date
- Healthcare charges, revenue, costs etc.
- Discharge diagnosis and procedure codes
- Diagnosis-related group
- Hospital length of stay
- ICU length of stay
- Post-discharge healthcare utilization (with dates/times and associated diagnoses)

6.4 Data management

Data analysts and research coordinators will collect data and record it in a custom-designed computer database. Data abstraction from electronic medical records will employ either standardized paper case report forms or a custom-designed interface maintained within Intermountain Health's secure Research Electronic Data Capture (REDCap) platform.[32] Outside REDCap, all electronic study data will be kept in a protected database on a protected computer and all paper forms will be kept in secured file storage. Each subject will be assigned a study identification number, and we will maintain minimal patient identifiers. Shared or presented data will be in anonymized or aggregate format such that individual patient subjects cannot be reidentified. An unblinded statistician will collate, analyze, and present relevant study data and other trial issues to the Data and Safety Monitoring Board (DSMB). A statistician blinded to both treatment assignment and data related to monitored care processes (see Section 7.2.4) will perform analyses of primary, secondary, and exploratory outcomes.

7. STATISTICAL CONSIDERATIONS AND DATA ANALYSIS

7.1 Primary exposure

The primary exposure will be the assigned vasopressin treatment strategy. For the primary intention-to-treat (ITT) analysis, study subjects will be treated as having received the treatment strategy in place at the time and hospital they first met all study inclusion criteria.

7.2 Outcomes

The day of study enrollment is considered study day 0. All outcomes are evaluated at the patient level. All-cause mortality outcomes are measured through day 90. All-cause 28-day mortality follow-up is incorporated into measurement of event- or treatment-free days. Other outcomes are censored at hospital discharge unless otherwise specified. All interventions and all laboratory or other diagnostic testing reflected in outcomes will be performed as part of routine clinical care. Additional details of outcome definition are included in the Appendix D.

7.2.1 Primary outcome

The primary outcome will be all-cause 28-day mortality (death on or before study day 28).

7.2.2 Key secondary outcome

The key secondary outcome will be renal replacement therapy-free days to day 28, with death on or before day 28 assigned a value of -1.[58] For patients with baseline end-stage renal failure on dialysis prior to the index hospitalization, potential values for this ordinal outcome will be 0 or -1.

7.2.3 Exploratory clinical outcomes

- In-hospital all-cause mortality
- 90-day all-cause mortality
- Vasopressor-free days to day 28
- New receipt of renal replacement therapy after enrollment (excludes subjects receiving renal replacement therapy prior to enrollment)
- ICU-free days to day 28 (calculated analogous to vasopressor-free days)
- Hospital-free days to day 28 (calculated analogous to vasopressor-free days)

7.2.4 Exploratory safety outcomes

- New-onset clinical diagnosis of acute coronary syndrome or ST elevation or non-ST elevation myocardial infarction
- New-onset clinical diagnosis of mesenteric ischemia
- New-onset clinical diagnosis of extremity, nose, or ear ischemia
- Clinical diagnosis of vasopressor extravasation
- Clinical diagnosis of clinically-significant arrhythmia (sustained ventricular tachycardia, reentrant [supraventricular] tachycardia, atrial arrhythmia with rapid ventricular response requiring intervention, or new-onset atrial fibrillation or flutter)
- Clinical diagnosis of cardiogenic shock
- Cardiac arrest
- New-onset severe hyponatremia (serum sodium <120 mEq/L) after enrollment
- Maximum lactate from enrollment through day 7

- Serum troponin above upper limit of normal from enrollment through day 7

7.2.5 Monitored care processes

Delivery of interventions for septic shock directly related to the studied intervention or which may be indirectly influenced by the intervention will be evaluated. These include:

- Study-specific threshold-based vasopressin order entered by clinical team at any time
- Receipt of vasopressin at any time after study entry
- Maximum dose of non-vasopressin vasopressors (in norepinephrine equivalents) before initiation of vasopressin. (For patients never initiated on vasopressin, value is the patient's maximum total dose of vasopressors.)
- Receipt of stress-dose steroids from enrollment through study day 7
- Volume of IV resuscitation fluid administered during the first 24 hours after enrollment
- Volume of IV resuscitation fluid administered during the first 72 hours after enrollment
- Placement of new central venous access (central venous catheter or peripherally-inserted central catheter) through study day 7

7.3 Data analysis

Patients will enter the study at the time they first meet all entry criteria at a study hospital. As described in Section 5.2.7, patients' study entry and treatment assignment for intention-to-treat analyses will be based on the date and time they meet all entry criteria at a study hospital, with all subsequent care and outcomes through the end of the index hospital encounter and study follow-up (including any subsequent episodes of shock meeting study entry criteria) contributing to the subjects' study outcomes. For an individual patient, to ensure independence of observations, any episodes of shock meeting study inclusion criteria that occur during distinct hospital encounters subsequent to the index hospital encounter will be included in safety monitoring reports for the Data and Safety Monitoring Board (DSMB, see Sections 7.6 and 10) but will be excluded from other analyses.

Full details of planned analyses will be prespecified in a Statistical Analysis Plan.

7.3.1 Descriptive and adjusted analysis

Categorical variables will be reported as number (percent). For descriptive reporting, continuous variables will be reported as mean (standard deviation) or median (25th-75th percentile). Between-group comparisons will, as appropriate, employ the t-test with unequal variance or the Wilcoxon rank-sum test for continuous variables and chi-squared or Fisher's exact test for categorical testing.

7.3.2 Primary analysis

The primary analysis will compare the effect of the two treatment strategies on 28-day mortality on an intention-to-treat basis using a generalized linear mixed effects model incorporating a logit link, an indicator variable for treatment strategy assignment, a random effect for study hospital (to account for within-hospital correlation), and patient-level adjustment covariates (including age, sex, source of infection suspected or diagnosed at the time of study entry [pulmonary, urinary, GI/abdominal, skin, other/multiple], preexisting kidney disease [see Appendix D], preexisting heart disease [see Appendix D], non-cardiovascular SOFA score, and initiation of vasopressors within 72 hours of hospital arrival). The generalized linear model will use a Wald test to evaluate whether we have evidence of a non-zero effect of the exposure. A two-sided p value <0.05 will be considered significant.

7.3.3 Key secondary analysis

The sole pre-specified secondary analysis will compare the effect of the two treatment strategies on renal replacement therapy-free days on an intention-to-treat basis using analysis and statistical testing methods parallel to that employed for the primary outcome, using a random effects proportional odds model with random effects for study hospital and fixed effects for covariates to evaluate the ordinal outcome.

7.3.4 Exploratory analyses

Exploratory analyses will not include adjustment for multiple comparisons and will thus be considered hypothesis generating. Pre-planned secondary analyses will:

- Evaluate the intervention's effect on exploratory outcomes using a similar statistical model as the primary analysis, including a random effect for study hospital and adjustment for patient-level covariates. Secondary analyses will replace the logit link as needed based on nature of the outcome variable.
- Using a similar statistical model as the primary analysis, including adjustment for patient-level covariates, measure heterogeneity of treatment effect for the primary outcome associated with patient-level covariates included in the adjustment model by incorporating an interaction term between covariate of interest and the treatment assignment and testing the significance of the interaction term's coefficient. Planned covariates for heterogeneity of treatment effect evaluation include: age, sex, source of infection diagnosed at the time of study entry, chronic kidney disease [see Appendix D], preexisting heart disease [see Appendix D], non-cardiovascular SOFA score, and time between hospital presentation and initial receipt of vasopressor (<72 hours vs >= 72 hours).

7.3.5 Sensitivity analyses

Pre-planned sensitivity analyses will include:

- Repeat the primary and key secondary analysis restricted to enrolled patients for whom study-specific threshold-based vasopressin order was entered;
- Repeating the primary analysis without adjustment for baseline patient covariates;
- Repeating the primary analysis adding time as a covariate. The time variable will be a continuous variable indicating study month (values from 1 to 28) and modeled using restricted cubic splines to allow for non-linearity.

7.4 Missing data

Data for the primary outcome and treatment assignment is expected to be non-missing for all patients. Missing outcome and treatment assignment data will not be imputed for any analysis. The prespecified outcome analysis model covariates are also expected to be 100% non-missing after electronic data collection supplemented by manual chart review. However, if missing data for covariates is present for >0.2% of eligible subject, we will perform the primary, secondary, and exploratory outcome analyses after imputing missing values using chained equations. An analysis based on complete cases will be performed as a sensitivity analysis for the primary and key secondary analysis. If covariate missingness is present for ≥1 eligible patient but ≤0.2% of the overall cohort, the primary analysis will be restricted to complete cases, with sensitivity analyses based on multiple imputation by chained equations performed for the primary and key secondary analysis. For descriptive data, missing data will not be imputed and complete case analysis will be used and missingness will be reported.

7.5 Sample size/power analysis

Based on historical data, the study hospitals average roughly 11.7 patients per month (range 1-45) with septic shock of whom 8.25 patients per month (range 0.3-35) receive ≥0.1 mcg/kg/min norepinephrine (or equivalent). We conservatively estimated that the study will last 16 months — including 8-12 months of enrollment at all study sites and an additional 4 month vanguard phase at the largest-volume site (see study timeline depicted in Appendix B) — and will enroll approximately 2050 patients, including approximately 1445 patients with maximum vasopressors dose ≥0.1 mcg/kg/min norepinephrine (or equivalent). Power analyses employed the following additional assumptions:

- Historical patient volumes for each cluster;
- 28-day mortality of 40% among patients receiving ≥0.1 mcg/kg/min norepinephrine (or equivalent) assigned to the higher vasopressin initiation treatment strategy (considered the control group);
- 28-day mortality of 21% for all patients (regardless of treatment assignment) receiving <0.1 mcg/kg/min norepinephrine (or equivalent);
- Within-cluster correlation of 0.003;
- Randomization stratified by hospital patient volume (see Section 4.4 and Appendix B);
- No differential impact of the intervention across clusters; and
- A type 1 error rate of 0.05.

Simulation analyses based on these assumptions estimate that the trial will have 80% power to detect a 5.4% difference in all-cause 28-day mortality (27.6% vs 33%) for the compared treatment groups (Figure 4).[59,60] The final sample size will be determined by the number of patients enrolled with septic shock during the planned trial duration, with a maximum expected enrollment of ≤7000 patients.

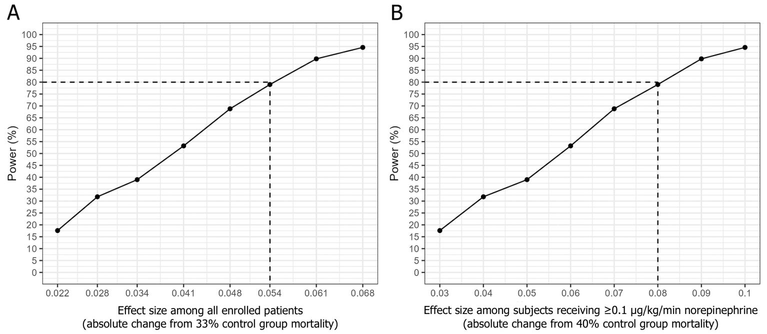

Figure 4. Power estimates for varying effect sizes applied to (A) the overall population and (B) the subset of patients receiving ≥0.1 mcg/kg/min of norepinephrine (or equivalent). Power analyses employed the assumptions described in Section 7.5, 13 clusters, and a 16-month study with monthly crossover as shown in Appendix B.[59,60]

7.6 Interim analysis

The trial DSMB will conduct interim analyses for safety approximately every six months, beginning with availability of data for patients discharged within 6 months of study initiation. In addition to monitoring safety on an ongoing basis, the DSMB will conduct a single interim analysis for efficacy including patients enrolled within the first 8 months of the trial. This analysis will employ a conservative Haybittle-Peto stopping boundary (two-sided p-value <0.001) to test for between-group differences in the primary outcome, allowing the final analysis to proceed without altering the p-value threshold for assessing statistical significance (p=0.05).[61-63] There will be no interim futility analyses, and therefore no formal interim stopping rules for futility. The decision not to include a futility analysis is based on our goal to obtain the most accurate possible effect sizes for this minimal risk study and potential for utilization rate

of study orders to influence study monthly enrollment rates in unpredictable ways. Given that both thresholds are widely practiced, ethically there is not a duty to stop the trial early based on futility.

Prior to the end of the trial, the DSMB will review non-comparative (pooled) data on (1) monthly enrollment; (2) primary outcome incidence; (3) the proportion of enrolled patients who received ≥0.1 mcg/kg/min norepinephrine (or equivalent); and (4) rates of treatment strategy crossover and non-adherence. Lower-than-expected rates of patient enrollment, the primary outcome, or shock treated with ≥0.1 mcg/kg/min norepinephrine (or equivalent) or higher-than-expected treatment strategy crossover and non-adherence could all decrease the study's power to detect the preplanned effect size. Therefore, if meaningful differences from expected values for these factors are observed and continuing the study beyond the planned duration is determined to be operationally feasible,[50-52] the DSMB may request the unblinded biostatistician re-estimate the sample size required to maintain the prespecified power to detect the prespecified treatment effect. Based on this analysis, the DSMB may recommend extending the trial beyond the planned duration and/or resuming the trial after study hospitals' EMR transition up to a maximum of 30 months of active enrollment and a maximum total trial duration of 4 years.[64]

To protect patient safety, the DSMB will have the ability to stop the trial at any time (including before or in between planned safety analyses), to require supplementary data or interim analyses, or to request study protocol revision. DSMB recommendations, including those associated with interim safety and efficacy analyses, will be at the discretion of the DSMB, and may take into account existing and newly-available evidence external to the present study as well as the totality of available data from the present study, including consideration of the p-values from interim safety and efficacy analyses along with other clinical outcomes, adverse events, and other potential clinical factors.

8. RISK ASSESSMENT

This trial is a pragmatic comparison of the effectiveness of two strategies for management of septic shock using vasopressin within the range of current usual practice and guidelines. Patients will all be closely monitored in an ED or ICU setting. This will allow for prompt treatment of any untoward events. It is further anticipated that enhanced safety monitoring using EMR queries and chart review will in fact provide even greater safety monitoring than the already high level applied in routine clinical use. Clinicians will retain and exercise their clinical judgment in the care of patients; the intervention is a default setting that applies usual clinical care in a non-biased way. The trial will be overseen by an independent Data and Safety Monitoring Board (DSMB), which will operate according to an approved charter.

8.1 Potential risks

8.1.1. Risk of vasopressin treatment for septic shock

Vasopressin, FDA-approved and marketed as both generic product and as branded VASOSTRICT™ with a labeled indication for treatment of septic shock, has been used extensively for years in clinical practice. Vasopressin, at the infusion rates and indications studied within this trial, is in common clinical use across study hospitals. The only contraindication to vasopressin administration is allergy to vasopressin or its vehicle. At doses substantially higher than will be used in the present septic shock treatment strategies, vasopressin has been associated with bradycardia and decreases in cardiac output. Based on passive reporting (as specified in the package insert) and general observations in the literature, “the most common adverse reactions include decreased cardiac output, bradycardia, tachyarrhythmias, hyponatremia and ischemia (coronary, mesenteric, skin, digital).” It is anticipated that low, fixed-rate infusions—the clinical standard at study hospitals—are well tolerated without adverse reactions; in common clinical use, no special precautions or monitoring are used for vasopressin as opposed to other vasopressors. The trial in no way proposes or implements a change in the infusion rates of vasopressin; the locus of randomization is *when* the vasopressin infusion should be initiated.

8.1.2. Risk of lower versus higher thresholds for vasopressin initiation

Comparison of the risks and benefits of lower versus higher thresholds is the purpose of the present trial. Because both lower and higher thresholds are routinely encountered in clinical care, with variation in use driven by factors unrelated to patient attributes, the theoretical risks and benefits of lower versus higher thresholds for vasopressin initiation are already features of usual care in the clinical environment.

8.1.3 Risk of confidentiality breach

Potential consequences of a breach of confidentiality to the subject could include identity theft, loss of privacy, theft, embarrassment, or harassment. This risk is extremely low and will be managed intensively by study personnel.

8.2 Alternatives to participation

While randomization is at the level of ICU rather than patient, treating clinicians retain the ability to not prescribe or administer vasopressin at all or to prescribe and administer vasopressin at thresholds other than those evaluated in this trial.

8.3 Minimization of risks

Federal regulations 45 CFR 46.111(a)(1) require that risks to subjects are minimized by using procedures which are consistent with sound research design. This trial meets those requirements, incorporating numerous mechanisms to minimize risks to patients. The trial protocol includes manual and electronic query-based safety monitoring, solicited reporting of potential protocol-related adverse events, and routine monitoring by an independent DSMB empowered to require stopping the trial or modification of the trial protocol at any time. Data privacy and confidentiality will be intensively managed in accordance with Intermountain Health's high-reliability methods and processes.

8.3.1 Protections against altered clinical risk/benefit from vasopressin strategies

Both tested strategies are within the spectrum of care provided to thousands of patients per year at study hospitals (see Figure 1) and to hundreds of thousands of patients nationwide each year.[43] Conditional on the clinician's decision that a patient should receive vasopressin if their response to first-line vasopressors is inadequate, the study essentially aims to compare the effects of implementing two alternative guidelines providing (compared to current guidelines) more definitive recommendations regarding the threshold for adding vasopressin to first-line vasopressors for septic shock. As such, the pragmatic intervention explicitly encourages clinicians to use their clinical judgment for individual patients to including assessment of whether patient factors require (1) choosing an alternative threshold for starting vasopressin or (2) not prescribing vasopressin. Clinician education delivered prior to study launch and refreshed at intervals during study conduct will address this topic and include discussion of conditions that have potential to alter risk/benefit for vasopressin based on prior evidence or pathophysiologic consideration. In addition, in the presence of patient factors that may alter the risk/benefit profile of vasopressin, EMR-based logic will suppress delivery of prompts to clinicians to consider use of study-specific threshold-based vasopressin orders (see Section 5.2.2.3 and Appendix C).

8.3.2 Protections in setting of potential allergy to vasopressin or its vehicle

Vasopressin is an endogenous hormone, and allergy or adverse event history to the drug or its vehicle is expected to be rare. We were unable to identify any reports of vasopressin allergy in the medical literature as of November 2022. Study hospitals employ a shared electronic medical record with embedded allergy/adverse event checking and decision support during medication electronic order entry. These mechanisms will notify ordering clinicians of potential vasopressin allergy/adverse event at the time of order entry and prevent completion of order for vasopressin unless — as per usual practice — clinician specifically and intentionally overrides based on clinical judgement. We anticipate that these robust mechanisms will prevent administration of vasopressin to patients in whom an allergy is present.

8.3.3 Protections against loss of privacy or breach of confidentiality

Protection of participant identity and prevention of unintentional release of protected information is of paramount importance to the study team.

- *Data storage and transfer*: Data will be maintained in password-protected, encrypted research servers, REDCap (a secure, HIPAA-compliant system), or password-protected, encrypted computers. Intermountain Health deploys comprehensive information technology support systems to maintain and regularly update computer systems and maintain physical and electronic safeguards for data management. Any paper case report forms will include minimal identifiers and will be stored in secure locations.
- *Data access*: Only the PI, co-investigators, and the study team will have access to identifying information. All individuals with access to identifiable data will have all necessary human subjects training and, as applicable, Good Clinical Practice training.

- *Training*: All study personnel will maintain appropriate training in human subjects research and data protection. The PI, co-investigators, and study staff responsible for study coordination, data collection, and data management of the associated clinical trial will maintain Good Clinical Practice training.
- *Certificate of Confidentiality*: A Certificate of Confidentiality as described in subsection 301(d) of the Public Health Service Act (42 U.S.C 24) will be obtained from the National Institutes of Health. The Certificate of Confidentiality will protect the privacy of research participants by prohibiting disclosure of identifiable information compiled for purposes of the trial (e.g., in response to legal subpoena) without consent of the individual identified except as required by Federal, State, or local laws or for the purposes of other scientific research that is in compliance with applicable Federal regulations governing the protection of human subjects in research.

8.3.4 Incidental findings
Data review and analysis will use only data collected as part of routine clinical care and operations. We therefore do not expect identification of new incidental findings relevant to patient care.

8.4 Potential benefits of the proposed research to human subjects and others
Study subjects may or may not receive any direct benefits from their participation in this study. It is unclear whether a lower (versus higher) threshold for initiating vasopressin as a secondary vasopressor in septic shock will provide benefit. Thus, the optimal treatment is undefined at this point. This study has the potential to generate important findings for patients with sepsis and their treating clinicians, including optimal strategies for vasopressor management in septic shock. The risks to patient confidentiality and the risks for adverse drug reactions appear substantially less than the potential benefit to sepsis patients and the important knowledge gained about delivery of septic shock care.

8.5 Importance of the knowledge to be gained
Patients with sepsis —a syndrome defined by infection associated with acute organ dysfunction — have high mortality. Septic shock is a common reason for ICU admission with even higher mortality. Optimal strategies for management of patients who receive more than low doses of vasopressors to achieve target blood pressure are unclear. The overall hypothesis of this pragmatic comparative effectiveness trial is that a lower (versus higher) threshold for starting vasopressin as a secondary vasopressor is associated with improved clinical outcomes. Based on the preceding assessment of risks and potential benefits, the risks to subjects (no more than minimal risk) are acceptable in relation to anticipated knowledge to be gained to improve care for the common and deadly sepsis syndrome.

9. HUMAN SUBJECTS

We will prospectively assess the effect of septic shock treatment strategies encouraging a lower versus higher threshold for initiating vasopressin as a secondary vasopressor. In this pragmatic, embedded, comparative effectiveness trial, strategies will be implemented at the hospital level, and patients with septic shock will be exposed to the treatment strategy in place at the study hospital at the time they initiate vasopressors, with patients' treating clinicians exercising clinical judgment as to whether to apply a study strategy to any individual patient.

9.1 Selection of subjects

Federal regulations at 45 CFR 46(a)(3) require the equitable selection of subjects. All eligible patients will be enrolled. Study exclusion criteria neither unjustly exclude classes of individuals from participation in the research nor unjustly include classes of individuals from participation in the research. Hence, the recruitment of subjects conforms to the principle of distributive justice.

9.2 Waiver of informed consent and HIPAA authorization

Recognizing feasibility, generalizability, methodological integrity, and appropriate ethical precedents, we will request a waiver of informed consent and HIPAA authorization based on the following criteria as per 45 CFR 46.116(f) and the HIPAA Privacy Rule. Numerous previous randomized trials comparing two or more strategies or treatments within standard of care for critically ill and other hospitalized patients have been completed under a waiver of informed consent.[53,54,65-84]

9.2.1 Research involves no more than minimal risk

Vasopressin is recommended as a preferred second-line vasopressor in septic shock, but the threshold for adding this agent to first-line vasopressors varies widely between patients and — demonstrating practice variation not driven by patient factors — across hospitals. The planned trial will compare two strategies for vasopressin initiation that fall within the range of practice commonly used in routine clinical care. Both are strategies to which patients could be exposed even if not participating in the study. No established differences in risk and benefit are known to exist between the two approaches based on the currently available data. Clinicians will be free to deviate from the assigned strategy based on clinical judgement. If a clinician feels an individual patient will benefit from starting vasopressin at a lower threshold, the clinician will be permitted to start vasopressin at a lower threshold regardless of group assignment. If a clinician feels that an individual patient will benefit from starting vasopressin at a higher threshold, the clinician will be permitted to start vasopressin at a higher threshold regardless of group assignment. Thus, the only patients for whom the study-defined treatment strategy will determine the threshold at which vasopressin is started are those patients for whom treating clinicians feel that either of the study's two vasopressin initiation thresholds is consistent with safe and effective care for that patient. The approach taken by the VASSPR trial — replacing the arbitrary exposure to competing, unproven therapies seen currently with vasopressin with structured variation created by randomization[85-87] — is a well-accepted strategy recently adopted in numerous other "embedded pragmatic clinical effectiveness trials" to answer important clinical questions in critical care medicine[53,54,73-80] specifically and both inpatient[81-83] and outpatient[84,88-91] medicine generally.

9.2.2 No adverse effects on the rights and welfare of participants

Exposure to (1) structured variation within the range of accepted and observed usual care — rather than arbitrary variation — with regard to initiation of secondary vasopressors and (2) research team abstraction and analysis of data routinely collected in the course of clinical care should have no adverse

effects on the rights or welfare of study subjects. Because risk is minimal given this design, the waiver of consent and authorization will not adversely affect the rights or welfare of subjects.

9.2.3 The research could not practicably be carried out without waiver of informed consent

Conceptually, this trial compares outcomes associated with adoption of two different guidelines for secondary vasopressor initiation in septic shock. Delivery of the two different treatment strategies will therefore be integrated within usual care via the kinds of implementation methods used in real-world practice to standardize clinical care and aid adoption of new guidelines. These implementation methods target groups of physicians, nurses, and pharmacists at the unit/hospital level rather than targeting at the level of individual patients. The trial’s underlying question and intervention deployment strategies therefore require use of an embedded, cluster-randomized trial design rather than individual patient randomization. As a result, while clinicians are empowered to individualize vasopressor management for each patient as needed, all patients treated with vasopressors for septic shock at a study hospital will be exposed to the vasopressin treatment strategy assigned for that month at that hospital. It would be impracticable and unethical, violating core principles of informed consent, to require patients to choose between giving informed consent for trial participation or transferring to another hospital before starting treatment for septic shock, especially since vasopressor initiation for critically ill adults is a time-sensitive procedure for which delay increases the likelihood of life-threatening hemodynamic compromise. Restricting study enrollment and analysis to strategy-exposed patients capable of providing consent — a non-representative subset of all strategy-exposed patients due to the condition’s high mortality rate and frequent association with impaired mental status/decisional capacity — would compromise the scientific validity of the study. In summary, because of these considerations, this research could not practicably be carried out without the waiver of consent.

9.2.4 Information on the trial will be available to all ICU patients

We will make available patient information sheets describing the trial in lay language via distribution in study ICUs to all ICU patients. Information sheets may be posted or distributed in public areas of the ICU (e.g. waiting room), core entryways, and/or included in information packets provided to patients and their families. Posters with general information on the study may also be displayed in patient rooms and/or public areas of the ED and ICU. Providing information specifically to patients enrolled in the study (such as the time of meeting entry criteria) is not feasible given the pragmatic, distributed nature of the intervention across 13 hospitals, likelihood that many patients will have passed away or not be at a home address at time of retrospective query-based eligibility identification, and risk that attempting to contact patients would substantially increase the risk of a confidentiality breach.

9.2.5 The minimal necessary amount of protected information will be obtained

Only data necessary for the completion of the study will be collected. The patient ID, encounter ID, social security number, address, and date of birth are necessary to allow data linkage and identification of the specific patient encounter for supplemental data abstraction. Event date/times are necessary for care process and outcome measurement, including calculation of the elapsed time related to various patient management actions.

9.2.6 All data, including PHI, will be securely guarded from improper disclosure

High-level safeguards will be in place to protect subject identity and confidential data. The study data will be kept on encrypted, password-protected computers. These computers are routinely used for storage of patient data and research data including subject identifiers. The data will only be accessible to members of the research team, and all members of the research team have completed Human Subjects

Protections and understand the importance of protecting subject privacy and confidentiality. Protected health information will not be reused or disclosed to any other person or entity, except as required by law, for authorized oversight of the research project, or for other research for which the use or disclosure of protected health information would be permitted by under applicable regulations. Minimal identifiers will be maintained linked to the data. Maintaining minimal identifiers linked to the data is necessary to allow (1) manual abstraction of additional data from the electronic medical record and (2) potential future linkage – with IRB approval – of the data to additional datasets. No individual subject data will be presented in any presentation, publication or report related to this research. Data will be presented only in anonymized or aggregate form or as results of statistical analyses and will not include any individual-level data that could be traced to a particular subject.

10. SAFETY AND DATA MONITORING AND REPORTING

10.1 Summary and rationale for safety and data monitoring and reporting

The study Pis and steering committee will be responsible for data and safety monitoring associated with achieving the study objectives. The VASSPR trial will be overseen by an independent Data and Safety Monitoring Board (DSMB). The DSMB —comprised of independent individuals with expertise in critical care medicine, sepsis, biostatistics, clinical trial design and conduct, and clinical trial ethics — will operate according to an approved charter.

Assuring patient safety is an essential component of this clinical trial. The approach to data and safety monitoring and assessment within this trial reflects attributes of the studied treatment strategies and the target population and the environment in which study subjects will be receiving care.[92]

- Vasopressin is a mature, FDA-approved, and widely-marketed therapy for septic shock with which there is extensive experience. Treatment of septic shock with vasopressin as a second-line vasopressor generally and the tested strategies involving alternative threshold for vasopressin initiation specifically are in common clinical use both across the United States and at the study hospitals. Vasopressin has a labelled indication for septic shock and its use in the present study is consistent with the range of practice observed in the U.S. and at study hospitals. It is anticipated that low-dose, fixed-rate infusions—the clinical standard at study hospitals—are well tolerated without adverse reactions; in common clinical use, no special precautions or monitoring are used for vasopressin as opposed to other vasopressors.
- The study intervention involves randomization to one of two “default strategies” for initiation of secondary vasopressors in septic shock. If treating clinicians believe an alternative approach to shock management is preferable for an individual patient at any point in their care, clinicians will be able to select the management approach that the treating clinicians judge to be best.
- Patients with septic shock are critically ill and may be reasonably anticipated to experience multiple adverse events (AEs) regardless of any study procedures.
- Clinical care for study subjects will occur in clinical settings dedicated to provision of highest-acuity clinical care and within the context of routine clinical care for a high-acuity procedure (vasopressor administration) employed to treat septic shock. Characteristics of routine care while on vasopressor therapy include:
    - Care by a critical care, emergency medicine, or other appropriately trained nurse with a low patient-to-nurse staffing ratio.
    - ED patients have immediate access to an ED provider, while ICU patients will have immediate access to an in-house physician as well as either direct access to an in-house critical care physician or telemedicine-based access to a critical care physician.
    - Routine clinical care for septic shock patients will — independent of any study procedures — involve continuous or frequent monitoring of blood pressure and other vital signs via invasive or non-invasive means as well as frequent monitoring of physical exam, symptoms, and laboratory parameters.

Given these considerations, trial safety monitoring will combine careful monitoring via chart review and electronic queries with a focus on potential adverse effects identified from vasopressin product labeling and prior clinical trials and active surveillance via event reporting by bedside clinical personnel. Also consistent with these considerations, investigator AE review will not take place except for AEs which clinical personnel flag as potentially related to study procedures, and narrative-based AE reporting will not take place unless review by the research team finds event is both possibly, probably or definitely

related to study procedures and either (1) serious or (2) suggests the research places subjects or others at a greater risk of harm than was previously known or recognized.

10.2 Potential Risks and Benefits for Participants

- Potential risks: The risk level associated with this study is estimated to be no more than minimal, since the studied septic shock management strategies are in common clinical use across study hospitals prior to the study. The pragmatic intervention will replace arbitrary variation with structured variation by mimicking implementation of a care guideline or recommendation for vasopressin initiation timing with a clearer threshold for starting vasopressin than is currently available while preserving clinicians' ability to apply clinical judgement and select treatment approaches outside the suggested strategies for individual patients as deemed necessary. See Section 8.3.1 for additional details.
- Potential benefits: Study subjects may or may not receive any direct benefits from their participation in this study. This study has the potential to generate important findings for patients with sepsis and their treating clinicians, including optimal strategies for vasopressor management in septic shock. See Section 8.4 for additional details.

10.3 Data and Safety Monitoring Board (DSMB)

The principal role of the DSMB is to assure the safety of patients in the clinical trial that will test septic shock strategies involving two default thresholds for vasopressin initiation.

10.3.1 DSMB membership

The DSMB will consist of members independent of the clinical trial with expertise in critical care, sepsis, biostatistics, clinical trial design and conduct, and clinical trial ethics.

10.3.2 DSMB responsibilities

The DSMB will make recommendations to the principal investigator (PI) and trial steering committee with respect to:

- Review and approve the research protocol and plans for data and safety monitoring;
- Protect the safety of the study participants, including review of safety outcomes and adverse events during routinely scheduled meetings and at other intervals as needed;
- Evaluate the progress of the trial, including periodic assessments of data quality and timeliness, recruitment, accrual, participant risk versus benefit, and other factors that can affect study outcome;
- Consider factors external to the study when relevant information becomes available, such as scientific or therapeutic developments that may have an impact on the safety of the participants or the ethics of the trial;
- Review study performance, make recommendations and assist in the resolution of problems reported by the principal investigator (PI) or their delegate;
- Make recommendations to the PI and trial steering committee concerning continuation, termination or other modifications of the trial;
- Ensure the confidentiality of the study data and the results of monitoring; and,
- Assist the IRB by commenting on any concerns related to study conduct, enrollment, sample size, and/or data collection.

The PI will be responsible for the preparation of all DSMB and adverse event reports. DSMB recommendations — including recommendations to end, modify, or continue the trial — will be

communicated to the PI in writing (paper or electronic). The PI will be responsible for sharing these recommendations with the IRB.

10.3.3 DSMB meeting frequency

The DSMB will meet prior to initiation of the clinical trial to review the trial protocol and plan for monitoring study progress and safety measures. Approval by the DSMB and Institutional Review Board (IRB) will be required prior to initiation of the clinical trial. After trial initiation, the DSMB will meet approximately every six months to review study progress and safety data. The DSMB may convene additional *ad hoc* meetings as needed upon determination of the DSMB chair, either alone or in consultation with the trial PI and/or trial steering committee. An interim efficacy analysis will be conducted after primary outcome data are available for the primary outcome for patients enrolled through the end of study month 14. There will be no futility analysis.

10.3.4 Conflict of interest for DSMB

The members of the DSMB will have no direct involvement with the study investigators or intervention related to the trial outside of DSMB meetings and other DSMB-related communications. DSMB members will provide a Conflicts of Interest Disclosure which includes current affiliations, if any, with pharmaceutical and biotechnology companies (e.g., stockholder, consultant), and any other relationship that could be perceived as a conflict of interest related to the study and/or associated with commercial interests pertinent to study objectives.

10.4 Adverse Event Definitions

- **Adverse event** — A clinical trial adverse event is any untoward medical event temporally associated with the research participation and which is not tracked as a clinical outcome, whether or not it is considered related to a drug or study procedure.

- **Seriousness:** A ***serious adverse event (SAE)*** is any AE that results in any one or more of the following outcomes:

    - Death
    - A life-threatening experience (that is, immediate risk of dying)
    - Event requiring inpatient hospitalization or prolongation of existing hospitalization.
    - Persistent or significant disability or incapacity.
    - Congenital anomaly or birth defect
    - Important medical events that may not result in death, be life-threatening, or require hospitalization may be considered serious adverse events when, based upon appropriate medical judgment, they may jeopardize the patient and may require medical or surgical intervention to prevent one of the outcomes listed in this definition.

- **Relatedness:** The study uses the following AE attribution scale:
    - <u>Definitely Related</u>: The event follows: a) A reasonable, temporal sequence from a study procedure; and b) Cannot be explained by the known characteristics of the patient’s clinical state or other therapies; and c) Evaluation of the patient’s clinical state indicates to the investigator that the experience is definitely related to study procedures.

    - Probably or Possibly Related: The event should be assessed following the same criteria for "Definitely Associated". If in the investigator's opinion at least one or more of the criteria are not present, then "probably" or "possibly" associated should be selected.
    - Probably Not Related: The event occurred while the patient was on the study but can reasonably be explained by the known characteristics of the patient's clinical state or other therapies.
    - Definitely Not Related: The event is definitely produced by the patient's clinical state or by other modes of therapy administered to the patient.
    - Uncertain Relationship: The event does not meet any of the criteria previously outlined.

- **Unanticipated adverse events:** An adverse event that, in the judgment of the study principal investigator or their delegate, is out of the range of that typically observed in terms of nature, severity, or frequency for the patient's underlying critical illness, septic shock, and baseline medical conditions/health.

  Unanticipated adverse events that are also serious and are judged to be possibly, probably, or definitely related to study procedures will be classified as a ***serious unanticipated suspected adverse reaction (SUSAR)***.

- **Unanticipated problem (UP):** Any incident, experience, or outcome that in the judgment of the study principal investigator or their delegate meets all of the following criteria:
    - Unanticipated (in terms of nature, severity, or frequency) given (a) the research procedures; and (b) the characteristics of the subject population being studied, including the anticipated course of subjects' critical illness and septic shock and their associated conditions and predisposing risk factors;
    - Related or possibly related to participation in the research (in this guidance document, *possibly related* means there is a reasonable possibility that the incident, experience, or outcome may have been caused by the procedures involved in the research); and
    - Suggests that the research places subjects or others at a greater risk of harm (including physical, psychological, economic, or social harm) than was previously known or recognized.

10.5 Adverse Event Monitoring and Reporting

Patients eligible for this study are critically ill and at high risk of death, serious organ dysfunction requiring intervention, prolonged hospitalization, or other adverse outcomes. Study personnel have defined a system to collect, review, and closely monitor clinical outcomes and adverse events appropriate to patients' baseline risk, the nature of the study intervention, the pragmatic trial design, the study's planned methods for data collection, and the environment in which patients will receive care (Figure 5). Safety outcomes and potentially reportable adverse events will be identified via a combination of electronic queries of the medical record, structured manual review of the electronic medical record by trained study personnel, and collection of reports of suspected adverse reactions from bedside clinical personnel. Consistent with established procedures in both traditional and pragmatic trials enrolling critically ill patients, investigator review and adjudication will focus on events flagged by research or clinical personnel as potentially related to study procedures.[93]

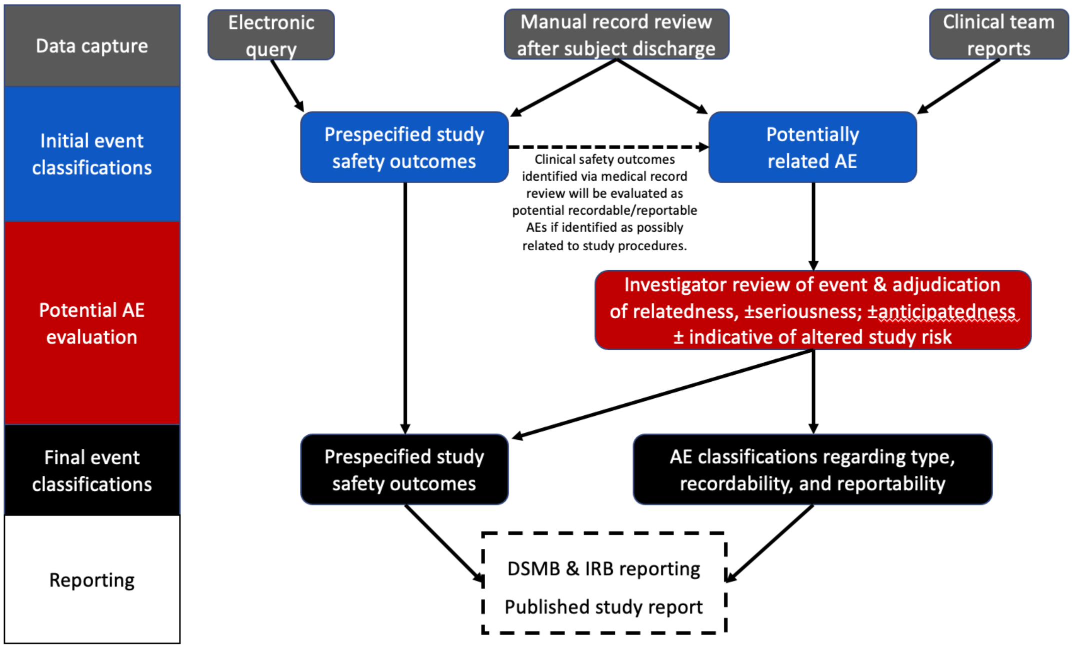

Figure 5. AE monitoring schema for VASSPR trial.

10.5.1 Prespecified study safety outcomes

A prespecified list of clinical outcomes will be systematically collected via electronic queries and manual review of the electronic medical record (see Section 7.2) for all study subjects. Data on study safety outcomes as well as hospital mortality will be reviewed for enrolled subjects at scheduled meetings of the study DSMB unless events meet reportable adverse event criteria described below.

10.5.2 Anticipated clinical events (not considered adverse events)

Anticipated clinical events are adverse events consistent with subjects' septic shock and underlying critical illness and preexisting or concurrent medical conditions. Anticipated clinical events will be collected in the study data and included in study clinical outcomes as appropriate. Anticipated clinical events will not be considered reportable adverse events unless the event is considered by the study team to be possibly, probably, or definitely related to study procedures.

The following is a partial list of adverse events that, through end of study follow up, will be considered anticipated clinical events that will be captured as clinical outcomes:

- Death (all deaths will be captured as part of routine data collection)
- Progressive, persistent, or recurrent shock
- New, secondary, or healthcare associated infections
- New, persistent, or progressive organ dysfunction or failure (and any associated symptoms, signs, and interventions) including but not limited to:
    - Cardiac failure (including heart failure, cardiac arrest, progressive or recurrent shock with or without receipt of vasopressors)
    - Cardiac arrhythmias
    - Myocardial injury or infarction
    - Liver injury, dysfunction, or failure

- Acute kidney injury or failure, including laboratory abnormalities or treatment with renal replacement therapy
  - Hematologic abnormalities (e.g. thrombocytopenia, coagulopathy, bleeding or hemorrhage, clotting [including arterial and venous thromboembolism])
  - Gastrointestinal system symptoms or dysfunction (e.g. vomiting, ileus, bowel obstruction, constipation, bleeding)
  - Respiratory symptoms, dysfunction, or failure, including use of supplemental oxygen or non-invasive or invasive respiratory support
  - Neurologic dysfunction or injury (e.g. delirium, disability, physical or cognitive impairment, ischemic or hemorrhagic stroke, and seizure)
  - Endocrine dysfunction
- New or progressive global weakness, fatigue, or physical impairment (e.g. critical illness myopathy, inability to perform previous level of cognitive or physical function or self care)
- New or progressive focal or generalized pain
- New or exacerbated psychiatric symptoms or diagnoses, including depression, anxiety, and PTSD
- Skin breakdown, rash, pressure ulcers.
- Co-interventions for treatment and monitoring (e.g. ICU antibiotic therapy, blood transfusion, arterial line placement)
- Collection, measurement, abnormalities, or alterations of clinically-collected assessments including:
  - All values for vital signs (e.g. Glasgow Coma Scale score, temperature, respiratory rate, oxygen saturation)
  - Physical exam
  - Patient history/symptoms
  - All laboratory testing and results (e.g. hematocrit, chloride, hepatitis testing)
  - All microbiologic testing and results
  - All diagnostic/radiologic testing and results (e.g. echocardiogram, HIDA scan, chest X-ray, sleep study)
- Duration of treatments and interventions (e.g. vasopressor days, ICU length of stay)
- Prolonged hospitalization or ICU stay
- Occurrence and duration of readmission to ED, hospital and/or ICU and associated diagnoses, care, investigations, and treatments

10.5.3 Reportable adverse events

For purposes of the present trial, a **reportable adverse event** is any event that in the judgment of the study PI or a qualified co-investigator is possibly, probably, or definitely related to study procedures. Adverse events that do not meet this definition will not be separately recorded or reported.

10.5.4 Adverse event reporting

The PI will report (1) SAEs that are possibly, probably, or definitely related to study procedures, (2) UPs, and (3) SUSARs to the DSMB and IRB within 7 calendar days after identification of the event. Narrative reports for these events will be anonymized and will include the nature of the event (including any interventions or treatments), its onset and cessation, outcome, and the principal or delegate investigator's opinion regarding the event's anticipatedness and the relationship between study procedures and the event. AEs not qualifying for narrative reporting will be reported in aggregate form as part of regularly-scheduled semiannual reports to the DSMB and annual reports to the IRB (Figure 6).

	**Unanticipated and related and indicative of altered risk**	**Unanticipated and related but not indicative of altered risk**	**Related but not unanticipated**	**Not related**
**Serious**	SUSAR and UP 7 calendar day narrative reporting	SUSAR 7 calendar day narrative reporting	Related SAE 7 calendar day narrative reporting	Non-reportable AE, safety outcome, or anticipated clinical event
**Not serious**	UP 7 calendar day narrative reporting	Unanticipated & related AE, routine aggregate reporting	Related AE, routine aggregate reporting	

Figure 6. AE event classification and reporting.

AE reports and annual summaries will not include subject- or group-identifiable material. Data will be presented in a blinded manner during open sessions involving the DSMB. At meetings with the DSMB or in IRB reports, data and discussion are confidential. Participant identities will not be known to the DSMB or to the IRB.

10.6 Enrollment, procedure, regulatory, and data quality monitoring

The trial data monitoring plan will include

1. Review of accrued data (including AEs) at monthly meetings of the study protocol committee;
2. Monitoring of data quality and interim safety and data analysis performed by the DSMB;
3. Semi-annual audits of study materials by the regulatory affairs group;
4. An annual review performed by the responsible IRB.

Careful monitoring of the data collection, regulatory adherence, data quality, and study procedures will help to protect the safety of study subjects, the quality of data, and the integrity of the study. The PI or study staff will review all data collection on an ongoing basis for data completeness and accuracy as well as protocol compliance. Data verification for key demographic, exposure, and outcome parameters will be performed on an ongoing basis for a random 5% of all trial subjects. This will occur on the basis of data reabstraction by someone other than the individual who originally collected the data or by blinded duplicate data entry. The results of the ongoing data review will be incorporated into each review with the DSMB. A statement reflecting the results of the ongoing data review will be incorporated into the annual report for the IRB.

10.7 Frequency of data and safety monitoring

The PI and study team will review data quality and adverse event reports monthly and meet with the DSMB approximately every 6 months, either in-person or by teleconference call, to review study progress, data quality, and participant safety data. A summary report on all adverse events and data quality will be submitted to the IRB during annual recertification as per IRB guidelines.

11. AMENDMENT HISTORY

Protocol version and date	Description of change and brief rationale	Sections
Version 1.0<br>(23 January 2024)	N/A	N/A [Original protocol]
Version 1.1<br>(17 May 2024)	Clarify that norepinephrine doses referenced throughout the protocol and employed during clinical care at study hospitals represent norepinephrine base and specify that study hospitals employ the bitartrate formulation of norepinephrine in accordance with 2024 SCCM/ESICM position paper addressing these issues. [18]	Section 3.2<br>Section 4.1<br>Appendix A
	Provide detailed calculation methods for the ICU-free days exploratory clinical outcome.	Appendix D
	Clarify methods for calculation of the hospital-free days exploratory clinical outcome when patients are discharged alive.	Appendix D
	Clarify vasopressor-free and renal replacement therapy-free day definitions by naming the described method for outcome calculation as the "last off" method.	Appendix D
	Correct date of protocol version 1.0 in amendment history table.	Section 11
	Apply consistent terminology for secondary vasopressor strategy evaluation.	Section 2 (background)<br>Section 2 (hypothesis)<br>Section 3.8<br>Section 4.8<br>Sections 8.4-8.5<br>Sections 9<br>Sections 9.2.2-9.2.3<br>Section 10.1
	Format exploratory outcomes to precisely match descriptions registered on ClinicalTrials.gov prior to trial initiation.	Section 2 (exploratory outcomes)<br>Sections 7.2.3-7.2.4

12. BIBLIOGRAPHY

1. Singer M, Deutschman CS, Seymour CW, et al. The Third International Consensus Definitions for Sepsis and Septic Shock (Sepsis-3). *JAMA.* 2016;315(8):801-810.
2. Rhee C, Dantes R, Epstein L, et al. Incidence and Trends of Sepsis in US Hospitals Using Clinical vs Claims Data, 2009-2014. *JAMA.* 2017;318(13):1241-1249.
3. Gaieski DF, Edwards JM, Kallan MJ, Carr BG. Benchmarking the incidence and mortality of severe sepsis in the United States. *Crit Care Med.* 2013;41(5):1167-1174.
4. Kaukonen K-M, Bailey M, Suzuki S, Pilcher D, Bellomo R. Mortality related to severe sepsis and septic shock among critically ill patients in Australia and New Zealand, 2000-2012. *JAMA.* 2014;311(13):1308-1316.
5. Buchman TG, Simpson SQ, Sciarretta KL, et al. Sepsis Among Medicare Beneficiaries: 1. The Burdens of Sepsis, 2012-2018. *Crit Care Med.* 2020;48(3):276-288.
6. Iwashyna TJ, Ely EW, Smith DM, Langa KM. Long-term cognitive impairment and functional disability among survivors of severe sepsis. *JAMA.* 2010;304(16):1787-1794.
7. Prescott HC, Langa KM, Iwashyna TJ. Readmission diagnoses after hospitalization for severe sepsis and other acute medical conditions. *JAMA.* 2015;313(10):1055-1057.
8. Shankar-Hari M, Phillips GS, Levy ML, et al. Developing a New Definition and Assessing New Clinical Criteria for Septic Shock: For the Third International Consensus Definitions for Sepsis and Septic Shock (Sepsis-3). *JAMA.* 2016;315(8):775-787.
9. Kadri SS, Rhee C, Strich JR, et al. Estimating ten-year trends in septic shock incidence and mortality in United States academic medical centers using clinical data. *Chest.* 2017;151(2):278-285.
10. Evans L, Rhodes A, Alhazzani W, et al. Surviving Sepsis Campaign: International Guidelines for Management of Sepsis and Septic Shock 2021. *Crit Care Med.* 2021;49(11):e1063-e1143.
11. Avni T, Lador A, Lev S, Leibovici L, Paul M, Grossman A. Vasopressors for the Treatment of Septic Shock: Systematic Review and Meta-Analysis. *PLoS One.* 2015;10(8):e0129305.
12. Annane D, Vignon P, Renault A, et al. Norepinephrine plus dobutamine versus epinephrine alone for management of septic shock: a randomised trial. *Lancet.* 2007;370(9588):676-684.
13. De Backer D, Biston P, Devriendt J, et al. Comparison of dopamine and norepinephrine in the treatment of shock. *N Engl J Med.* 2010;362(9):779-789.
14. Cheng L, Yan J, Han S, et al. Comparative efficacy of vasoactive medications in patients with septic shock: a network meta-analysis of randomized controlled trials. *Crit Care.* 2019;23(1):168.
15. Stolk RF, van der Poll T, Angus DC, van der Hoeven JG, Pickkers P, Kox M. Potentially Inadvertent Immunomodulation: Norepinephrine Use in Sepsis. *Am J Respir Crit Care Med.* 2016;194(5):550-558.
16. Stolk RF, van der Pasch E, Naumann F, et al. Norepinephrine Dysregulates the Immune Response and Compromises Host Defense during Sepsis. *Am J Respir Crit Care Med.* 2020;202(6):830-842.
17. van der Poll T, Jansen J, Endert E, Sauerwein HP, van Deventer SJ. Noradrenaline inhibits lipopolysaccharide-induced tumor necrosis factor and interleukin 6 production in human whole blood. *Infect Immun.* 1994;62(5):2046-2050.

18. Wieruszewski PM, Leone M, Kaas-Hansen BS, et al. Position Paper on the Reporting of Norepinephrine Formulations in Critical Care from the Society of Critical Care Medicine and European Society of Intensive Care Medicine Joint Task Force. *Crit Care Med.* 2024;52(4):521-530.
19. Chawla LS, Busse L, Brasha-Mitchell E, et al. Intravenous angiotensin II for the treatment of high-output shock (ATHOS trial): a pilot study. *Crit Care.* 2014;18(5):534.
20. Khanna A, English SW, Wang XS, et al. Angiotensin II for the Treatment of Vasodilatory Shock. *N Engl J Med.* 2017;377(5):419-430.
21. Holmes CL, Patel BM, Russell JA, Walley KR. Physiology of vasopressin relevant to management of septic shock. *Chest.* 2001;120(3):989-1002.
22. Landry DW, Levin HR, Gallant EM, et al. Vasopressin deficiency contributes to the vasodilation of septic shock. *Circulation.* 1997;95(5):1122-1125.
23. Reid IA. Role of vasopressin deficiency in the vasodilation of septic shock. *Circulation.* 1997;95(5):1108-1110.
24. Nagendran M, Russell JA, Walley KR, et al. Vasopressin in septic shock: an individual patient data meta-analysis of randomised controlled trials. *Intensive Care Med.* 2019;45(6):844-855.
25. Ukor IF, Walley KR. Vasopressin in Vasodilatory Shock. *Crit Care Clin.* 2019;35(2):247-261.
26. Sacha GL, Bauer SR, Lat I. Vasoactive Agent Use in Septic Shock: Beyond First-Line Recommendations. *Pharmacotherapy.* 2019;39(3):369-381.
27. Dunser MW, Hasibeder WR. Sympathetic overstimulation during critical illness: adverse effects of adrenergic stress. *J Intensive Care Med.* 2009;24(5):293-316.
28. Dunser MW, Mayr AJ, Ulmer H, et al. Arginine vasopressin in advanced vasodilatory shock: a prospective, randomized, controlled study. *Circulation.* 2003;107(18):2313-2319.
29. Luckner G, Mayr VD, Jochberger S, et al. Comparison of two dose regimens of arginine vasopressin in advanced vasodilatory shock. *Crit Care Med.* 2007;35(10):2280-2285.
30. Lauzier F, Levy B, Lamarre P, Lesur O. Vasopressin or norepinephrine in early hyperdynamic septic shock: a randomized clinical trial. *Intensive Care Med.* 2006;32(11):1782-1789.
31. Barzegar E, Nouri M, Mousavi S, Ahmadi A, Mojtahedzadeh M. Vasopressin in Septic Shock; Assessment of Sepsis Biomarkers: A Randomized, Controlled Trial. *Indian J Crit Care Med.* 2017;21(9):578-584.
32. Hajjar LA, Zambolim C, Belletti A, et al. Vasopressin Versus Norepinephrine for the Management of Septic Shock in Cancer Patients: The VANCS II Randomized Clinical Trial. *Crit Care Med.* 2019;47(12):1743-1750.
33. Gordon AC, Mason AJ, Thirunavukkarasu N, et al. Effect of Early Vasopressin vs Norepinephrine on Kidney Failure in Patients With Septic Shock: The VANISH Randomized Clinical Trial. *JAMA.* 2016;316(5):509-518.
34. Russell JA, Walley KR, Singer J, et al. Vasopressin versus norepinephrine infusion in patients with septic shock. *N Engl J Med.* 2008;358(9):877-887.
35. Gradwohl-Matis I, Dunser MW. On sepsis, troponin and vasopressin: the bitter truth. *Crit Care.* 2013;17(5):1002.
36. Parrillo JE. Septic shock--vasopressin, norepinephrine, and urgency. *N Engl J Med.* 2008;358(9):954-956.

37. Heavner MS, McCurdy MT, Mazzeffi MA, Galvagno SM, Jr., Tanaka KA, Chow JH. Angiotensin II and Vasopressin for Vasodilatory Shock: A Critical Appraisal of Catecholamine-Sparing Strategies. *J Intensive Care Med.* 2021;36(6):635-645.
38. Wieruszewski PM, Khanna AK. Vasopressor Choice and Timing in Vasodilatory Shock. *Crit Care.* 2022;26(1):76.
39. Ammar MA, Ammar AA, Wieruszewski PM, et al. Timing of vasoactive agents and corticosteroid initiation in septic shock. *Ann Intensive Care.* 2022;12(1):47.
40. Sacha GL, Lam SW, Wang L, Duggal A, Reddy AJ, Bauer SR. Association of Catecholamine Dose, Lactate, and Shock Duration at Vasopressin Initiation With Mortality in Patients With Septic Shock. *Crit Care Med.* 2021.
41. Gordon AC, Russell JA, Walley KR, et al. The effects of vasopressin on acute kidney injury in septic shock. *Intensive Care Med.* 2010;36(1):83-91.
42. Patel BM, Chittock DR, Russell JA, Walley KR. Beneficial effects of short-term vasopressin infusion during severe septic shock. *Anesthesiology.* 2002;96(3):576-582.
43. Bosch NA, Teja B, Wunsch H, Walkey AJ. Practice Patterns in the Initiation of Secondary Vasopressors and Adjunctive Corticosteroids during Septic Shock in the United States. *Ann Am Thorac Soc.* 2021;18(12):2049-2057.
44. Vail EA, Gershengorn HB, Hua M, Walkey AJ, Wunsch H. Epidemiology of Vasopressin Use for Adults with Septic Shock. *Ann Am Thorac Soc.* 2016;13(10):1760-1767.
45. Sacha GL, Kiser TH, Wright GC, et al. Association Between Vasopressin Rebranding and Utilization in Patients With Septic Shock. *Crit Care Med.* 2022;50(4):644-654.
46. Curtis N, Corapi J, Roberts R, Devlin JW. Rebranding of generic parenteral vasopressin: Effect on clinician practices and perceptions. *Am J Health Syst Pharm.* 2017;74(3):105-106.
47. Quan AT, Li F. Hyperinflation of Vasopressors (Vasopressin, Norepinephrine, Ephedrine, etc). *J Pharm Pract.* 2018;31(4):399-402.
48. Wu JY, Stollings JL, Wheeler AP, Semler MW, Rice TW. Efficacy and Outcomes After Vasopressin Guideline Implementation in Septic Shock. *Ann Pharmacother.* 2017;51(1):13-20.
49. Prevention CfDCa. Hospital Toolkit for Adult Sepsis Surveillance. 2018; https://www.cdc.gov/sepsis/pdfs/Sepsis-Surveillance-Toolkit-Mar-2018_508.pdf.
50. Matheny ME, Gelman HM, Souden M, Lu Z, DuVall SL, Gonsoulin ME. Challenges and Opportunities for Secondary Use of Observational Data Following an EHR Transition. *J Gen Intern Med.* 2023;38(Suppl 4):943-945.
51. Miake-Lye IM, Cogan AM, Mak S, et al. Transitioning from One Electronic Health Record to Another: A Systematic Review. *J Gen Intern Med.* 2023;38(Suppl 4):956-964.
52. Huang C, Koppel R, McGreevey JD, 3rd, Craven CK, Schreiber R. Transitions from One Electronic Health Record to Another: Challenges, Pitfalls, and Recommendations. *Appl Clin Inform.* 2020;11(5):742-754.
53. Self WH, Semler MW, Wanderer JP, et al. Balanced Crystalloids versus Saline in Noncritically Ill Adults. *N Engl J Med.* 2018;378(9):819-828.
54. Semler MW, Self WH, Wanderer JP, et al. Balanced Crystalloids versus Saline in Critically Ill Adults. *N Engl J Med.* 2018;378(9):829-839.

55. Bledsoe J, Peltan ID, Bunnell RJ, et al. Order Substitutions and Education for Balanced Crystalloid Solution Use in an Integrated Health Care System and Association With Major Adverse Kidney Events. *JAMA Netw Open.* 2022;5(5):e2210046.
56. Miller RR, 3rd, Dong L, Nelson NC, et al. Multicenter implementation of a severe sepsis and septic shock treatment bundle. *Am J Respir Crit Care Med.* 2013;188(1):77-82.
57. Clayton PD, Narus SP, Huff SM, et al. Building a comprehensive clinical information system from components: the approach at Intermountain Health Care. *Methods Inf Med.* 2003;42(1):1-7.
58. Kidney Disease: Improving Global Outcomes Acute Kidney Injury Work G. KDIGO clinical practice guidelines for acute kidney injury: summary of recommendation statements. *Kidney International Supplements.* 2012;2(1):8-12.
59. Hemming K, Kasza J, Hooper R, Forbes A, Taljaard M. A tutorial on sample size calculation for multiple-period cluster randomized parallel, cross-over and stepped-wedge trials using the Shiny CRT Calculator. *Int J Epidemiol.* 2020;49(3):979-995.
60. Hemming K, Kasza J, Hughes J. Power and Sample size for Cluster Randomised Trials. https://clusterrcts.shinyapps.io/rshinyapp/. Accessed July 9, 2022.
61. Peto R, Pike MC, Armitage P, et al. Design and analysis of randomized clinical trials requiring prolonged observation of each patient. I. Introduction and design. *Br J Cancer.* 1976;34(6):585-612.
62. Haybittle JL. Repeated assessment of results in clinical trials of cancer treatment. *Br J Radiol.* 1971;44(526):793-797.
63. Schulz KF, Grimes DA. Multiplicity in randomised trials II: subgroup and interim analyses. *Lancet.* 2005;365(9471):1657-1661.
64. *Adaptive Designs for Clinical Trials of Drugs and Biologics.* Food and Drug Administration Center for Drug Evaluation and Research;2019.
65. Janz DR, Semler MW, Joffe AM, et al. A Multicenter Randomized Trial of a Checklist for Endotracheal Intubation of Critically Ill Adults. *Chest.* 2018;153(4):816-824.
66. Semler MW, Janz DR, Russell DW, et al. A Multicenter, Randomized Trial of Ramped Position vs Sniffing Position During Endotracheal Intubation of Critically Ill Adults. *Chest.* 2017;152(4):712-722.
67. Janz DR, Semler MW, Lentz RJ, et al. Randomized Trial of Video Laryngoscopy for Endotracheal Intubation of Critically Ill Adults. *Crit Care Med.* 2016;44(11):1980-1987.
68. Driver BE, Semler MW, Self WH, et al. Effect of Use of a Bougie vs Endotracheal Tube With Stylet on Successful Intubation on the First Attempt Among Critically Ill Patients Undergoing Tracheal Intubation: A Randomized Clinical Trial. *JAMA.* 2021;326(24):2488-2497.
69. Myburgh JA, Seppelt IM, Goodman F, et al. Effect of Selective Decontamination of the Digestive Tract on Hospital Mortality in Critically Ill Patients Receiving Mechanical Ventilation: A Randomized Clinical Trial. *JAMA.* 2022.
70. Semler MW, Janz DR, Lentz RJ, et al. Randomized Trial of Apneic Oxygenation during Endotracheal Intubation of the Critically Ill. *Am J Respir Crit Care Med.* 2016;193(3):273-280.
71. Casey JD, Janz DR, Russell DW, et al. Bag-Mask Ventilation during Tracheal Intubation of Critically Ill Adults. *N Engl J Med.* 2019;380(9):811-821.

72. Young P, Bailey M, Beasley R, et al. Effect of a buffered crystalloid solution vs saline on acute kidney injury among patients in the intensive care unit: the SPLIT randomized clinical trial. *JAMA.* 2015;314(16):1701-1710.
73. Casey JD, Vaughan EM, Lloyd BD, et al. Protocolized Postextubation Respiratory Support to Prevent Reintubation: A Randomized Clinical Trial. *Am J Respir Crit Care Med.* 2021;204(3):294-302.
74. Semler MW, Wanderer JP, Ehrenfeld JM, et al. Balanced Crystalloids versus Saline in the Intensive Care Unit. The SALT Randomized Trial. *Am J Respir Crit Care Med.* 2017;195(10):1362-1372.
75. Investigators P, Australian New Zealand Intensive Care Society Clinical Trials Group, Alberta Health Services Critical Care Strategic Clinical Network, et al. Effect of Stress Ulcer Prophylaxis With Proton Pump Inhibitors vs Histamine-2 Receptor Blockers on In-Hospital Mortality Among ICU Patients Receiving Invasive Mechanical Ventilation: The PEPTIC Randomized Clinical Trial. *JAMA.* 2020;323(7):616-626.
76. Noto MJ, Domenico HJ, Byrne DW, et al. Chlorhexidine bathing and health care-associated infections: a randomized clinical trial. *JAMA.* 2015;313(4):369-378.
77. Semler MW, Casey JD, Lloyd BD, et al. Oxygen-Saturation Targets for Critically Ill Adults Receiving Mechanical Ventilation. *N Engl J Med.* 2022.
78. Janz DR, Casey JD, Semler MW, et al. Effect of a fluid bolus on cardiovascular collapse among critically ill adults undergoing tracheal intubation (PrePARE): a randomised controlled trial. *The Lancet Respiratory Medicine.* 2019;7(12):1039-1047.
79. Landsperger JS, Byram JM, Lloyd BD, Rice TW, Pragmatic Critical Care Research G. The effect of adhesive tape versus endotracheal tube fastener in critically ill adults: the endotracheal tube securement (ETTS) randomized controlled trial. *Crit Care.* 2019;23(1):161.
80. Casamento AJ, Serpa Neto A, Young M, et al. A Phase II Cluster-Crossover Randomized Trial of Fentanyl vs. Morphine for Analgosedation in Mechanically Ventilated Patients. *Am J Respir Crit Care Med.* 2021.
81. Huang SS, Septimus E, Kleinman K, et al. Chlorhexidine versus routine bathing to prevent multidrug-resistant organisms and all-cause bloodstream infections in general medical and surgical units (ABATE Infection trial): a cluster-randomised trial. *Lancet.* 2019;393(10177):1205-1215.
82. Ahmad T, Yamamoto Y, Biswas A, et al. REVeAL-HF: Design and Rationale of a Pragmatic Randomized Controlled Trial Embedded Within Routine Clinical Practice. *JACC Heart Fail.* 2021;9(6):409-419.
83. Ghazi L, O'Connor K, Yamamoto Y, et al. Pragmatic trial of messaging to providers about treatment of acute heart failure: The PROMPT-AHF trial. *Am Heart J.* 2023;257:111-119.
84. Melnick ER, Nath B, Dziura JD, et al. User centered clinical decision support to implement initiation of buprenorphine for opioid use disorder in the emergency department: EMBED pragmatic cluster randomized controlled trial. *BMJ.* 2022;377:e069271.
85. Casey JD, Beskow LM, Brown J, et al. Use of pragmatic and explanatory trial designs in acute care research: lessons from COVID-19. *Lancet Respir Med.* 2022;10(7):700-714.
86. Young PJ. Learning Healthcare Systems Will Protect Patients from Unscientific Practice Variation. *Ann Am Thorac Soc.* 2018;15(2):131-133.

87. Casey JD, Courtright KR, Rice TW, Semler MW. What can a learning healthcare system teach us about improving outcomes? *Curr Opin Crit Care.* 2021;27(5):527-536.
88. Dember LM, Lacson E, Jr., Brunelli SM, et al. The TiME Trial: A Fully Embedded, Cluster-Randomized, Pragmatic Trial of Hemodialysis Session Duration. *J Am Soc Nephrol.* 2019;30(5):890-903.
89. Ishani A, Leatherman SM, Woods P, et al. Design of a pragmatic clinical trial embedded in the Electronic Health Record: The VA's Diuretic Comparison Project. *Contemp Clin Trials.* 2022;116:106754.
90. Yap TL, Horn SD, Sharkey PD, et al. Effect of Varying Repositioning Frequency on Pressure Injury Prevention in Nursing Home Residents: TEAM-UP Trial Results. *Adv Skin Wound Care.* 2022;35(6):315-325.
91. MyTEMP Writing Committee. Personalised cooler dialysate for patients receiving maintenance haemodialysis (MyTEMP): a pragmatic, cluster-randomised trial. *Lancet.* 2022;400(10364):1693-1703.
92. Simon GE, Shortreed SM, Rossom RC, Penfold RB, Sperl-Hillen JAM, O'Connor P. Principles and procedures for data and safety monitoring in pragmatic clinical trials. *Trials.* 2019;20(1):690.
93. Irving E, van den Bor R, Welsing P, et al. Series: Pragmatic trials and real world evidence: Paper 7. Safety, quality and monitoring. *J Clin Epidemiol.* 2017;91:6-12.
94. Schoenfeld DA, Bernard GR, Network A. Statistical evaluation of ventilator-free days as an efficacy measure in clinical trials of treatments for acute respiratory distress syndrome. *Crit Care Med.* 2002;30(8):1772-1777.
95. Novack V, Beitler JR, Yitshak-Sade M, et al. Alive and ventilator free: a hierarchical, composite outcome for clinical trials in the acute respiratory distress syndrome. *Crit Care Med.* 2020;48(2):158-166.
96. Semler MW, Rice TW, Shaw AD, et al. Identification of Major Adverse Kidney Events Within the Electronic Health Record. *J Med Syst.* 2016;40(7):167.
97. Zavada J, Hoste E, Cartin-Ceba R, et al. A comparison of three methods to estimate baseline creatinine for RIFLE classification. *Nephrol Dial Transplant.* 2010;25(12):3911-3918.
98. Inker LA, Eneanya ND, Coresh J, et al. New Creatinine- and Cystatin C-Based Equations to Estimate GFR without Race. *N Engl J Med.* 2021;385(19):1737-1749.

APPENDIX A — Eligible vasopressor and associated equivalencies

Eligible vasopressors	Multiplier (conversion factor) to convert to norepinephrine base equivalents*	Equivalent doses*	
		Norepinephrine†	Other drug
Norepinephrine†	1	0.1 mcg/kg/min	N/A
Epinephrine	1	0.1 mcg/kg/min	0.1 mcg/kg/min
Dopamine	0.01	0.1 mcg/kg/min	10 mcg/kg/min
Phenylephrine	0.1	0.1 mcg/kg/min	1 mcg/kg/min
Vasopressin	3.333333334<br>0.055555556	0.1 mcg/kg/min	0.03 units/**min**<br>1.8 units/**hr**
Angiotensin II	10	0.1 mcg/kg/min	0.01 mcg/kg/min

* Adapted from: Goradia *et al*. Vasopressor dose equivalence: A scoping review and suggested formula. *J Crit Care*, 61; 2021: 233-240.

† Norepinephrine doses represent norepinephrine base.[18]

APPENDIX B —Randomized monthly strategy assignments and example trial timeline

Initial randomization sequence is pre-determined as shown. Each individual study hospital will be assigned the lower and higher vasopressin initiation strategies for an equal number of months. A potential hospital-level study timeline is shown. The time at which hospitals exit from the study will be based on the timing of each hospital's EMR transition as discussed in section 4.5.

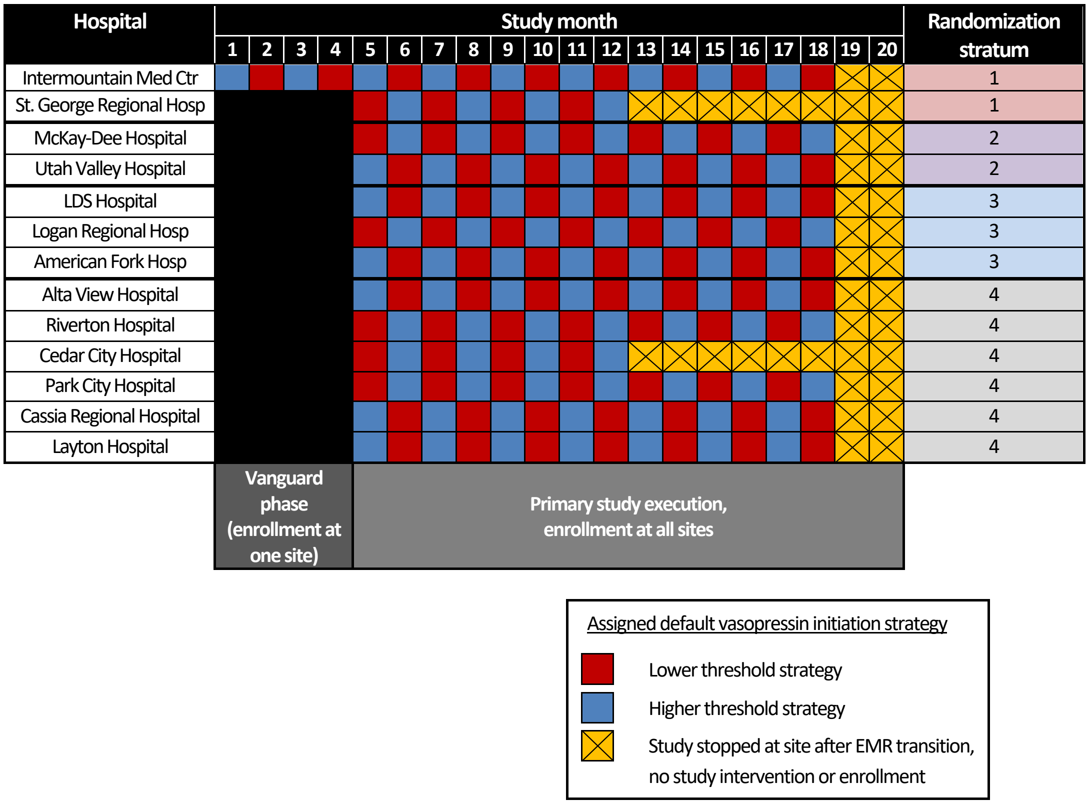

*

APPENDIX C — Conditions potentially altering vasopressin risk/benefit ratio

While there are no absolute contraindications to vasopressin administration other than theoretical allergy, listed conditions are noted as part of just-in-time education included within the order set for septic shock and, if identified via the EMR, result in "silencing" of the order set utilization reminder when vasopressors are ordered outside the septic shock order set for patients on antibiotics.

Condition	Method of identification for silencing	Rationale
Severe hyponatremia	Serum sodium <120 mEq/L	Potential exacerbation due to osmoregulatory effects of vasopressin.
STEMI	Condition listed as active problem in EMR	Possible exacerbation due to peripheral vasoconstriction by vasopressin.
NSTEMI	Condition listed as active problem in EMR and serum troponin >10 ng/mL	Possible exacerbation due to peripheral vasoconstriction by vasopressin.
Digital, limb, extremity, nose, ear, or other soft tissue ischemia	Condition listed as active problem in EMR	Possible exacerbation due to peripheral vasoconstriction by vasopressin.
Bowel or mesenteric ischemia	Condition listed as active problem in EMR	Possible exacerbation due to peripheral vasoconstriction by vasopressin.
Pregnancy	Pregnancy or related condition listed as active problem or hCG level above upper limit of normal	While vasopressin is used in practice, unknown pregnancy-related effects of vasopressin.
Nursing mother	Related condition listed as active problem in EMR	Unknown effects on breast milk production and unknown secretion into breast milk.
Systemic sclerosis	Condition listed as active or chronic problem in EMR	Potentially higher risk of digital ischemia in setting of treatment with pure peripheral vasoconstrictor
CREST syndrome	Condition listed as active or chronic problem in EMR	Potentially higher risk of digital ischemia in setting of treatment with pure peripheral vasoconstrictor
Raynaud syndrome	Condition listed as active or chronic problem in EMR	Potentially higher risk of digital ischemia in setting of treatment with pure peripheral vasoconstrictor

Abbreviations: EMR, electronic medical record; NSTEMI, non-ST-elevation myocardial infarction; STEMI, ST-elevation myocardial infarction;

APPENDIX D — Definitions of study outcomes and variables

Outcomes

Vasopressor-free days: Defined as the number of days alive and off vasopressor support from the time of trial enrollment to study day 28.[94,95] If a patient returns to vasopressor support and subsequently achieves vasopressor independence prior to day 28, vasopressor-free days will be counted from the end of the last period of vasopressor support to day 28 (the "last off" method). Patients discharged alive and off vasopressors will be assumed to remain vasopressor free through study day 28. Patients discharged alive and on vasopressors before day 28 will be assumed to remain on vasopressors through study day 28. A period of vasopressor dependence during a surgical procedure will not count against the vasopressor-free days calculation. If a patient was receiving vasopressors at day 28, vasopressor-free days will be zero. If a patient dies on or before day 28, vasopressor-free days will be -1.[95] The maximum number of vasopressor-free days for a patient is 28 which would be assigned if they were vasopressor independent on the day following study enrollment and remained alive meeting the definition of vasopressor independence to day 28.

Renal replacement-free days: Defined as the number of days alive and off renal replacement therapy — including intermittent hemodialysis, hemofiltration, or ultrafiltration; slow continuous ultrafiltration or dialysis; or continuous venovenous hemodialysis, hemofiltration, hemodiafiltration, or hemofiltration — from the time of trial enrollment to study day 28.[94,95] If a patient resumes renal replacement therapy and subsequently achieves renal replacement therapy independence prior to day 28, renal replacement therapy-free days will be counted from the end of the last period of renal replacement therapy to day 28 (the "last off" method). Patients discharged alive and off renal replacement therapy will be assumed to remain off through study day 28. Patients discharged alive on renal replacement therapy will be assumed to remain on renal replacement through study day 28. If a patient was receiving renal replacement therapy at day 28 or has end stage renal disease treated with hemodialysis prior to the index hospitalization, renal replacement therapy-free days will be zero. If a patient dies on or before day 28, renal replacement therapy-free days will be -1.[95] The maximum number of renal replacement therapy-free days for a patient is 29 which would be assigned if they were renal replacement therapy independent on the day of study enrollment and remained alive meeting the definition of renal replacement therapy independence to day 28.

Hospital-free days: Defined as the number of days alive and out of the hospital through study day 28. Value of outcome will be calculated analogous to vasopressor-free days, with patients who die on or before study day 28 assigned a value of -1 and patients still hospitalized on day 28 assigned a value of 0. Patients discharged to another acute care hospital (excluding psychiatric inpatient facility) or a long-term acute care facility will be assumed to remain hospitalized through study day 28. Patients discharged alive and who do not die on or before study day 28 will be assumed to remain out of the hospital through day 28.

ICU-free days: defined as the number of days alive and out of the ICU through study day 28. The value of the outcome will be calculated analogous to vasopressor-free days, with patients who die on or before study day 28 assigned a value of -1 and patients still hospitalized on day 28 assigned a value of 0. If a patient returns to the ICU and subsequently achieves ICU independence prior to day 28, ICU-free days will be counted from the end of the last period of ICU care to day 28 (the "last off" method). Patients discharged alive and who do not die on or before study day 28 will be assumed to remain out of the ICU through day 28.

Receipt of stress-dose steroids: While receiving vasopressors, any medication administrations of:

- IV hydrocortisone 50-100 mg;
- During any shortage of IV hydrocortisone occurring during the study period requiring therapeutic exchange of other corticosteroids in place of hydrocortisone at study hospitals, either:
    - Administration of fludrocortisone plus administration of an alternative corticosteroid at a dose consistent with institutional medication substitution guidelines (e.g., IV dexamethasone 8 mg or IV methylprednisolone 40 mg); or
    - Administration of an alternative corticosteroid at a dose consistent with institutional medication substitution guidelines (e.g., IV dexamethasone 8 mg or IV methylprednisolone 40 mg IV) plus no indication in medical record that corticosteroid was administered for an indication other than septic shock.

Volume of IV resuscitation fluids: Measured as the total volume of crystalloid fluids (normal saline, lactated Ringer's solution, PlasmaLyte®) or normal bicarbonate (150 mEq/L sodium bicarbonate in 5% dextrose solution or sterile water) administered at a rate of >150 ml/hr plus the volume of any IV albumin (5% or 25%) administered.

Other variables

Baseline creatinine: Baseline creatine will be obtained using an adaptation of a previously described hierarchical method.[54,96] If available, the lowest serum creatinine ≥24 hours and ≤1 year prior to hospital arrival will be used. If this data is unavailable, the lowest serum creatinine >1 year and ≤2 years prior to hospital arrival will be used. If there is no serum creatinine value available in either of these two windows, a baseline creatine may be estimated using the following formula[97]:

$$\text{Creatinine} = 0.74 - 0.2\ (\text{if female}) + 0.08\ (\text{if race is Black}) + 0.003\ \text{x}\ (\text{age in years})$$

Chronic kidney disease (CKD): Defined as end-stage renal disease managed with chronic hemodialysis or CKD stage 3, 4, or 5 based on a baseline creatinine glomerular filtration rate (GFR) <60 ml/min/1.73 m^2 or clinical documentation. GFR will be estimated from baseline serum creatinine using the 2021 version of the Chronic Kidney Disease Epidemiology (CKD-EPI) Collaboration equation.[98]

Chronic cardiovascular disease: Documented history of myocardial infarction or coronary artery disease, congestive heart failure, or peripheral vascular disease.

e-Appendix 3. Statistical analysis plan

VASSPR Trial

Vasopressin for Septic Shock Pragmatic Trial

STATISTICAL ANALYSIS PLAN (SAP)

**SAP Authors:**	Danielle Groat, PhD<br>Benjamin Brintz, PhD
**SAP Version:**	1.3
**SAP Date:**	April 14, 2025
**Principal Investigator:**	Ithan Peltan, M.D.
**Trial Steering Committee:**	Ithan Peltan, MD, MSc (chair)<br>Lindsay Leither, DO<br>Samuel Brown, MD, MS<br>Joseph Bledsoe, MD<br>Colin Grissom, MD
**Sponsor:**	Intermountain Foundation

Contents

1. INTRODUCTION 4

1.1 SYNOPSIS 4

1.1.1 Overall design 4

1.1.2 Objectives 4

1.2 METHODS 4

1.2.1 Study sites and study duration 4

1.2.2 Randomization and blinding 5

1.2.3 Population, enrollment, and treatment assignment 5

1.2.4 Intervention 6

1.2.5 Data collection 6

1.2.6 Primary outcome 6

1.2.7 Secondary outcome 6

1.2.8 Exploratory clinical outcomes 7

1.2.9 Exploratory safety outcomes 7

1.2.10 Statistical hypotheses 8

1.2.11 Power calculation 8

2 STATISTICAL ANALYSIS 9

2.1 INTERIM ANALYSES 9

2.2 STATISTICAL PRINCIPLES 10

2.2.1 Software 10

2.2.2 Level of significance 10

2.2.3 Withdrawal, missing data, and outliers 10

2.2.4 Baseline analysis 11

2.2.5 Primary outcome analysis 11

2.2.6 Secondary and exploratory outcome analyses 12

2.2.7 Exploratory analyses 12

2.2.8 Sensitivity analyses 12

2.2.9 Corrections for multiple testing 14

2.2.10 Post hoc analyses 14

3 APPENDIX 15

3.1 EVENT-FREE DAYS OUTCOME DEFINITIONS 15

3.1.1 Renal replacement-free days 15
3.1.2 Vasopressor replacement-free days 15
3.1.3 Hospital-free days 16
3.1.4 Intensive care unit (ICU)-free days 16

3.2 REVISION HISTORY 16

1. INTRODUCTION

Sepsis is a syndrome of life-threatening organ failure. The morbidity, mortality, and costs imposed by sepsis are enormous. Mortality reaches 30-40% for the 17% of patients with septic shock and climbs as high as 80% for refractory septic shock. Interventions that mitigate the adverse outcomes associated with refractory septic shock are a key knowledge gap.

International guidelines recommend the addition of vasopressin for septic shock patients with an inadequate response to first-line vasopressors. However, the guidelines' advice regarding vasopressin initiation threshold is vague. Inadequate data and escalation of vasopressin costs has led to conflicting septic shock management approaches and considerable practice variation for vasopressin treatment.

Practice variation unrelated to patient characteristics and preferences exposes patients to the risks and benefits of divergent treatment strategies but yields no new insights. By replacing arbitrary variation with structured variation via a pragmatic, embedded clinical trial, this study will generate knowledge that informs the care of patients with septic shock.

1.1 SYNOPSIS

1.1.1 Overall design

To compare the effectiveness of alternative strategies currently within the spectrum of usual care of septic shock management, we will conduct a multicenter, open-label, multiple cross-over, cluster-randomized, embedded, pragmatic clinical trial. The study will include 13 clusters and last up to 22 months (includes a 4-month vanguard phase, see section 1.2.1). Expected duration is 16 months. Preferred strategies for initiation of vasopressin at a lower versus a higher threshold will be implemented at the hospital level and alternate monthly per hospitals' randomized assignment.

1.1.2 Objectives

The objective of this study is to compare the effectiveness of septic shock treatment strategies utilizing lower versus higher thresholds for addition of vasopressin as a secondary vasopressor.

1.2 METHODS

1.2.1 Study sites and study duration

The thirteen hospitals within Intermountain Health's Desert and Canyons regions with intensive care units (ICUs) at the time of study initiation will be included in the trial. A single academic referral hospital, Intermountain Medical Center, will launch the trial 4 months prior to system-wide implementation. During this 4-month vanguard phase, electronic medical record-based interventions, implementation methods, data collection methods, and safety monitoring will be tested and optimized. After the vanguard phase, active study intervention and enrollment will begin at all study hospitals. The total planned duration of active enrollment will be

approximately 16 months (maximum 22 months) at the vanguard hospital and 6-12 months (maximum 18 months) at each of the other 12 study hospitals. The study team plans to determine the date on which study hospitals cease study enrollment based on each study hospital's transition from its current electronic medical record (EMR) system to a new EMR. Each hospital's study closure date will be selected such that the hospital has approximately equal time assigned to each vasopressin initiation strategy.

1.2.2 Randomization and blinding

In this multiple-crossover study design, each of the 13 study sites will be randomized to begin the study assigned to a lower or higher threshold strategy for the initiation of vasopressin as a secondary vasopressor. Block randomization will use a computer-generated random number and study sites will be stratified based on four levels of historical volumes of patients treated with septic shock. After each site's initial study month, the site will alternate monthly between the two vasopressin initiation strategies for the duration of the study.

Due to the pragmatic design of the trial, blinding of patients, clinicians, and study personnel to treatment assignment is not practical. Data will be collected via queries and will be reviewed while remaining agnostic to treatment assignment. Creation and any modification of the statistical analysis plan is performed by individuals without access to unblinded data. A statistician blinded to treatment assignment throughout the trial will conduct the final analyses.

1.2.3 Population, enrollment, and treatment assignment

Inclusion Criteria:

- Age ≥18 years
- Admitted to a study hospital emergency department (ED) or inpatient care unit
- Administration of vasopressor(s) for septic shock

Exclusion Criteria: None

All adult patients aged 18 years or older admitted to a study hospital emergency department or inpatient care unit who are administered vasopressor(s) for septic shock. Administration of vasopressors for septic shock is determined by concurrent 1) active vasopressor infusion, and 2) an active vasopressor administration order incorporating an indication for septic shock. The indication for vasopressor administration is documented by the ordering provider in real-time within the computerized order for vasopressor administration.

Enrollment: Patients will be considered enrolled in the study at the time when 1) the patient is admitted to a study hospital emergency department or inpatient care unit, and 2) a clinician has

entered an active order for vasopressors with an indication for suspected or confirmed septic shock, and 3) vasopressors are being infused. Day of enrollment is considered study day zero.

Treatment assignment: Patients' treatment assignment will be the assigned treatment strategy in place for the study hospital where they are admitted at the time they are enrolled.

1.2.4 Intervention

The intervention will be embedded in routine care, thus aiding adherence to the assigned strategy while allowing clinicians to tailor care for individual patients as needed. Order sets for septic shock management will be modified to include a pre-selected order for initiation of fixed-dose vasopressin infusion when the total dose of first-line vasopressor(s) reaches the assigned low or high threshold for the hospital and month. The lower threshold treatment strategy will involve initiating vasopressin 1.8 units/hour when other continuously-infused vasopressor medications reach a total dose of 0.1 mcg/kg/min norepinephrine (or equivalent). The higher threshold strategy will involve initiating vasopressin 1.8 units/hour when other continuously-infused vasopressor medications reach a total dose of 0.4 mcg/kg/min norepinephrine (or equivalent). Lower vs higher threshold order sets will be implemented at the hospital level and alternate monthly per hospitals' randomized assignment.

Use of either strategy, both of which are within the range of current usual care, is at the discretion of the patients' treating clinicians. Clinicians may choose to not order vasopressin or to initiate vasopressin at an alternative threshold per their interpretation of individual patient needs.

1.2.5 Data collection

Collection of patient demographic and clinical characteristics, treatments, and outcomes will be embedded in routine clinical care. Most data will be obtained via queries of Intermountain's carefully curated and comprehensive electronic data warehouse (EDW). Patient-level clinical, laboratory, diagnostic, and billing data across inpatient and outpatient care settings are fully linked. Mortality will be obtained from preexisting linkages to Utah State death records and the Social Security Death Index. Data from the EDW will be supplemented and validated as needed via review of the electronic medical record.

1.2.6 Primary outcome

The primary outcome is 28-day all-cause mortality, defined as death on or before study day 28, regardless of location at the time of death.

1.2.7 Secondary outcome

The key secondary outcome is renal replacement therapy-free days to day 28. Mortality on or before day 28 will be assigned -1. For patients with baseline end-stage renal failure receiving dialysis prior to the index hospitalization, potential values for this ordinal value will be 0 (dialysis is

recorded on or before study day 28) or -1 (mortality occurs on or before study day 28). Additional detail regarding the definition of this outcome is provided in Section 3.1.1.

1.2.8 Exploratory clinical outcomes

- In-hospital all-cause mortality
- 90-day all-cause mortality
- Vasopressor-free days to day 28
- Incidence of new renal replacement therapy — New receipt of renal replacement therapy after onset of septic shock. (Patients receiving renal replacement therapy prior to enrollment are excluded from this outcome.)
- ICU-free days to day 28
- Hospital-free days to day 28

Mortality data used for exploratory clinical outcomes will be collected through the specified outcome end point, regardless of the patient's location at the time of death. Other data for exploratory clinical outcomes are collected through hospital discharge. All interventions reflected in outcomes are performed as part of routine clinical care. Detailed definitions of event-free days outcomes are provided in Section 3.1.

1.2.9 Exploratory safety outcomes

Unless otherwise specified, safety outcomes are collected through hospital discharge. All laboratory or other diagnostic testing reflected in outcomes are performed as part of routine clinical care.

- Incidence of acute coronary syndrome — Documented new-onset clinical diagnosis of acute coronary syndrome or ST-elevation or non-ST-elevation myocardial infarction[1]
- Incidence of mesenteric ischemia — Documented new-onset clinical diagnosis of mesenteric ischemia[1]
- Incidence of soft tissue ischemia — Documented new-onset clinical diagnosis of extremity, nose, or ear ischemia[1]
- Incidence of vasopressor extravasation — Documented clinical diagnosis of vasopressor extravasation occurring after onset of septic shock
- Incidence of clinically-significant arrhythmia — Documented clinical diagnosis of clinically-significant arrhythmia (sustained ventricular tachycardia, reentrant

[1] Subjects with onset of the exploratory safety outcome before enrollment in the study will be excluded from analysis of this outcome

[supraventricular] tachycardia, atrial arrhythmia with rapid ventricular response requiring intervention, or new-onset atrial fibrillation or flutter) occurring after onset of septic shock

- Incidence of cardiogenic shock—Documented clinical diagnosis of cardiogenic shock occurring after onset of septic shock
- Incidence of cardiac arrest — Documented occurrence of a cardiac arrest with administration of chest compressions or defibrillation after onset of septic shock
- Incidence of severe hyponatremia — New-onset severe hyponatremia (serum sodium <120 mEq/L)[1]
- Maximum lactate — Maximum lactate (arterial or venous; mmol/L) from enrollment through study day 7
- Incidence of abnormal troponin — Serum troponin above upper limit of normal for assay from enrollment through study day 7[2]

1.2.10 Statistical hypotheses

The primary statistical hypotheses are as follows:

- Null hypothesis: There is no difference in 28-day all-cause mortality between patients randomized to the lower threshold strategy (0.1 mcg/kg/min) versus the higher threshold strategy (0.4 mcg/kg/min) for initiating the administration of vasopressin.
- Alternative hypothesis: There is a difference (2-sided) in 28-day all-cause mortality between subjects randomized to the two treatment strategies.

1.2.11 Power calculation

Based on historical data, the study hospitals average 11.7 patients per month per hospital with septic shock (range 1-45) of whom 8.25 patients per month (range 0.3-35) receive ≥0.1 mcg/kg/min norepinephrine (or equivalent). We conservatively estimated that the study will last 16 months — including 8-12 months of enrollment at all study sites and an additional 4-month vanguard phase at the largest-volume site (see study timeline depicted in Appendix B) — and will enroll approximately 2050 patients, including approximately 1445 patients with maximum vasopressor dose ≥0.1 mcg/kg/min norepinephrine (or equivalent). Power analyses employed the following additional assumptions:

- Historical patient volumes for each cluster

[2] Serum troponin thresholds (and potentially other laboratory values) will be determined based on normal lab values during the study period. Normal lab values are subject to change based on patient sex, study site, lab equipment, and other factors throughout the study period.

- 28-day mortality of 40% among patients receiving ≥0.1 mcg/kg/min norepinephrine (or equivalent) assigned to the higher vasopressin initiation treatment strategy (considered the control group)
- 28-day mortality of 21% for all patients (regardless of treatment assignment) who <0.1 mcg/kg/min norepinephrine (or equivalent)
- Within-cluster correlation of 0.003
- Randomization stratified by hospital patient volume (see Section 4.4 and Appendix B)
- No heterogeneity of the intervention effect across clusters
- A type 1 error rate of 0.05.

Simulation analyses based on these assumptions estimate that the trial will have 80% power to detect an absolute 5.4% difference in all-cause 28-day mortality (27.6% vs 33.0%) for the compared treatment groups.

2 STATISTICAL ANALYSIS

2.1 INTERIM ANALYSES

A Data and Safety Monitoring Board (DSMB) will be appointed to oversee the conduct of the trial and review safety endpoints every six months, beginning with availability of data for patients discharged within 6 months of study initiation. The DSMB will review one formal interim analysis for efficacy.

The DSMB will review the interim analysis for efficacy roughly 12 months after the study initiation and will include patients enrolled in the study and discharged within the first 8 months of the trial, including the vanguard phase. A conservative Haybittle-Peto stopping boundary (two-sided p-value <0.001) will be employed to test for between-group differences in the primary outcome. This will allow for the final analysis to proceed with the typical p-value threshold for statistical significance, a p-value ≤ 0.05. (Section 2.2.2)

There is no planned interim review for futility, and therefore no formal stopping guideline for futility. To protect patient safety, the DSMB will have the ability to stop the trial at any time for safety or other concerns, to require supplementary data, ask for additional interim analyses, or to request study protocol revisions.

Prior to the expected end of the trial, associated with study hospitals' transition to a new electronic medical record (EMR), the DSMB will review non-comparative (i.e., pooled) data on 1) monthly enrollment, 2) primary outcome incidence, 3) proportion of enrolled patients who received ≥0.1 mcg/kg/min norepinephrine or equivalent, and 4) rates of treatment strategy crossover and non-adherence. Lower than expected rates of patient enrollment, the incidence rate of the primary outcome, number of patients with shock treated with ≥0.1 mcg/kg/min norepinephrine or equivalent, or higher-than-expected treatment strategy crossover and non-

adherence could all decrease the study's power to detect a difference between the treatment strategies with the preplanned power analysis. Therefore, if meaningful differences from expected values for these factors are observed, and continuing the study beyond the planned duration is determined to be operationally feasible, the DSMB may request the unblinded biostatistician to re-estimate the sample size required to maintain the pre-specified power to detect the prespecified treatment effect. Based on this re-estimation, the DSMB may recommend extending the trial beyond the planned duration and/or resuming the trial after the study hospitals' EMR transition up to a maximum of 30 months of active enrollment and a maximum total trial duration of 4 years.

2.2 STATISTICAL PRINCIPLES

2.2.1 Software

All analyses will be performed using R version 4.0 or above or Stata version 16 or above.

2.2.2 Level of significance

The single interim analysis for efficacy will use the Haybittle-Peto stopping rules with a p-value < 0.001. Given the conservative stopping rule and the negligible amount of type-I error spent at the interim analysis, the significance threshold will remain at the standard p-value ≤ 0.05 for the final analyses (see also Section 2.1). No corrections will be made for multiple comparisons for the key secondary outcome or any of the exploratory outcomes.

2.2.3 Withdrawal, missing data, and outliers

All subjects that meet inclusion criteria will be included in the study, with analysis performed according to intention to treat principles. No withdrawal at either the site or patient level are expected due to the pragmatic, embedded nature of this trial.

Care and outcomes through the end of the index hospital encounter and study follow-up (including any subsequent episodes of shock meeting study entry criteria) contribute to subjects' study outcomes. For an individual patient, to ensure independence of observations, episodes of shock meeting study inclusion criteria that occur during distinct hospital encounters subsequent to the index hospital encounter will be included in safety monitoring reports for the Data and Safety Monitoring Board and a planned sensitivity analysis (see section 2.2.7) but will be excluded from other analyses.

We expect that there will not be missing outcome data for mortality. Missing outcome and treatment assignment data will not be imputed for any analysis. Missing data for patient-level covariates used in the analyses is possible but not expected. If data missingness for patient-level covariates is present, we will perform the primary, secondary, and exploratory outcome analysis after imputing using chained equations and complete case analyses will be performed as sensitivity analyses. See section 2.2.8.

For presentation of descriptive data, missing data will not be imputed and missingness will be reported.

Outliers will be reviewed for validity by a statistician blinded to treatment assignments. Outliers that are determined to be valid will be included.

2.2.4 Baseline analysis

For description of study sites (e.g., hospital type, size of ICU), discrete variables will be summarized by frequencies and percentages. Continuous variables will be summarized with mean and standard deviation and/or median and first and third interquartile range.

Description of baseline characteristics for patients will be presented by treatment group. Discrete variables will be summarized by frequencies and percentages. Continuous variables will be summarized with mean and standard deviation and/or median and first and third interquartile range.

Between-group comparisons, as appropriate, for continuous variables will employ the t-test with unequal variances or the Wilcoxon rank-sum test. As appropriate for categorical variables, between-group comparisons will use chi-squared or Fisher's exact test.

2.2.5 Primary outcome analysis

The primary analysis will be an intention-to-treat comparison of the two treatment strategies — low versus high threshold for the addition vasopressin as an adjunctive vasopressor — on 28-day all-cause mortality. We will use a generalized linear mixed-effects model using the binomial family with a logit link. The independent variable will be the treatment strategy assignment. Fixed effects will include patient-level covariates: age, sex, source of suspected or diagnosed of infection (pulmonary, urinary, GI/abdominal, skin, other/multiple), preexisting chronic kidney disease, preexisting heart disease, non-cardiovascular SOFA score, and initiation of vasopressors within 72 hours of hospital arrival. A random intercept for study hospital will also be included in the model. The treatment effect will be expressed as the adjusted marginal odds ratio and its 95% confidence interval together with the corresponding risk difference and its 95% confidence interval (if applicable), using the higher threshold strategy as the reference (control) arm. We will estimate risk differences by calculating the average difference between the model estimated probability of the outcome for each patient if they received the higher threshold and if they had received the lower threshold, using parametric bootstrapping to calculate 95% confidence intervals. An odds ratio less than 1.0 or a risk difference less than 0 indicates a decrease in mortality in the lower threshold arm, i.e., benefit from the lower threshold treatment strategy. The generalized linear model uses the Wald test to evaluate whether there is evidence of a non-zero effect of the exposure. A two-sided p-value ≤ 0.05 will be considered significant (Section 2.2.2).

2.2.6 Secondary and exploratory outcome analyses

Analyses of secondary and exploratory outcomes will use the analytical methods as outlined above for the primary outcome based on generalized linear mixed-effects model incorporating identical fixed effects and a hospital level random intercept. However, for analyses of event-free days outcomes, we will use proportional odds (i.e., ordinal) logistic regression models. Analysis of the maximum lactate laboratory values will use a linear regression model. We will not report p-values for exploratory outcomes.

2.2.7 Exploratory analyses

Using a similar generalized linear mixed-effects model as the primary analysis, including adjustment for patient-level covariates and random effects, we will assess heterogeneity of treatment effect for the primary outcome associated with patient-level covariates in the adjustment model by incorporating an interaction term between the covariate of interest and the treatment assignment. Planned covariates for heterogeneity of treatment effect evaluation include age (≤65 years vs. > 65 years), sex at birth (male vs female), source of infection suspected or diagnosed at time of study entry (pulmonary, urinary, intrabdominal, or other), preexisting chronic kidney disease (see protocol Appendix D), chronic cardiovascular disease (see protocol Appendix D), non-cardiovascular SOFA score (terciles), and time from hospital presentation to initial receipt of vasopressor (<72 hours vs. ≥ 72 hours). Continuous variables will remain so for analysis of effect modification but may be dichotomized (as presented in preceding parentheses in order of dichotomization preference) for data presentation. At the time of the planned interim analysis, we will review the specified variables for effect modification to ensure there are sufficient patients for meaningful analysis and make any necessary adjustments to the exploratory analyses described in this section, based on pooled data. The investigators making that assessment will remain blinded to treatment assignment.

To facilitate interpretation of analytic models and study, we will report intraclass correlation coefficients for the main analysis for the primary, secondary, and each exploratory outcome.

2.2.8 Sensitivity analyses

Pre-planned sensitivity analyses include:

Assessment for crossover and contamination effects,

- Strategy-ordered population: Repeat the primary and key secondary outcome analyses after restricting the cohort to enrolled patients for whom a study-specific threshold-based order vasopressin was entered.
- Strategy-eligible population: Repeat the primary and key secondary outcome analyses after restricting the cohort to enrolled patients with a maximum vasopressor dose of ≥0.07 mcg/kg/min of norepinephrine (or equivalent).

- Confirmed septic shock population: Repeat the primary and key secondary outcome analyses for patients who received ≥ 1 dose of antimicrobials in window of -30 to +2 hours from study entry and/or had a positive molecular test for an infection not necessarily amenable to antimicrobial treatment (i.e., SARS-CoV-2, influenza, respiratory syncytial virus, adenovirus, parainfluenza, metapneumovirus, non-COVID coronavirus, norovirus, or rotavirus) within 7 days preceding study entry or up to 2 hours after study entry.
- Time-dependent crossover reduction (analytic washout): Repeat the primary and key secondary outcome analyses while excluding patients who were enrolled during the final 2 days of each month, i.e., crossover period.
- Transfer-related crossover reduction: Repeat the primary and key secondary outcome analyses after excluding patients enrolled at hospitals with ICUs not staffed by in-person intensivist physicians (i.e., excluding patients at increased likelihood of transfer between hospitals and therefore potentially resulting in crossover).

Assessment for time and carryover effects:

- Repeat the primary outcome analysis after adding time as a covariate. The time variable will be a continuous variable indicating study month and modeled 1) as a continuous linear fixed effect, and 2) using restricted cubic splines to allow for non-linearity.
- Repeat the primary outcome analysis and include treatment by period interactions to assess potential carryover.
- Repeat the primary outcome analysis and include a random interaction for time and study hospital.

Other sensitivity analyses:

- Recurrent enrollment for individual patients: Repeat the primary and key secondary outcome analyses including subsequent eligible enrollments for an individual patient occurring >28 days after the initial enrollment.
- Unadjusted analysis: Repeat the primary and key secondary outcome analyses without adjusting for baseline patient covariates.
- Effect estimates: Where applicable, we will report conditional odds ratios as a sensitivity analysis for main analyses reporting adjusted marginal odds ratios.
- Missing data: If covariate or exploratory outcomes are missing in >1 eligible patient, complete case analyses will be performed as sensitivity analyses. See section 2.2.3.

2.2.9 Corrections for multiple testing

The primary and key secondary outcome will not be adjusted for multiple comparisons. All exploratory analyses (e.g., clinical and safety) will be considered hypothesis-generating and no corrections for multiple comparisons will be performed.

2.2.10 Post hoc analyses

If investigators or reviewers introduce analyses in addition to those described herein, these will be noted as post-hoc and will be considered hypothesis-generating.

3 APPENDIX

3.1 EVENT-FREE DAYS OUTCOME DEFINITIONS

3.1.1 Renal replacement-free days

Renal replacement-free days is defined as the number of days alive and off renal replacement therapy — including intermittent hemodialysis, hemofiltration, or ultrafiltration; slow continuous ultrafiltration or dialysis; or continuous venovenous hemodialysis, hemofiltration, hemodiafiltration, or hemofiltration — from the time of trial enrollment to study day 28. If a patient resumes renal replacement therapy and subsequently achieves renal replacement therapy independence prior to day 28, renal replacement therapy-free days will be counted from the end of the last period of renal replacement therapy to day 28 (the "last off" method). Patients discharged alive and off renal replacement therapy will be assumed to remain off through study day 28. Patients discharged alive on renal replacement therapy will be assumed to remain on renal replacement through study day 28. If a patient was receiving renal replacement therapy at day 28 or has end stage renal disease treated with hemodialysis prior to the index hospitalization, renal replacement therapy-free days will be zero. If a patient dies on or before day 28, renal replacement therapy-free days will be -1. The maximum number of renal replacement therapy-free days for a patient is 29 which would be assigned if they were renal replacement therapy independent on the day of study enrollment and remained alive meeting the definition of renal replacement therapy independence to day 28.

3.1.2 Vasopressor replacement-free days

Vasopressor-free days is defined as the number of days alive and off vasopressor support from the time of trial enrollment to study day 28. If a patient returns to vasopressor support and subsequently achieves vasopressor independence prior to day 28, vasopressor-free days will be counted from the end of the last period of vasopressor support to day 28 (the "last off" method). Patients discharged alive and off vasopressors will be assumed to remain vasopressor free through study day 28. Patients discharged alive and on vasopressors before day 28 will be assumed to remain on vasopressors through study day 28. A period of vasopressor dependence during a surgical procedure will not count against the vasopressor-free days calculation. If a patient was receiving vasopressors at day 28, vasopressor-free days will be zero. If a patient dies on or before day 28, vasopressor-free days will be -1. The maximum number of vasopressor-free days for a patient is 28 which would be assigned if they were vasopressor independent on the day following study enrollment and remained alive meeting the definition of vasopressor independence to day 28.

3.1.3 Hospital-free days

Hospital-free days is defined as the number of days alive and out of the hospital through study day 28. The value of outcome will be calculated analogous to vasopressor-free days, with patients who die on or before study day 28 assigned a value of -1 and patients still hospitalized on day 28 assigned a value of 0. Patients discharged to another acute care hospital (excluding psychiatric inpatient facility) or a long-term acute care facility will be assumed to remain hospitalized through study day 28. Patients discharged alive and who do not die on or before study day 28 will be assumed to remain out of the hospital through day 28.

3.1.4 Intensive care unit (ICU)-free days

ICU-free days is defined as the number of days alive and out of the ICU through study day 28. The value of the outcome will be calculated analogous to vasopressor-free days, with patients who die on or before study day 28 assigned a value of -1 and patients still hospitalized on day 28 assigned a value of 0. If a patient returns to the ICU and subsequently achieves ICU independence prior to day 28, ICU-free days will be counted from the end of the last period of ICU care to day 28 (the "last off" method). Patients discharged alive and who do not die on or before study day 28 will be assumed to remain out of the ICU through day 28.

3.2 REVISION HISTORY

Version and date	Description of change and brief rationale	Sections
Version 1.0 (29 May 2024)	N/A	N/A [Original version]
Version 1.1 (6 Feb 2025)	Revise plan for management of missing covariate data to clarify that missingness for data necessary for planned analyses is not expected. Analyses will be completed after multiple imputation if unexpected missingness does occur with complete case analyses completed as sensitivity analysis.	Sections 2.2.3 & 2.2.8
	Specify that, if applicable, odds ratios will be reported alongside their corresponding risk differences for the primary analysis and other analyses and describe model-based estimation of risk differences.	Section 2.2.5
	Add a sensitivity analysis including subsequent enrollments events for patients enrolled in the trial >1 time	Section 2.2.8

Version and date	Description of change and brief rationale	Sections
Version 1.2 (8 Apr 2025)	Consistent with original plan as described in Section 2.1, revise language in Section 2.2.2 to clarify that only a single formal interim analysis for efficacy will be performed.	Section 2.2.2
	Clarify that analyses models will incorporate a random *intercept* for study hospital.	Sections 2.2.5 and 2.2.6
	Clarify that the main analysis for the primary outcome (and other outcomes) will report adjusted marginal effect estimates	Section 2.2.5
	Specify that the intraclass correlation coefficient will be reported for the main analysis of all outcomes	Section 2.2.7
	Specify that a sensitivity analysis will report conditional odds ratios as a sensitivity analysis for main analyses reporting adjusted marginal odds ratios	Section 2.2.8
Version 1.3 (14 Apr 2025)	Specify methods — also described in the original trial protocol — for analysis of subsequent episodes of septic shock for an individual patient.	Section 2.2.3

e-Appendix 4. Study variables

VASSPR TRIAL VARIABLE LIST

Explanation of study related events and windows:

- **Study entry time:** Date/time where all entry criteria are met (including admission to a study hospital or ED).
- **Study entry day:** Enrollment day = Day 0 of study = Calendar day where all entry criteria are met
- **Study day:** Refers to calendar day, not rolling 24h windows
- **Overall/index encounter arrival:** First arrival to any Intermountain hospital for encounter continuous with encounter during which patient enters the study**.**
- **Overall/index encounter window:** Begins with first Intermountain hospital arrival (even if not a study hospital) for encounter that is continuous with the encounter during which patient enters the study and ends with final hospital discharge from encounter continuous with the encounter during which the patient enters the study.
- **Baseline:** Last available values preceding study entry date/time

Data sources: Data sources described in the table represent primary data sources. All data elements are evaluated for discrepancies, potentially invalid data, and missingness and are subject to query- and/or manual review-based methods for corrections as needed.

Variable	Definition/comments	Primary data source
**Study ID code**	Study ID code	N/A
**FIN**	Encounter ID code at time patient met study entry criteria	Query
**MRN/EMPI**	Patient medical record number	Query
**Hospital**	Hospital where patient was located when they met study entry criteria	Query
**Treatment assignment**	Treatment assignment (treatment assigned to study hospital where patient was located at the time patient met study entry criteria)	Identified from hospital assignment for corresponding to patient's hospital location at study entry
**Overall encounter ED admit date/time**	ED arrival date/time **for overall/index encounter**	Query with manual review of potential discrepancies
**Overall encounter hospital admit date/time**	Hospital admission date/time **for overall encounter**.	Query with manual review of potential discrepancies
**Overall encounter arrival date/time**	Date/time of first arrival to an Intermountain hospital **for overall encounter** (first event of index encounter ED arrival or index encounter hospital admit)	Query with manual review of potential discrepancies
**Study hospital arrival date/time**	Date/time of arrival to the **study hospital where patient met inclusion criteria**	Query with manual review of potential discrepancies
**Study hospital admit date/time**	Hospital admission date/time to the **study hospital where patient met inclusion criteria**	Query with manual review of potential discrepancies
**Study entry date/time**	First date/time during encounter that patient met all study inclusion criteria	Query with manual review of potential discrepancies
**Overall encounter first vasopressor date/time**	First administration of vasopressors **for overall encounter**	Query with manual review of potential discrepancies
**Study hospital first vasopressor date/time**	First administration of vasopressors at **study hospital where patient met study entry criteria**	Query with manual review of potential discrepancies
**Study vasopressin order date/time**	First order entry date/time for study-specific threshold -based vasopressin order	Query with manual review of potential discrepancies

Variable	Definition/comments	Primary data source
**Threshold date/time**	First date/time **after study entry** where vasopressor dose reached patient's assigned initiation threshold	Query with manual review of potential discrepancies
**Encounter vasopressin start date/time**	Date time patient first received vasopressin **for overall/index encounter**	Query with manual review of potential discrepancies
**Vasopressin first start date/time after study entry**	First date/time patient first received vasopressin **after study entry**	Query with manual review of potential discrepancies
**Final hospital discharge date/time**	Discharge date/time from final hospital in **overall encounter**	Query with manual review of potential discrepancies
**Interhospital transfer during index encounter**	Flag indicating that total study encounter involved more than one Intermountain facility	Query
**Age**	Age at study entry (years)	Query with manual review of missing and outlier data
**Sex**	Sex at birth	Query with manual review of missing data
**Race**	Self-reported race	Query
**Ethnicity**	Self-reported ethnicity	Query
**Insurance**	Insurance type	Query
**Language**	Preferred language	Query
**Marital status**	Marital status	Query
**Long-term care facility**	Admitted from long-term care facility	Manual chart review
**Charlson score**	Weighted Charlson Comorbidity Index	Calculated from query-based and manual chart review comorbidities
**Elixhauser score**	Weighted Elixhauser comorbidity score (von Walraven)	Calculated from query-based and manual chart review comorbidities
**Chronic dialysis**	Chronic dialysis prior to index encounter arrival	Query-assisted manual chart review
**Chronic cardiovascular disease (CVD)**	Chronic PAD, CHF, or CAD	Calculated from manual adjudication of component comorbidities
**Summative CKD status**	Manually adjudicated CKD status	Query-assisted manual chart review
**Charlson-Myocardial infarction**	Charlson comorbidity-Myocardial infarction	Query-assisted manual chart review
**Charlson-CHF**	Charlson comorbidity-Congestive heart failure	Query-assisted manual chart review
**Charlson-Paraplegia or hemplegia**	Charlson comorbidity-Paraplegia or hemiplegia	Query
**Charlson-Cerebrovascular disease**	Charlson comorbidity-Cerebrovascular disease	Query
**Charlson-Dementia**	Charlson comorbidity-Dementia	Query
**Charlson-Chronic pulmonary disease**	Charlson comorbidity-Chronic pulmonary disease	Query
**Charlson-Mild liver disease**	Charlson comorbidity-Mild liver disease	Query
**Charlson-Mod or severe liver disease**	Charlson comorbidity-Mod or severe liver disease	Query
**Charlson-Peripheral vascular disease**	Charlson comorbidity-Peripheral vascular disease	Query-assisted manual chart review
**Charlson-Renal disease**	Charlson comorbidity-Renal disease	Query-assisted manual chart review
**Charlson-Diabetes without complications**	Charlson comorbidity-Diabetes without complications	Query
**Charlson-Diabetes with complications**	Charlson comorbidity-Diabetes with complications	Query
**Charlson-Connective tissue disease**	Charlson comorbidity-Connective tissue dz	Query
**Charlson-Peptic ulcer disease**	Charlson comorbidity-Peptic ulcer disease	Query
**Charlson-Metastatic cancer**	Charlson comorbidity-Metastatic cancer	Query
**Charlson-Cancer**	Charlson comorbidity-Cancer	Query
**Charlson-HIV/AIDS**	Charlson comorbidity-HIV/AIDS	Query
**Elixhauser-CHF**	Elixhauser comorbidity-Congestive heart failure	Query-assisted manual chart review
**Elixhauser-Arrhythmia**	Elixhauser comorbidity-Arrhythmia	Query
**Elixhauser-Cardiac valvular disease**	Elixhauser comorbidity-Cardiac valvular disease	Query
**Elixhauser-Pulm circulatory disease**	Elixhauser comorbidity-Pulmonary circulatory disease	Query

Variable	Definition/comments	Primary data source
**Elixhauser-Peripheral vascular disease**	Elixhauser comorbidity-Peripheral vascular disease	Query-assisted manual chart review
**Elixhauser-Hypertension (uncomplicated)**	Elixhauser comorbidity-Hypertension (uncomplicated)	Query
**Elixhauser-Hypertension (complicated)**	Elixhauser comorbidity-Hypertension (complicated)	Query
**Elixhauser-Paralysis**	Elixhauser comorbidity-Paralysis	Query
**Elixhauser-Other neurological disorder**	Elixhauser comorbidity-Other neurological disorder	Query
**Elixhauser-Chronic pulmonary disease**	Elixhauser comorbidity-Chronic pulmonary disease	Query
**Elixhauser-Diabetes (uncomplicated)**	Elixhauser comorbidity-Diabetes (uncomplicated)	Query
**Elixhauser-Diabetes (complicated)**	Elixhauser comorbidity-Diabetes (complicated)	Query
**Elixhauser-Hypothyroidism**	Elixhauser comorbidity-Hypothyroidism	Query
**Elixhauser-Renal failure**	Elixhauser comorbidity-Renal failure	Query-assisted manual chart review
**Elixhauser-Liver disease**	Elixhauser comorbidity-Liver disease	Query
**Elixhauser-Peptic ulcer disease without bleeding**	Elixhauser comorbidity-Peptic ulcer disease without bleeding	Query
**Elixhauser-HIV/AIDS**	Elixhauser comorbidity-HIV/AIDS	Query
**Elixhauser-Lymphoma**	Elixhauser comorbidity-Lymphoma	Query
**Elixhauser-Metastatic cancer**	Elixhauser comorbidity-Metastatic cancer	Query
**Elixhauser-Solid tumor without metastasis**	Elixhauser comorbidity-Solid tumor without metastasis	Query
**Elixhauser-Rheumatoid arthritis**	Elixhauser comorbidity-Rheumatoid arthritis	Query
**Elixhauser-Coagulopathy**	Elixhauser comorbidity-Coagulopathy	Query
**Elixhauser-Obesity**	Elixhauser comorbidity-Obesity	Query
**Elixhauser-Weight loss**	Elixhauser comorbidity-Weight loss	Query
**Elixhauser-Fluid & electrolyte disorders**	Elixhauser comorbidity-Fluid & electrolyte disorders	Query
**Elixhauser-Blood loss anemia**	Elixhauser comorbidity-Blood loss anemia	Query
**Elixhauser-Deficiency anemia**	Elixhauser comorbidity-Deficiency anemia	Query
**Elixhauser-Alcohol abuse**	Elixhauser comorbidity-Alcohol abuse	Query
**Elixhauser-Substance abuse**	Elixhauser comorbidity-Substance abuse	Query
**Elixhauser-Psychoses**	Elixhauser comorbidity-Psychoses	Query
**Elixhauser-Depression**	Elixhauser comorbidity-Depression	Query
**Baseline GCS**	Glasgow Coma Scale score (GCS) last-available value preceding study entry	Query with manual review of missing data
**Baseline heart rate**	Heart rate (HR; beats/min) last-available value preceding study entry	Query with manual review of missing or outlier data
**Baseline temperature**	Temperature (Celsius) last-available value preceding study entry	Query with manual review of missing or outlier data
**Baseline respiratory rate**	Respiratory rate (RR; breaths/min) last-available value preceding study entry	Query
**Baseline systolic blood pressure**	Systolic blood pressure (SBP; mmHg) last-available value preceding study entry	Query
**Baseline diastolic blood pressure**	Diastolic blood pressure (DBP; mmHg) last-available value preceding study entry	Query
**Baseline mean arterial pressure**	Mean arterial pressure (MAP; mmHg). last-available value preceding study entry	Query
**Baseline lactate**	Serum or whole blood lactate (mmol/L) last-available value preceding study entry	Query
**Baseline sodium**	Sodium (mEq/L) last-available value preceding study entry	Query

Variable	Definition/comments	Primary data source
**Baseline creatinine**	Creatinine (mg/dL) last-available value preceding study entry	Query
**Baseline troponin I**	Troponin I (ng/mL) last-available value preceding study entry	Query
**Baseline high-sensitivity troponin T**	High-sensitivity troponin T (pg/mL = ng/L); last-available value preceding study entry	Query
**Baseline white blood cell count**	White blood count (WBC; 1000/dL); acceptable to use value within 6 hour after entry time.	Query
**Baseline bilirubin**	Serum bilirubin (mg/dL)	Query
**Baseline platelets**	Platelet count (1000/dL)	Query
**Baseline INR**	International Normalized Ratio	Query
**Non-cardiovascular SOFA score**	Non-cardiovascular SOFA score (range 0-20), calculated last-available values preceding study entry	Calculated from queried and manually obtained data
**Acute physiology score (APS)**	Calculated using last-available value preceding study entry or, if this is missing, within 24h after entry time.	Calculated from queried and manually obtained data
**Date/time of first E2 antibiotics during encounter**	Date/time of first IV or IV-equivalent antimicrobial administration during encounter	Query
**Date/time of antibiotics (any) during encounter**	Date/time of first antimicrobial administration via any route during encounter	Query
**COVID-19 positive**	COVID-19 positive (antigen or PCR) during overall index encounter or within 14 days preceding initial arrival for encounter	Query
**ED disposition**	Disposition from first ED during overall encounter	Query with manual review of potential discrepancies
**Hospital disposition**	Hospital disposition for **overall encounter**	Query with manual review of potential discrepancies
**Source of infection present study entry**	Source of infection that patient's clinical team suspected or diagnosed <u>at time of study entry</u>	Manual abstraction, with 5% random validation and investigator adjudication of disagreement
**Final adjudicated presence/source of infection**	Source of infection that was present at study entry as determined using all available data	Manual abstraction, with 5% random validation and investigator adjudication of disagreement
**Death date**	Date (no time component) of death	Combined query and manual review
**Hospital mortality**	Patient died in hospital	Derived from hospital discharge status
**28-day mortality**	Mortality on or before study day 28 (note that enrollment day = day 0)	Derived from death date
**90-day mortality**	Mortality on or before study day 90 (note that enrollment day = day 0)	Derived from death date
**1-year mortality**	Mortality on or before 1 year from study entry	Derived from death date
**RRT-free days**	Days alive and free of dialysis after study entry (calendar days)	Calculated from query and manually-abstracted data regarding chronic and inpatient dialysis
**RRT after enrollment**	Patient received renal replacement therapy (RRT) after enrollment	Query
**Last day of RRT during encounter**	Last intermittent hemodialysis, hemofiltration, or ultrafiltration; slow continuous ultrafiltration or dialysis; or continuous venovenous hemodialysis, hemofiltration, hemodiafiltration, or hemofiltration	Query

Variable	Definition/comments	Primary data source
**RRT continued after hospital discharge**	Among hospital survivors not on chronic dialysis who received ≤4 days from hospital dischargel, did patient continue dialysis after discharge	Manual abstraction
**New RRT after enrollment**	New receipt of renal replacement therapy after enrollment (excludes subjects receiving renal replacement therapy prior to enrollment)	Calculated from query and manually-abstracted data regarding chronic and inpatient dialysis
**ICU-free days**	ICU-free days to day 28 after study entry (calendar days)	Calculated from query and manually-verified data
**ICU length of stay for overall encounter**	ICU length of stay for overall encounter (round to hours)	Calculated from query and manually-verified data
**ICU length of stay after enrollment**	ICU length of stay after study entry (round to hours)	Calculated from query and manually-verified data
**Hospital-free days**	Hospital-free days to day 28 after study entry (calendar days)	Calculated from query and manually-verified data
**Hospital length of stay for overall encounter**	ICU length of stay for overall encounter (round to hours)	Calculated from query and manually-verified data
**Hospital length of stay after enrollment**	ICU length of stay after study entry (round to hours)	Calculated from query and manually-verified data
**Vasopressor-free days**	Vasopressor-free days to day 28 after study entry (calendar days)	Calculated from query and manually-verified data
**Mechanical ventilation**	Invasive mechanical ventilation (outside OR) on/after study entry	Query
**Elevated troponin**	Troponin I or high-sensitivity troponin above upper limit of normal after study entry but before discharge	Query
**Maximum troponin I**	Peak troponin I after study entry but before discharge	Query
**Maximum high-sensitivity troponin**	Peak high-sensitivity troponin T after study entry but before discharge	Query
**New moderate lactate elevation**	New moderate lactate (arterial blood gas, venous blood gas, or venous lactate >5 mmol/L after study entry but before discharge and ≤5 mmol/L or missing before study entry)	Query
**New severe lactate elevation**	New moderate lactate (arterial blood gas, venous blood gas, or venous lactate >10 mmol/L after study entry but before discharge and ≤10 mmol/L or missing before study entry)	Query
**Maximum lactate from enrollment through day 7**	Leave blank if lactate never checked after enrollment. Ends at day 7 or discharge, whichever occurs first	Query
**New moderate hyponatremia**	New moderate hyponatremia (serum sodium <130 mEq/L after study entry but before discharge and ≥130 or missing before study entry)	Query
**New severe hyponatremia**	New severe hyponatremia (serum sodium <120 mEq/L after study entry but before discharge and ≥120 or missing before study entry)	Query
**Lowest day 0 sodium**	Lowest sodium (mEq/L) on study day #0	Query
**Lowest day 1 sodium**	Lowest sodium (mEq/L) on study day #1	Query
**Lowest day 2 sodium**	Lowest sodium (mEq/L) on study day #2	Query
**Lowest day 3 sodium**	Lowest sodium (mEq/L) on study day #3	Query
**Lowest day 4 sodium**	Lowest sodium (mEq/L) on study day #4	Query
**Lowest day 5 sodium**	Lowest sodium (mEq/L) on study day #5	Query
**Lowest day 6 sodium**	Lowest sodium (mEq/L) on study day #6	Query
**Lowest day 7 sodium**	Lowest sodium (mEq/L) on study day #7	Query

Variable	Definition/comments	Primary data source
**Highest day 0 lactate**	Highest lactate (mmol/L) on study day #0	Query
**Highest day 1 lactate**	Highest lactate (mmol /L) on study day #1	Query
**Highest day 2 lactate**	Highest lactate (mmol /L) on study day #2	Query
**Highest day 3 lactate**	Highest lactate (mmol mmol /L) on study day #3	Query
**Highest day 4 lactate**	Highest lactate (mmol /L) on study day #4	Query
**Highest day 5 lactate**	Highest lactate (mmol /L) on study day #5	Query
**Highest day 6 lactate**	Highest lactate (mmol /L) on study day #6	Query
**Highest day 7 lactate**	Highest lactate (mmol /L) on study day #7	Query
**Vasopressin administered**	Vasopressin infusion administered on/after study entry	Query
**Study-specific vasopressin ordered**	Study-specific threshold-based vasopressin order entered by clinical team at any time during overall encounter (even if vasopressin never received)	Query
**Maximum vasopressor dose after enrollment before vasopressin**	Dose of non-vasopressin vasopressors (in norepinephrine equivalents) at initiation of vasopressin or, for patients never initiated on vasopressin, the patient's maximum total dose of vasopressors after enrollment. For patients who are on vasopressin at study entry, this is the dose of non-vasopressin vasopressors at study entry.	Query-assisted manual chart review
**Maximum vasopressor dose after enrollment**	Maximum dose (in NEE) of all vasopressors (including vasopressin) after enrollment	Query and query-assisted manual chart review
**Strategy adherence (time and range based)**	Adherence to assigned treatment strategy based on time- and dose-based definition	Calculated based on queried and manually abstracted data
**Stress-dose steroids prior to study entry**	Received stress-dose steroids from first encounter arrival through enrollment.	Query
**Stress-dose steroids (entry through study day 7)**	Received stress-dose steroids from enrollment through study day 7.	Query
**Elapsed time to stress-dose steroids**	Elapsed time (hours) from study enrollment to stress-dose steroids though hospital discharge.	Query
**Elapsed time from hospital arrival to stress-dose steroids (through discharge)**	Elapsed time (hours) from first encounter arrival to stress-dose steroids hospital discharge.	Query
**IV resuscitation fluid (within 24 hours of study enrollment)**	Volume (round to mL) of IV resuscitation fluid administered during the first 24 hours after enrollment.	Query
**IV resuscitation fluid (within 72 hours of study enrollment)**	Volume (round to mL) of IV resuscitation fluid administered during the first 72 hours after enrollment. (See Appendix D in study protocol for definitions.)	Query
**CVC present at enrollment or placement through study day 7**	Central venous access placed on/after study entry.	Query
**Elapsed time to CVC placement (through study day 7)**	Elapsed time (hours) from study enrollment to central venous catheter (CVC) placement up to study day 7. (See study Appendix D for definitions.). If CVC present at study entry, set "0" as value.	Query
**Clinical ACS diagnosis**	CRC chart review for clinical diagnosis of acute coronary syndrome	Query-assisted manual chart review
**Clinical mesenteric/bowel ischemia diagnosis (primary abstraction)**	CRC chart review for clinical diagnosis of mesenteric, intestinal, colon, or bowel ischemia	Query-assisted manual chart review
**Clinical soft tissue ischemia diagnosis**	CRC chart review for clinical diagnosis of ischemia of digits (fingers/toes), extremities, nose, or ears	Query-assisted manual chart review

Variable	Definition/comments	Primary data source
**Clinical vasopressor extravasation diagnosis**	CRC chart review for clinical diagnosis of vasopressor extravasation	Query-assisted manual chart review
**Clinical diagnosis of clinically-significant tachyarrhythmia**	CRC chart review for clinical diagnosis of clinically-significant tachyarrhythmia	Query-assisted manual chart review
**Clinical diagnosis of cardiogenic shock**	CRC chart review for clinical diagnosis of cardiogenic shock	Query-assisted manual chart review
**Cardiac arrest**	Cardiac arrest	Query-assisted manual chart review
**Potential pregnancy-associated safety event**	Potential pregnancy-associated safety event	Query-assisted manual chart review
**Timing of clinical ACS diagnosis**	Timing of clinical diagnosis of acute coronary syndrome after trial entry	Manual chart review
**Timing of clinical mesenteric/bowel ischemia diagnosis**	Timing of clinical diagnosis of mesenteric, intestinal, colon, or bowel ischemia after trial entry	Manual chart review
**Timing of clinical soft tissue ischemia diagnosis**	Timing of clinical diagnosis of ischemia of digits (fingers/toes), extremities, nose, or ears after trial entry	Manual chart review
**Timing of clinical vasopressor extravasation diagnosis**	Timing of clinical diagnosis of vasopressor extravasation after trial entry	Manual chart review
**Timing of clinical diagnosis of clinically-significant tachyarrhythmia**	Timing of clinical diagnosis of clinically-significant tachyarrhythmia after trial entry	Manual chart review
**Timing of clinical diagnosis of cardiogenic shock**	Timing of clinical diagnosis of cardiogenic shock after trial entry	Manual chart review
**Cardiac arrest**	Timing of cardiac arrest after trial entry	Manual chart review
**Discharge diagnosis codes**	Final ICD-10 discharge diagnosis	Query

e-Appendix 5. Data and safety monitoring board charter

Version 4.0 (28 October 2024)

VASSPR Trial

Vasopressin for Septic Shock Pragmatic Trial

DATA AND SAFETY MONITORING BOARD (DSMB) CHARTER

Sponsor: Intermountain Research and Medical Foundation

DSMB charter version: 4.0

Version date: October 28, 2024

Ithan Peltan
Electronically signed by: Ithan Peltan
Reason: I'm an approver
Date: Oct 30, 2024 11:42 MDT
10/30/2024

Ithan Peltan, MD, MSc **Date**
Principal Investigator

Todd W Rice, MD
Electronically signed by: Todd W Rice, MD
Reason: I'm an approver
Date: Oct 30, 2024 12:43 CDT
10/30/2024

Todd Rice, MD, MSc **Date**
DSMB Chair

Vasopressin in Septic Shock Pragmatic (VASSPR) Trial
Data and Safety Monitoring Board Charter

1. Introduction

This Charter is for the Data Safety and Monitoring Board (DSMB) for the study *Vasopressin in Septic Shock Pragmatic (VASSPR) Trial*, a pragmatic multicenter open-label embedded cluster-randomized cluster-crossover trial comparing a lower- versus a higher-threshold strategy for addition of fixed-dose vasopressin to first-line vasopressors for treatment of patients with refractory septic shock.

The Charter is intended to be a living document. The DSMB may wish to review this Charter at regular intervals to determine whether any changes should be considered. A record of changes relative to the initially approved Charter will be included as Appendix A.

2. Responsibilities of the DSMB

The DSMB is responsible for safeguarding the interests of study participants, assessing the safety and efficacy of study procedures, ensuring data quality, evaluating clinical equipoise, and for monitoring the overall conduct of the study. In order to do this effectively in a trial, the DSMB is expected to review the data in both aggregate form in open sessions and in an unblinded fashion in closed sessions.

The DSMB is an independent group advisory to the IRB and the investigators, and is required to provide recommendations about starting, continuing, and stopping the study. DSMB members must be cleared of any potential Conflict of Interest (COI) before participating in any meeting.

In addition, the DSMB is asked to make recommendations, as appropriate about:

- Benefit/risk ratio of procedures and participant burden
- Participant safety
- Notification of and referral for abnormal findings
- Clinical equipoise (at onset and throughout the study)
- Ability of study design to answer the primary question
- Adherence to protocol requirements
- Completeness, quality, and analysis of measurements
- Data and statistical analysis plan
- Amendments to the study protocol, including whether any new data from other sources affect the equipoise of the study being monitored
- Review efficacy data at the single interim analysis evaluating participants enrolled during the first 8 months of the trial
- Desirability of re-estimation of the study sample size and/or extension of study duration if this is deemed operationally feasible
- Review of the primary endpoint paper, methods and result sections

3. DSMB Composition

The DSMB will consist of members independent of the clinical trial with expertise in critical care, mechanical ventilation, biostatistics, ethics, and clinical trials. There will be 5 voting members of the DSMB, including the DSMB Chair. DSMB members are listed in Appendix B with their area of expertise. Dr. Todd Rice (Vanderbilt University) has been appointed as the DSMB Chair. The DSMB Executive Secretary (ES) will take minutes and work with the DSMB Chair to produce final recommendations and meeting report. An appropriate administrative assistant employed by the sponsor will serve as the ES and take minutes for the open session of DSMB meetings. They will prepare minutes for the meeting and send to the DSMB Chair who will add any additional minutes, reports, or recommendations from closed sessions of the DSMB. The DSMB Chair is responsible for assuring the accuracy and timely transmission of the final recommendations and DSMB's report. *Ad hoc* non-voting members may be added to the DSMB as necessary for single or multiple meetings to supplement expertise. Additional non-voting DSMB members may be included to support career development of early career researchers.

4. Conflict of Interest

Other than compensation from Intermountain Healthcare for their effort as DSMB members, the members of the DSMB should have no other significant relationship with the sponsor or investigators that could impair the members' ability to objectively review study data. DSMB members will provide a Conflicts of Interest Disclosure which includes current affiliations, if any, with pharmaceutical and biotechnology companies (e.g., stockholder, consultant), and any other relationship that could be perceived as a conflict of interest related to the study and/or associated with commercial interests pertinent to study objectives.

5. Confidentiality

All materials, discussions, and proceedings of the DSMB are privileged and confidential. DSMB members will use this information exclusively to accomplish the responsibilities of the DSMB. No communication of the deliberations or recommendations of the DSMB, either written or oral, may occur except as required for the DSMB to fulfill its responsibilities. Individual DSMB members are expected to maintain confidentiality regarding the study outside the DSMB (including, but not limited to the investigators, IRBs, regulatory agencies, or sponsor) except as authorized by the DSMB.

6. Organization and Interactions

The DSMB Chair will primarily communicate with the Principal Investigator (PI) of the VASSPR Trial, Ithan Peltan, MD, MSc, and with the co-PI, Lindsay Leither, DO. Outside of official DSMB activities, the members of the DSMB will have no direct involvement related to the trial with the study investigators or intervention. The PIs will ensure that the IRB is updated on communication between the DSMB and VASSPR Trial Pis. The VASSPR Study Pis will communicate with the VASSPR Trial Steering and Protocol Committees to discuss or disseminate DSMB communication to other study investigators. The VASSPR Study organization and key roles are listed below:

PI:	Ithan Peltan, MD, MSc
Co-PI:	Lindsay Leither, DO
Steering Committee:	Ithan Peltan, MD, MSc (chair)<br>Lindsay Leither, DO<br>Colin Grissom, MD<br>Samuel Brown, MD, MS<br>Joseph Bledsoe, MD

7. Initial DSMB Meeting

The first meeting of the DSMB will address organizational issues. This meeting will formally convene the DSMB.

The purposes of the first meeting are to:

- Introduce the members of the DSMB
- Formally review, modify (if needed), and approve the DSMB Charter and plan for communication between the trial team and DSMB.
- Provide an overview of the VASSPR Trial activities.

8. Scheduling and Timing of Subsequent DSMB Meeting

Approval by the DSMB and Institutional Review Board (IRB) will be required prior to initiation of the clinical trial. Prior to opening enrollment of the trial, the DSMB will meet one or more times to conduct the following business:

- Review the trial protocol and make recommendations for changes related to study execution or human subjects' safety and ethics;
- Approve the trial protocol;
- Review and provide feedback on the reporting shell for use in data and safety monitoring during the trial.

Scheduled meetings of the DSMB for interim trial data and safety monitoring will begin approximately six months after trial enrollment opens, and thereafter repeat approximately every 6 months until study completion. The DSMB will review adverse event data, other safety data, quality and completeness of study data, and enrollment data at each meeting to ensure proper study conduct. It will also conduct an

ongoing assessment of clinical equipoise in the study. Study personnel should provide any new literature particularly pertinent to the study, along with their recommendation as to whether it affects the study conduct or design. Based on an overall assessment of risk and review of the data, the Board will make recommendations, including whether the study should continue and/or be modified.

One formal interim analysis meeting will be scheduled for the DSMB to review efficacy data from the participants enrolled during the first 8 months of the trial. This interim analysis meeting will be scheduled to accommodate the accumulation of data from study follow-up for these patients

The DSMB will also review and approve the trial statistical analysis plan prior to the planned interim analysis.

The DSMB may convene additional *ad hoc* meetings as needed upon determination of the DSMB chair, either alone or in consultation with the trial PI and/or trial steering committee.

9. Format and Content of Formal DSMB Meetings

DSMB meeting may be held in person or remotely via videoconference. The DSMB will review adverse event data, other safety data, quality and completeness of study data, and enrollment data at each meeting to ensure proper study conduct. It will also conduct an ongoing assessment of clinical equipoise in the study. Study personnel will provide any new literature specifically pertinent to the study, along with their recommendation as to whether it affects the study conduct or design. A single interim efficacy analysis will be performed after primary trial outcome data are available for patients enrolled during the first 8 months of the planned approximately 18 month trial duration. The DSMB will not conduct interim futility analyses for the primary trial outcome. Based on an overall assessment of risk and review of the data, the Board will make recommendations, including whether the study should continue and/or be modified.

The expertise of the attending DSMB voting members should be appropriate for the agenda of the meeting. It's expected that all DSMB members will attend every meeting, but this may not always be possible. The Board Chair must be present at all meetings. A quorum for a live meeting will occur when >50% of voting members of the DSMB are present. Meeting will not be held without a quorum or without members whose expertise is deemed essential to the planned agenda by the DSMB chair. All standing Monitoring Board members are voting members. The Board may also decide in advance whether to award *ad hoc* members voting privileges for a specific meeting.

The agenda for DSMB meetings and calls will be drafted by the study biostatistician in consultation with the study PI and DSMB Chair. The agenda, prespecified routine meeting materials, and specially-requested information, reports, or materials will be distributed by the PI or their delegate for open meeting material and the unblinded statistician for closed meeting material one week before each meeting. Planned and *ad hoc* meetings of the DSMB subsequent to the initial organizational meeting will be organized as follows:

(1) **Introductory Session:** Participant roll call and as-needed introductions, confirmation of a quorum, confidentiality reminder, and a conflict of interest review for all DSMB members initiated by the ES or Chair. If a new conflict is reported, the Chair (with input from the study team as appropriate) will determine if the conflict limits the ability of the DSMB member to participate in the discussion.

Examples of conflict of interest include significant collaborations in publications or other activities during the past few years, employee reporting relationships, or financial or intellectual stake in the research to be monitored.

(2) **Open Session:** Open sessions will be attended by the DSMB Chair and all DSMB members, the Executive Secretary, the study PI or delegate, members of the trial Steering Committee, and other study or regulatory personnel as deemed appropriate by the DSMB chair or study PI. The Open Session provides an opportunity for interaction between the study team and DSMB. DSMB members may ask questions or seek information relevant to study conduct or interim monitoring. The PI or their delegate will provide a general study update during the Open Session, including potentially study progress, adverse event status, enrollment statistics including baseline characteristics of masked trial groups, enrollment and follow-up performance, new information relevant to the study conduct or execution, and any concerns or questions about which DSMB input is sought. The study PI or delegate will also discuss any proposed modifications to the trial protocol or trial documents offered for DSMB review and approval. Any study data included in the Open Session report will be in aggregate form or masked as to treatment assignment.

(3) **Closed Session:** As needed, a closed session will be held and attended by the DSMB Chair, DSMB Members, and required study personnel (generally an unblinded study statistician or statisticians). The purpose of the Closed Session is to allow review of confidential data, including grouped safety data, grouped data for any interim efficacy analyses, discussion of DSMB findings and recommendations, and obtain formal agreement by voting. If the DSMB does not receive unmasked data, the DSMB will discuss and decide during each meeting whether to remain masked to the treatment assignments. The unblinded study statistician should be able to provide treatment assignments immediately should the DSMB wish to be unmasked during a meeting.

(4) **Executive Session:** The DSMB may hold an executive session in which only the DSMB members (potentially excluding *ad hoc* DSMB members) are present in order to discuss study issues independently.

(5) **Concluding Session:** A brief meeting may be held between the DSMB Chair and the PI or delegated member of the trial steering committee to provide a summary of the preliminary recommendations and provide an opportunity for participants to clarify the recommendations. At the discretion of the DSMB Chair, other members of the trial steering committee or study team may be invited to attend this session.

If the closed or executive sessions occur on a conference call, steps will be taken to ensure that only the appropriate participants are on the call, and to invite others to re-join the call only at the conclusion of the closed session.

10. Expedited Adverse Event Reporting

Adverse event definitions and reporting timelines are documented in the data and safety monitoring plan or protocol. Adverse events requiring expedited reporting (as defined in Data and Safety Monitoring Plan) will be sent to the DSMB Chair, or DSMB members designated by the DSMB chair with expertise in critical care and septic shock within 7 calendar days of learning of the event. DSMB chair will respond to VASSPR Study Pis with recommendations within 14 days.

11. Reports of DSMB Deliberations

- **Full Summary and Recommendations:** The DSMB Chair, in conjunction with the ES, is responsible for the accuracy and transmission of DSMB meeting summary to the entire Board within 14 calendar days of the meeting. The summary must be signed by the DSMB Chair and will include reports of the closed session and executive session if an executive session was held. The unblinded study statistician may receive the full summary as decided by the DSMB chair on a meeting-by-meeting basis.

- **Blinded Summary and Recommendations:** A version of the full DSMB summary and recommendations edited so as not to expose the investigators to confidential or unblinded information and signed by the DSMB Chair, will be sent to the investigators within 14 calendar days after the meeting. These summary minutes and recommendations at minimum include a statement as to whether the study is approved to continue as planned, any requests for additional data, and response of the investigators to prior recommendations. Requests for additional data from the investigators or DCC/statistician should include an expected due date.

  If the DSMB does not identify any safety or other protocol-related concerns, the Summary Report will state that:
  - A review of outcome data, adverse events, and information relating to study performance (e.g., data timeliness, completeness, and quality) across all study sites took place on a given date;
  - No safety concerns were identified;
  - A review of recent literature relevant to the research took place;
  - The study remains in clinical equipoise; and
  - The DSMB recommended that the study continue without modification of the protocol or informed consent.
  - The DSMB summary report will also include language that efficacy data were evaluated (for the interim analysis meeting only)

  DSMB recommendations will be submitted to the responsible IRB by the study PI or their delegate.

- **Action plan:** If required, the study's follow-up plans and/or response to DSMB recommendations will be submitted to the DSMB within 14 calendar days after the DSMB meeting.

12. Statistical Monitoring Guidelines

The DSMB will review the adequacy of the statistical monitoring plan. The final plan, whether part of a research protocol or separate document, will be considered an appendix to this Charter. The DSMB will

review the statistical monitoring procedures that will be followed to guide recommendations about termination or continuation of the trial based on interim assessment of safety-related events.

Appendix A — History of Changes to DSMB Charter

Date	Charter version modification took effect	Description
9 Jun 2023	2.0	Add interim efficacy analysis (Section 9)<br>Add planned DSMB meeting for interim efficacy analysis (Section 8)<br>Include interim efficacy results in relevant DSMB report (Section 11)<br>Shorten reporting timeline for adverse events requiring expedited reporting (Section 10)
9 Jan 2024	3.0	Revise planned duration of trial (Section 9)<br>Revise timing of interim efficacy analysis (Sections 2, 8, and 9)<br>Add DSMB role to provide input regarding trial duration extension and/or sample size expansion (Section 2)<br>Clarify timing of DSMB review of statistical analysis plan (Section 8) planned study duration
28 Oct 2024	4.0	Update person in role of DSMB executive secretary (Appendix B)

Appendix B — DSMB Voting Members and Personnel

DSMB member name	DSMB Role	Area(s) of expertise	Title & Institution	Email
Todd Rice, MD, MSc	Chair	Pragmatic Trials<br>Critical Care<br>Trial Ethics<br>Trial Safety Monitoring	Associate Professor of Medicine, Division of Allergy, Pulmonary, and Critical Care Medicine<br><br>Medical Director, Human Research Protections Program (IRB)<br><br>Vanderbilt University	todd.rice@vumc.org
Alison Bateman-House, MPH, PhD	Member	Ethicist	Assistant Professor, Department of Population Health, New York University Grossman School of Medicine	Alison.Bateman-House@nyulangone.org
Michael Harhay, PhD, MPH	Member	Biostatistician<br>Trial design	Assistant Professor of Epidemiology and Medicine, University of Pennsylvania School of Medicine	mharhay@pennmedicine.upenn.edu
Crystal North, MD, MPH	Member	Critical care	Assistant Professor of Medicine, Division of Pulmonary and Critical Care Medicine, Harvard Medical School<br><br>Assistant Physician, Department of Medicine, Massachusetts General Hospital	cnorth@mgh.harvard.edu
Christina Barkauskas, MD	Member	Mechanical vent expert	Assistant Professor of Medicine, Division of Pulmonary, Allergy, and Critical Care Medicine, Duke University School of Medicine	christina.barkauskas@duke.edu
Tiaura Webb	Executive secretary	DSMB administration	Executive Assistant, ICU Operations, Intermountain Healthcare	tiaura.webb@imail.org
